# Cubozoan genome illuminates functional diversification of opsins and photoreceptor evolution

**DOI:** 10.1038/srep11885

**Published:** 2015-07-08

**Authors:** Michaela Liegertová, Jiří Pergner, Iryna Kozmiková, Peter Fabian, Antonio R. Pombinho, Hynek Strnad, Jan Pačes, Čestmír Vlček, Petr Bartůněk, Zbyněk Kozmik

**Affiliations:** 1 Department of Transcriptional Regulation, Institute of Molecular Genetics, Videnska 1083, Prague, CZ-14220, Czech Republic; 2 Department of Cell Differentiation, Institute of Molecular Genetics, Videnska 1083, Prague, CZ-14220, Czech Republic; 3 Department of Genomics and Bioinformatics, Institute of Molecular Genetics, Videnska 1083, Prague, CZ-14220, Czech Republic

## Abstract

Animals sense light primarily by an opsin-based photopigment present in a photoreceptor cell. Cnidaria are arguably the most basal phylum containing a well-developed visual system. The evolutionary history of opsins in the animal kingdom has not yet been resolved. Here, we study the evolution of animal opsins by genome-wide analysis of the cubozoan jellyfish *Tripedalia cystophora*, a cnidarian possessing complex lens-containing eyes and minor photoreceptors. A large number of opsin genes with distinct tissue- and stage-specific expression were identified. Our phylogenetic analysis unequivocally classifies cubozoan opsins as a sister group to c-opsins and documents lineage-specific expansion of the opsin gene repertoire in the cubozoan genome. Functional analyses provided evidence for the use of the Gs-cAMP signaling pathway in a small set of cubozoan opsins, indicating the possibility that the majority of other cubozoan opsins signal via distinct pathways. Additionally, these tests uncovered subtle differences among individual opsins, suggesting possible fine-tuning for specific photoreceptor tasks. Based on phylogenetic, expression and biochemical analysis we propose that rapid lineage- and species-specific duplications of the intron-less opsin genes and their subsequent functional diversification promoted evolution of a large repertoire of both visual and extraocular photoreceptors in cubozoans.

Many animals sense light cues for vision and nonvisual photoreception. Light is captured by an opsin-based photopigment in a photoreceptor cell and leads to a cellular light response through a G protein-mediated phototransduction cascade^1^,^2^. Opsins are members of the G protein-coupled receptor (GPCR) superfamily; proteins with seven transmembrane helices that are involved in a diverse set of signaling functions. Within the GPCR superfamily, the opsins form a large monophyletic subclass of proteins characterized by a lysine in the seventh transmembrane helix that serves as the attachment site for the chromophore. Functional opsin proteins covalently bind a chromophore, gaining photosensitivity. Opsins are essential molecules in mediating the ability of animals to detect and use light for diverse biological functions and have been discovered in a wide variety of tissue and cell types, signaling through multiple pathways, and carrying out functions beyond image formation^1^,^3^.

Phylogenetic analysis has indicated that four major opsin monophyletic groups can be recognized^1^,^3^,^4^,^5^. The first group, is comprised of the c-type opsins, the vertebrate visual (transducin-coupled) and non-visual opsin subfamily, the encephalopsins, pinopsins, paprapinopsins, parietopsins and tmt-opsin subfamily and the invertebrate ciliary opsins. The second group, Cnidopsins, is a group consisting of all cnidarian opsins, except for so called Nematostella group 1 and Nematostella group 4 opsins, whose phylogenetic position is still unresolved^4^,^6^. Cnidopsins are exclusively found among cnidarians and not present in any other phyla. The third so-called ‘r-type’ group consists of Gq-coupled invertebrate visual opsins and vertebrate and invertebrate melanopsins and Group 4 opsins contain an assortment of relatively poorly characterized opsin types including, neuropsins, peropsins, and a mixed group of RGRs (retinal G-protein coupled receptors)^7^,^8^,^9^,^10^. The distribution of the opsins into these four major groups is supported by analyses of intron arrangements and insertion/deletion events^3^ and all groups contain genes found in multiple tissue locations (*e.g.* photoreceptor cells (PRCs) and/or other tissues). Two recently published analyses of opsin phylogeny by Feuda *et al.*^6^,^11^ have shown that in contrast to the findings of a majority of other studies^3^,^5^,^10^, Cnidarian opsins, including the Nematostella group 1 and Nematostella group 4 opsins, might not be of monophyletic origin, but rather can be divided into three groups, each more closely related to either the c-, r- or Group 4 opsins.

One of the defining characteristics of opsins are the presence of covalently bound chromophores, most commonly 11-cis retinal, that confer light sensitivity to the visual pigment. The chromophore is attached via a Schiff base linkage to a universally conserved lysine in the seventh transmembrane helix. Upon exposure to light, the chromophore undergoes a photoisomerization event to form all-trans retinal, that in turn drives the activation of the photopigment. Aside from this universally conserved lysine, other important amino acid residues can also be found in opsin primary structures. One example is the so-called ‘counterion’, typically a negatively charged amino acid that is required to interact with and thus raise the pKa of the protonated Schiff base linkage between retinal and lysine, stabilizing the binding of a proton at physiological pH. While vertebrate visual pigments use amino acid-113 as the counterion, position 181 can also be used by a diverse group of opsins containing photoisomerases and Gi/o-coupled pigments, whereas vertebrate melanopsins utilize amino acid position 83^3^. Multiple lines of evidence support the hypothesis that amino acid substitutions in the fourth cytoplasmic loop of duplicated opsins were involved in the origins of novel opsin-G protein interactions^5^. Residues 310-312, encompassing the so-called tripeptide region, were formerly demonstrated by site-directed mutagenesis to mediate opsin-G protein interactions in ciliary opsins^12^ and these data were later supported by correlation analyses^5^.

Box jellyfish belong to the phylum Cnidaria, arguably the most basal phylum containing a well-developed visual system. It is well known that light affects many behavioral activities of cnidarians, including diel vertical migration, responses to rapid changes in light intensity and reproduction^13^. Their phylogenetic position, simple nervous system and elaborate set of eyes^14^ make their visual system of key importance for understanding the early evolution of vision, and also for understanding the biology of box jellyfish^15^,^16^,^17^,^18^. The eyes of box jellyfish share many features with those of vertebrates. Morphologically, they are similar by overall design (lens, retina, ciliary photoreceptors)^14^,^19^, and recently, characterization of some molecular components has suggested that the box jellyfish visual system is more closely related to vertebrate than to invertebrate visual systems^20^,^21^,^22^,^23^. Photoreceptive organs in Cnidaria have diverse structures, not only between species^24^ but within the same species. The cubozoan jellyfish investigated in this study, *Tripedalia cystophora*, has four equally spaced sensory structures called rhopalia, dangling from a stalk and situated within open cavities surrounding the bell. Each rhopalium has six separate eyes. There are two complex, lens-containing eyes (upper lens eye – ULE, and lower lens eye – LLE), one larger than the other, situated at right angles to each other, and two pairs (one pit-shaped, one slit-shaped) of simple ocelli comprising photoreceptors on either side of the complex eyes^25^,^26^. As the visual fields of individual eyes of the rhopalium partly overlap, *T. cystophora* (as well as other Cubomedusae) has an almost complete view of its surroundings. The lens-containing *T. cystophora* eyes have sophisticated visual optics, similar to molluscs and vertebrates^14^,^19^. Two opsin genes have so far been identified in cubozoans, one in *T. cystophora*^22^, and one in the related species *Carybdea rastonii*^27^. Expression of both these opsins has been detected in eyes of the corresponding species^22^,^27^. *C. rastonii* opsin was furthermore shown to transfer the light stimulus via the Gs signaling pathway^27^.

In the present work, we characterize a complement of 18 opsin genes identified in cubozoan jellyfish *T. cystophora* by the whole-genome analysis. Based on phylogenetic, expression and biochemical analysis we propose that rapid lineage- and species-specific duplications of the intron-less opsin genes and their subsequent functional diversification promoted evolution of both visual and extraocular photoreception in cubozoans.

## Results

### A large complement of opsin genes are present in *T. cystophora* genome

In addition to previously annotated *T. cystophora* c-opsin^22^ we identified 17 other *Tripedalia* opsins (Tcops) sequences. Among those novel sequences, we were able to identify the ortholog (93% sequence identity) of the previously investigated *C. rastonii* opsin (Caryb)^27^, designated here in *T. cystophora* as Tcop13 (Fig. S1). Complete coding sequences for all these opsins were obtained by Genome Walking (GenomeWalker, Clontech). All of the eighteen *T. cystophora* opsins are intron-less genes, which show overall sequence homology with other cnidarian opsins as well as to bilaterian rhodopsins. The conserved lysine to which the chromophore 11-cis-retinal binds was found in each of the cloned opsins, suggesting that they are indeed used for photoreception. Next, we investigated the three potential counterion sites at amino-acid position 83, 113, 181 (numbered according to bovine rhodopsin) within the cnidopsins (Fig. S2). Negatively charged amino acids (either glutamic acid/E or aspartic acid/ D) at position 83 was found in more than 50% of the identified cnidopsins, with more than 95% having E/D at position 181. Intriguingly, E/D residues at position 113 were only found in four of the identified *T. cystophora* opsins. These were, Tcop8, Tcop12, Tcop15, and Tcop16 (Fig. S2 – red box). However, it is important to note that E/D counterions at position 113 have, to date, not been found in any opsins identified outside of chordates. Next, we investigated the identity of residues 310–312 within all the Tcop sequences. The tripeptides tended to be conserved among closely related cnidopsin groups of each species (Fig. S2) but are apparently not conserved across the cnidarian lineages. In summary, our collective data indicate that a large repertoire of diverse opsins is present in the cubozoan genome, some of which have some intriguing sequence similarities to vertebrate opsins.

### Phylogenetic relationships of cubozoan opsins within the opsin gene family

**Figure 1:**
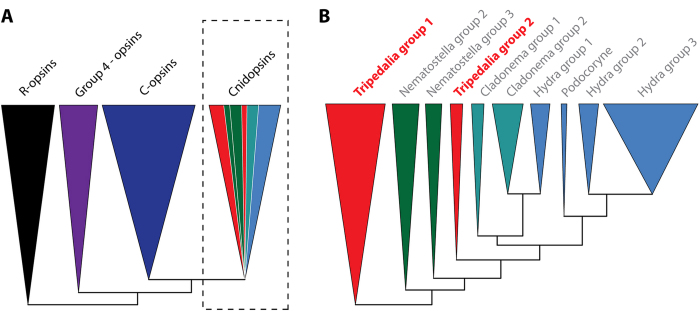
Schematic representation of the opsin phylogenetic analysis of a large set of opsin genes including the cubozoan dataset. **A**) Phylogenetic analysis performed in this study recovered previously described four major opsin lineages – r-opsins, c-opsins, group 4 opsins and cnidopsins. Herein the C-opsins and cnidopsins form sister groups. (For details see Fig. S3), **B)** Detailed inspection of cnidopsin branch indicates extensive gene duplication and lineage-specific expansion of cnidarian opsins. (For details see Fig. S4).

To investigate the relationship between the newly identified Tcops and other known metazoan opsins, we inferred a molecular phylogenetic tree by the maximum likelihood method from a set of 779 opsin protein sequences. Our phylogenetic analysis of this large and diverse set of opsin sequences recovered the four major lineages described in earlier studies^3^,^4^,^5^,^10^, *i.e.* the c-type opsins, the cnidopsins, the r-type opsins, and group 4 opsins. The relationships among the four major lineages in our analyses correlated with those proposed in other recent studies of opsin evolution^4^,^5^,^10^, however, the statistical support for some of the relationships was weak. Due to such weak branch support, we were unable to exclude the possibility that group 4 and r-type opsins cluster together as sister groups, opposing the c-type opsin and cnidopsin subgroups as has been suggested by Porter *et al.*^3^ (based on their phylogeny and the presence of functional unity as bistable pigments of arthropod/cephalopod visual r-type opsins and chicken group 4 neuropsin^28^,^29^). We found that the relationship between cnidopsins and the c-type opsin subfamily was most strongly supported. All cnidarian opsins except for *Nematostella* group 1 and *Nematostella* group 4 opsins fell within the cnidopsins, group and as was shown by Suga *et al.*^4^, the phylogenetic positions of these two groups remained unclear even after more precise Maximum-Likelihood analyses. In the phylogenetic tree of the opsin family, all identified Tcops fell into the cnidopsins subfamily (Fig. 1A, Fig. S3), consistently clustering with the hydrozoan opsins^30^. The cnidopsin group, composed entirely of cnidarian opsins, is the only group lacking representation of broad taxonomic diversity from the major animal phyla. The phylogenic trees presented here (Fig. 1B, Fig. S4) and elsewhere^3^,^4^ are indicative of extensive gene duplications (and diversifications) in each of the cnidarian lineages after their initial split. This latter point is exemplified by the case of Tcops that form two distinct phylogenetic groups: Tc-group 1 and Tc-group 2 (Fig. 1B).

In summary, our phylogenetic analysis unequivocally classifies cubozoan opsins as a sister group to c-type opsins and documents lineage-specific expansion of the opsin gene repertoire in the cubozoan genome.

### Functional diversification of opsins in *T. cystophora*: evidence of an apparent dichotomy in G protein-coupled signaling

**Figure 2:**
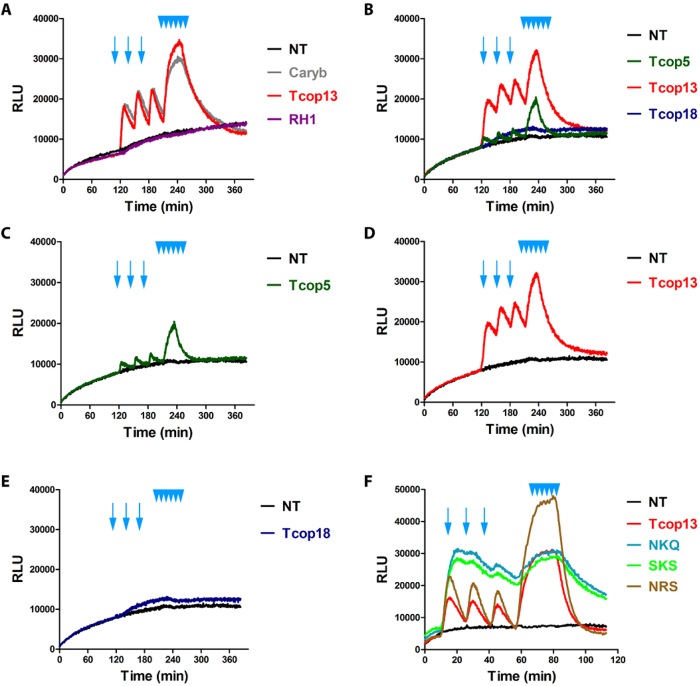
Opsin-Gs-cAMP assay. Light activation of opsin-Gs-cAMP pathway by selected opsins. GloSensor™-20F cAMP HEK293 cells (Promega) were transfected with expression vectors encoding genes for different opsins, treated and stimulated with light, as described in Materials and Methods. Arrows represent simple light pulses, multiple arrowheads represent repeated stimulation. Each graph represents a mean of triplicates for every sample. **A)** Previously reported Gs-cAMP pathway stimulating opsin from *C. rastonii* (Caryb)^27^ showed ability to increase the cAMP level in our setup (visualized with cAMP-dependent luciferase activity). The exact homolog of Caryb from *T. cystophora* Tcop13 showed a highly similar response in our assay. Opsin RH1 from medaka, expected to signal via Gt leading to cGMP decrease, showed no change in luciferase activity. **B)–E)** Examples of different Tcop light responses. Tcop5 showed faster and weaker activation of the Gs-cAMP pathway than Tcop13. Tcop18 did not activate the Gs-cAMP pathway. **F)** Analysis of tripeptide activity in Tcop13 was performed. Tcop13 tripeptide HKQ was replaced with tripeptides NKQ, SKS and NRS (originally found in opsins Tcop1 or bovine rhodopsin, Tcop14 and Tcop18 – none of which activated the Gs cascade). Tripeptide mutation did not disrupt Gs activation by Tcop13, but influenced length or sensitivity of Tcop13 response to light stimulation. NT – non-transfected cells used as negative control; Caryb – signal for cells transfected with a vector expressing opsin from *C. rastonii*, used as positive control; RH1 – signal for cells transfected with a vector expressing opsin RH1 from medaka fish *Oryzias latipes*, used as negative control; Tcop5, Tcop13, Tcop18 – signal for cells transfected with vectors expressing opsins from *T. cystophora* - Tcop5, Tcop13 or Tcop18, respectively; NKQ, SKS, NRS – Tcop13 original tripeptide HKQ replaced with tripeptides NKQ, SKS or NRS.

Sequence analysis and phylogenetic classification provided an important insight into the evolutionary history and possible relationships among opsins. However, these sequence-based approaches do not answer the question whether one or more signaling pathways are being used by *T. cystophora* opsins and do not permit the drawing of conclusions regarding which signaling pathway is coupled to any particular opsin. In order to get a deeper insight into the functional diversification of opsins identified in *T. cystophora* we used a GloSensor™ cAMP HEK293 cell based Gs protein-coupled signaling assay^31^ to investigate biochemical properties of all Tripedalia opsins (for description see Material and methods). We used *C. rastonii* opsin (Caryb), shown to activate the Gs-cAMP pathway^27^, as a positive control. As heterologous protein expression in cell lines may sometimes prove difficult or even impossible^32^, we first checked the expression of the individual opsin genes in GloSensor™ cAMP HEK293 cells by immunofluorescent labeling. The staining revealed that all examined opsins were expressed in GloSensor™ cAMP HEK293 cells and at comparable levels. Moreover the sub-cellular fluorescent signal for opsin was consistently detectable on the cell membranes (Fig. S5 and data not shown), thus confirming successful expression of the Tcop genes. The luciferase activity in GloSensor™ cAMP HEK293 cells, transfected with individual opsin constructs and pre-incubated in the dark with 9-cis retinal, was determined before and after repeated light stimulations (Fig. 2). Light stimulation of cells was in specific cases immediately followed by increased luciferase activity reaching a maximum after several minutes (Tcop5) or 10 minutes (Tcop13, Caryb) and remaining constant for several minutes before decreasing to the basal levels observed prior to illumination (Tcop5) or slightly higher (Caryb; Tcop13). Comparison of the previously characterized Caryb with its ortholog from *T. cystophora*, Tcop13, revealed that both opsins show similar light responses (Fig. 2A). In contrast, medaka (*Oryzias latipes*) opsin RH1, expected to signal via a distinct G protein-coupled pathway (Gi, leading to a cGMP decrease), elicited no increase of luciferase activity in our assay (Fig. 2A), being expressed at comparable levels to those of *T.cystophora* opsins (Fig. S5). Furthermore, no light-dependent stimulation of the Gs protein-coupled assay was detected using the invertebrate r-opsin gene, expected to function via Gq signaling (data not shown). Our assay was, therefore, highly specific for opsins signaling via the Gs-cAMP pathway, but was insensitive to signaling via Gi or Gq. We performed the light response assay several times for the entire set of *T. cystophora* opsins. Of all the opsins examined, only Tcop5 and Tcop13 activated the Gs-cAMP signaling pathway (Fig. 2B–E and data not shown). No convincing light induced opsin-Gs-cAMP response, similar to that of Caryb, Tcop5 and Tcop13, was detected for other Tcops. Tcop5 and Tcop13 sustained enhancement of G protein-coupled pathway signaling after repeated light stimulation. However, we noticed a conspicuous difference in the light response between these two opsins. Tcop5 responded to light faster but with lower intensity (Fig. 2C), whereas the response of Tcop13 was considerably slower, ultimately reaching higher signaling values (Fig. 2D).

To better understand the molecular features of cnidopsins, we focused on the role of the tripeptide in cnidopsin signaling. As stated above, the tripeptide is important for the contact between bovine rhodopsin and its G protein^12^. Accordingly, we replaced the HKQ tripeptide region in Tcop13 with either the tripeptides NKQ, SKS and NRS, originally found in Tcop1 (or bovine rhodopsin), Tcop14 and Tcop18, respectively, none of which activated the Gs signaling cascade in our assay. We expected to observe a loss of Gs cascade activation resulting from the tripeptide mutation. Surprisingly, we found that the tripeptide mutation in Tcop13 did not disrupt Gs activation; rather, it influenced the dynamics of the response to the light stimulation. Specifically, the introduction of the tripeptides NKQ and SKS led to an enhanced and prolonged response, while the introduction of NRS variant caused a massive light response after both single and repeated stimulation (Fig. 2F). Our data show that tripeptide mutation in cnidopsins contributes to subtle tuning of the opsin response to light stimulation, rather than influencing Tcop-G protein coupling *per se*.

In summary, Gs-cAMP signaling characterized only a small set of *T. cystophora* opsins, indicating that the majority of cubozoan opsins likely signal by a distinct and as of yet unidentified signaling pathway. Moreover, the highly sensitive two-dimensional functional assay used here (measuring time response as well as response intensity) uncovered subtle differences among individual opsins, suggesting possible fine tuning for specific photoreceptor tasks.

### Phototactic behavior of *T. cystophora* medusa is dependent on Gs signaling

**Figure 3:**
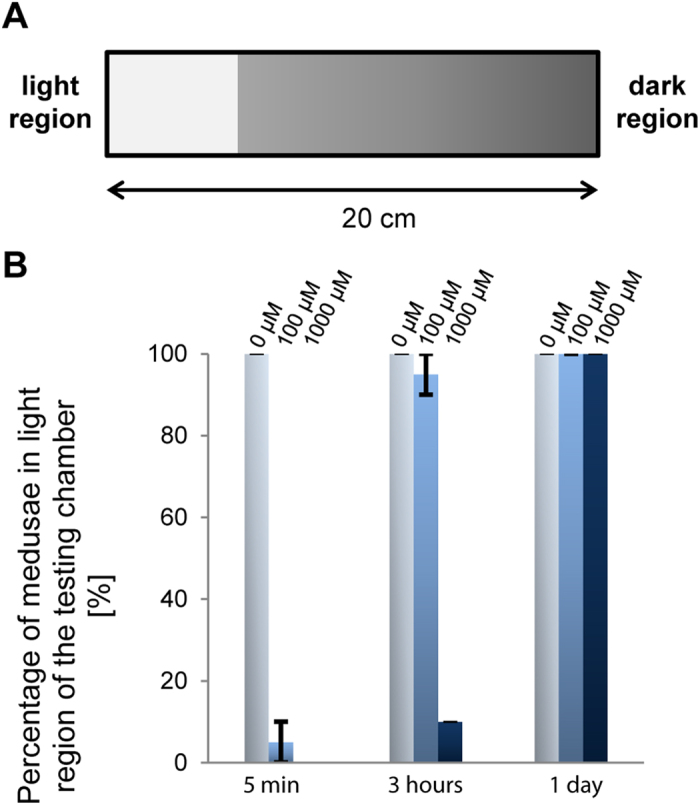
Test of *T. cystophora* medusa phototaxis after NF449 treatment. **A)** Schematic representation of the testing chamber. **B)** Statistical analysis of the light response of *T. cystophora* after NF449 (Gαs inhibitor) treatments (0 μM, 100 μM, 1 mM). Bars represent the percentage of phototactic medusae in given time point.

Caryb opsin present in retinas of lensed eyes of *C. rastonii* was previously shown to transfer the light stimulus via the Gs signaling pathway^27^. To investigate whether Tc-group 2 (especially Tcop13), that signal via Gs (see above), serve as important visual pigments in *T. cystophora*, we performed a phototaxis behavioral assay in the absence or presence of the pharmacological compound NF449. NF449 was originally identified as a selective suppressor of the Gs signaling pathway, with limited effect on the prototypical Gi/Go- and Gq-coupled receptors pathways^33^. Positive phototaxis in *T. cystophora* medusa was significantly decreased after treatment with NF449. Although, a variable response was detected in response to white light depending on NF449 concentration and timing of the treatments (Fig. 3 + video), most probably due to reversible inhibition of the Gs signaling pathway by NF449. The number of treated animals exhibiting a phototactic response 5 minutes after the treatment was: 5 ± 5% in samples treated with 100 μM NF449 and 0% in samples treated with 1mM. The number of responding medusae treated with 100 μM NF449 after 3 h rose to 95 ± 5% and the number of medusae treated with 1 mM NF449 rose to 10%. However, after 24 hours the decrease in photosensory response was no longer present. We observed 100% phototactic response in untreated animals (0 μM NF449) after 5 min, 3 h, 24 h intervals (Fig. 3B). Thus, the pharmacological inhibition of Tc-group 2 opsins abrogates positive phototactic movement of cubozoan medusa.

### Opsin gene expression analysis reveals both redundancy and specialization

**Figure 4:**
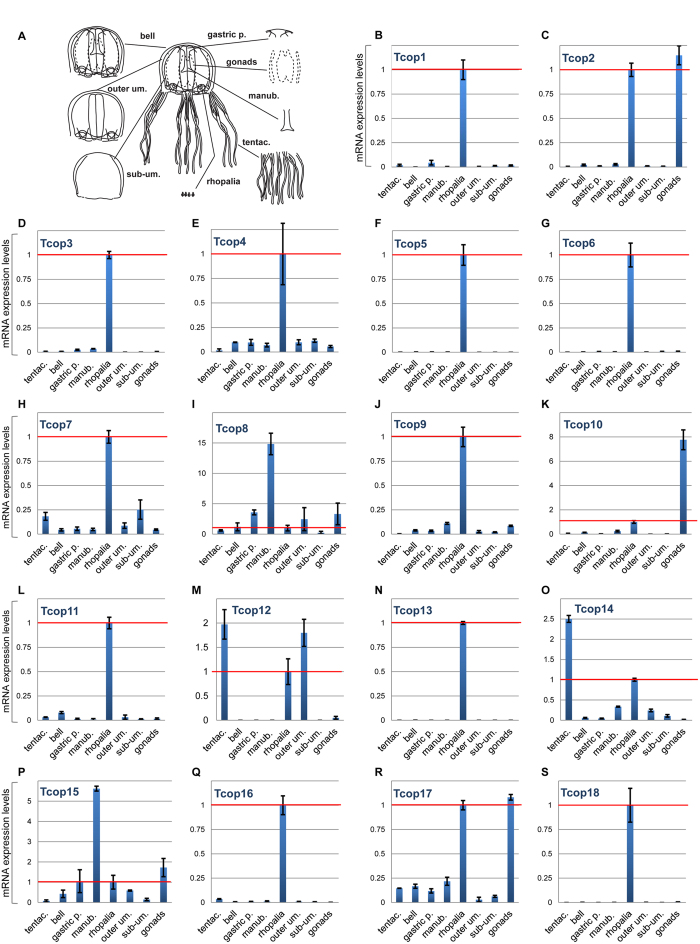
mRNA expression levels of *T. cystophora* opsins in dissected body parts of adult jellyfish. **A)** For real-time PCR analysis, medusae were dissected into eight body parts: tentacles (tentac.), manubrium (manb.), male gonads, gastric pouch (gastric p.), bell, outer umbrella (outer um.), sub-umbrella (sub-um.) and rhopalia.** B–S)** mRNA expression level of opsins for each dissected body part relative to the rhopalium expression (1.0 – red line). y – axis: relative mRNA expression level, x – axis: analyzed body parts.

The large complement of opsins found in the *T. cystophora* genome raises the possibility of their differential tissue-specific or stage-specific utilization. To investigate the expression patterns of *T. cystophora* opsins, we first analyzed mRNA isolated from different jellyfish life stages and dissected adult tissues by real-time qRT-PCR analysis. The normalized expression levels, relative to Rpl32 levels, of specific opsin genes in each dissected adult body part was calculated relative to that observed in the rhopalium (set at 1.0) and plotted (Fig. 4). Relevant opsin expression data were also represented as a heat map showing z-score of Tcops expression in different *T. cystophora* body parts. (Fig. S6A). All Tcop genes were found to be expressed at the mRNA level in the rhopalium. Moreover, for the majority of opsins (Tcop1, Tcop3–7, Tcop9, Tcop11, Tcop13, Tcop16, Tcop18), the rhopalium was the tissue exhibiting the highest detected expression. Other opsins were most highly expressed in the male gonads (Tcop2, Tcop10, Tcop17), tentacles (Tcop12, Tcop14) or manumbrium (Tcop8, Tcop15). For a better gene-to-gene comparison within the rhopalium, the results were plotted separately (Fig. S7). The expression data in the adult tissues identified common/over-lapping sites of expression most probably reflecting a common gene origin. Nevertheless, a clear tendency for specialization was apparent as very large differences in relative expression levels and/or unique sites of expression were detected.

**Figure 5:**
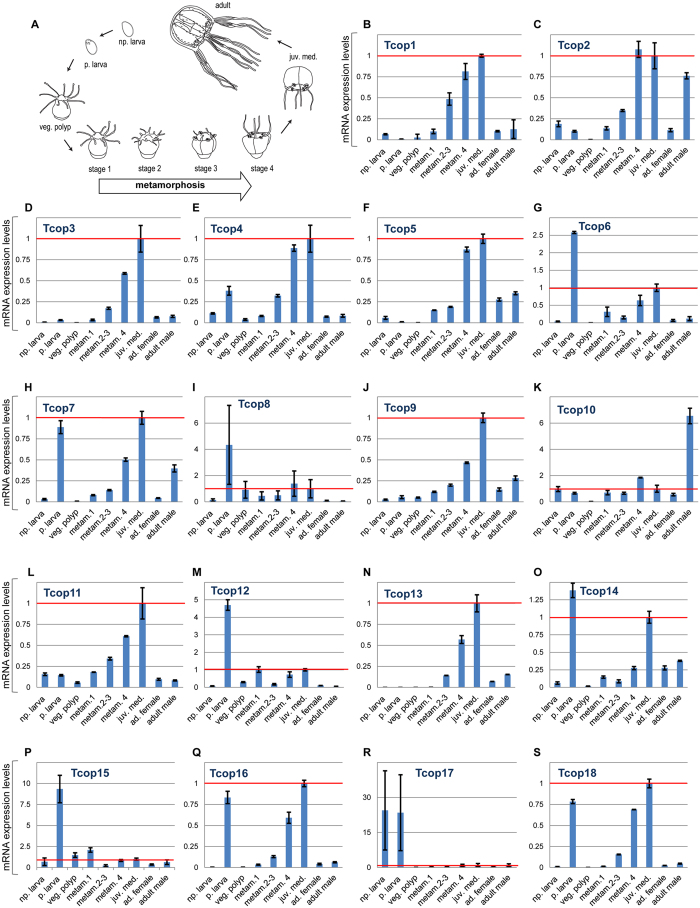
mRNA expression levels of *T. cystophora* opsins in different life stages. **A)** For real-time PCR analysis, animals of nine subsequent life stages were collected: non-pigmented larva (np. larva), pigmented larva (p. larva), vegetative polyp (veg.polyp), three polyp-to-medusa metamorphosing stages (metam1, 2–3, 4), juvenile medusa (juv. med.), adult female (ad. female) and adult male. **B-S)** mRNA expression levels of opsins for each life stage relative to the juvenile medusa expression (1.0 – red line). x – axis: analyzed stages, y – axis: relative mRNA expression level.

Next, we investigated opsin gene expression during the life cycle of *T. cystophora*. To this end, mRNAs from non-pigmented larva, pigmented larva (larval eye-containing stage), vegetatively grown polyp, four stages of a metamorphosing polyp (stages 3 and 4 containing a developing rhopalia) and medusae were isolated and subjected to qRT-PCR. The expression levels of all individual opsins for each *T. cystophora* life stage relative to the juvenile medusa expression (set as 1.0) were then calculated (Fig. 5 and Fig. S6B). The results revealed two consistent features. Firstly, opsins whose expression was detected in the adult rhopalium, sharply increased their expression during the polyp metamorphosis, coincident with the emergence of the developing rhopalia structure. Secondly, many Tc-group 1 opsins were highly expressed in the pigmented (eye-containing) larval stage, contrasting with the expression of Tc-group 2 opsins (established as Gs-coupled receptors with a major functional role in the adult lens-containing eye, see above), that were notably absent at this stage.

**Figure 6:**
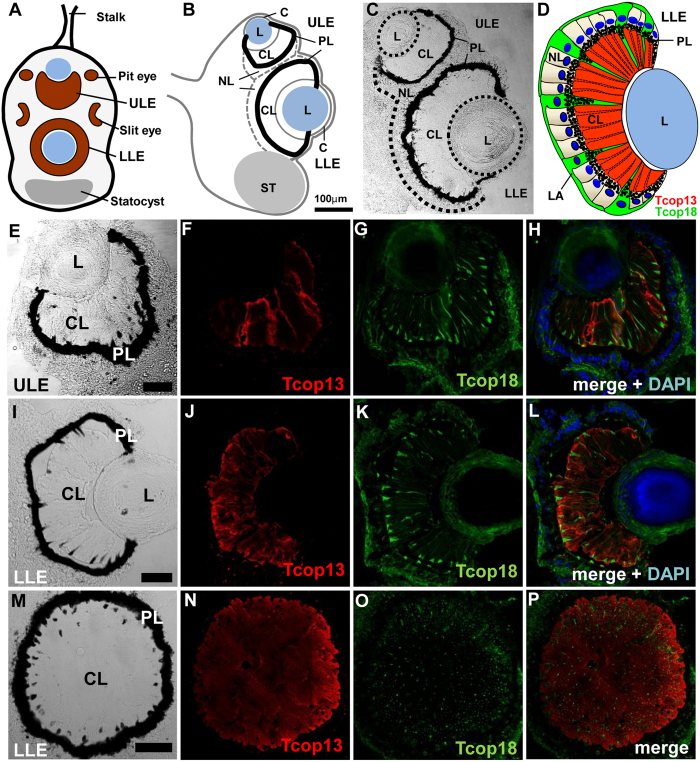
Visual organs of *T. cystophora* and immunohistochemical localization of Tcop13 and Tcop18. **A)** Schematic diagram of the rhopalium. The large (LLE) and small (ULE) complex eyes lie along the medial line, while the pit and slit ocelli are paired laterally. **B)** Schematic diagram of rhopalium sagittal plane (adapted from O´Connor 2009). **C)** Sagittal section through the rhopalium. Upper (ULE) and lower (LLE) lens eyes contain the typical components of camera-type eyes: a cornea (C), a lens (L), and a retina consisting of a ciliary layer (CL), a pigment layer (PL) and a neural layer (NL). St – statocyst, S – stalk. **D)** Schematic representation of the lens eye retina. The ciliary layer (CL) is dominated by the ciliary segments of type-B receptor cells (red). Scattered among the type-B receptor cells are the cone-shaped projections of type-A photoreceptor cells (green). In the neural layer (NL), both receptors types have their cell bodies with nuclei (dark blue); only type-A receptor cell bodies are positive for opsin signal. Projections of type-A photoreceptor cell bodies create a compact layer (LA) surrounding the whole retina. **E–H**) Confocal images of immuno-histochemical staining for Tcop13 (red), Tcop18 (green), DAPI (blue) in the upper lens eye (ULE). **I-L**) Large camera-type eye (LLE) retina longitudinal section. **M–P**) Large camera-type eye retina transverse section. (Scale bars: 50 μm).

**Figure 7:**
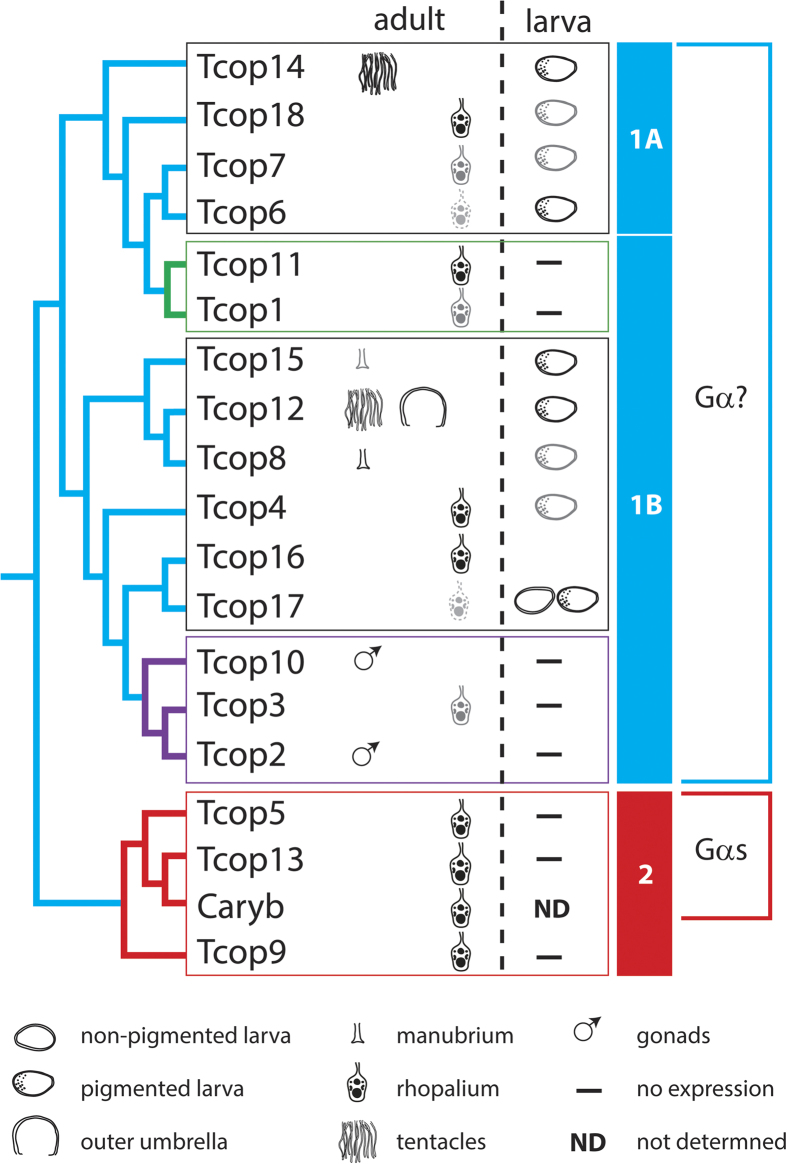
Schematic representation of opsin expression patterns according to their phylogenetic relationship. *T. cystophora* opsins can be classified into two groups, a probable more ancient Tc-group 1 opsin, with a broader expression pattern, and Tc-group 2 – rhopalium-specific opsins. The size and shade intensity of the symbols corresponds with the level of expression. Green coloured box and branches represent rhopalia specific Tc group 1A opsins. Purple coloured box and branches represent male specific Tc group 1B opsins. Red coloured box and branches represent rhopalia specific Tc-group 2 opsins.

To gain further insight into the possibly diverse roles of Tc-group 1 and Tc-group 2 opsins in the various cubozoan eyes (Fig. 6A–C), we also analyzed the expression of key representatives of each opsin type by immunohistochemical staining (IHC) *in situ*. Accordingly, we generated a specific antibody against Tcop13 and performed co-staining with another antibody raised against Tcop18^22^ on cryo-sectioned rhopalia. We found that both Tcop13 and Tcop18 were found to be co-expressed in the retinas of *T. cystophora* ULE and LLE in distinct patterns (Fig. 6D–P). We also discovered that *T. cystophora* retinas contain at least two distinct photoreceptor types: ciliary photoreceptor type-A that express Tcop18 not restricted only to the cilia but rather expressed more broadly within the whole cell body (Fig. S8) plus ciliary photoreceptor type-B (expressing Tcop13 in the receptor cell cilia). Both opsins were also distinctly expressed in the developing lens eyes of the newly metamorphosed *T. cystophora* medusae; however, only Tcop18 was detected in the developing eyes of another Carybdeid jellyfish, *Alatina marsupialis* (Fig. S9). Another difference in the apparent utilization of Tc-group 1 and Tc-group 2 opsins was revealed by further analysis of their expression in the two lesser eye types, slit and pit eyes, whose morphology has been thoroughly studied^26^. Only Tcop18 (but not Tcop13) was found to be expressed in the pit and slit ocelli of *T. cystophora*; both types of ocelli thus seem to be formed exclusively from type-A photoreceptors based on the opsin type expression (Fig. S10). However, some molecular features are shared by the PRCs of lesser eye types with those of complex lensed eyes. For example, all PRCs in *T. cystophora* contain two different screening pigments, dark pigment and a white pigment, first described in *Chiropsella bronzie*^24^, that becomes conspicuous in polarization microscopy (Fig. S11). It is important to stress that the Tc-group 1 opsin Tcop18 is the only known opsin to be expressed in the lesser eyes thus far. All phylogenetic, biochemical and gene expression data are schematically summarized in Fig. 7.

## Discussion

### A Scenario for intron-less retrogene-derived cnidarian opsin expansion

The current results highlight distinct features of intron-less genes in vertebrates. It appears that many intron-less genes are evolutionary innovations, so their formation, at least in part, via reverse transcription-mediated mechanisms, could be an important route of evolution of tissue-specific functions in animals^34^,^35^.

The lack of introns is typical for most members of the giant GPCR gene family and it has been proposed that many G-protein-coupled receptors are derived and amplified from a single intron-less common progenitor that was encoded by a retrogene (a DNA gene copied into genome by reverse transcription of an RNA transcript)^36^,^37^. Interestingly the vertebrate rhodopsin GPCRs, that are widespread phylogenetically and abundantly expressed, contain four introns in highly conserved positions^38^. However, most of the cnidarian opsins thus far annotated are intron-less genes, although at least one opsin in anthozoan *Acropora digitifera*, CNOP2, has been characterized with two introns^39^. Astonishingly, the first of these intron matches, in position and phase, with the first intron of bovine rhodopsin (Fig. S12). Such examples of the first introns to be located in cnidarian opsins are moderately short and have conventional GT-AG donor and acceptor splice sites and thus it appears that this intron was already present in an opsin gene present in the last common ancestor of eumetazoa. Accordingly, intron-less opsin genes appear to be a Cnidarian feature, with the original variant of the gene most probably being lost in medusozoans. Similarly, intron-less opsin genes were previously identified in two cephalopod species^40^ and in genome of teleost fish^41^, in both cases probably derived from introns containing opsin genes by retrotransposition. Based on these facts and our data, we propose the scenario (Fig. S13) that cnidarian intron-less opsins are retrogenes derived from an ancient eumetazoan ciliary-like opsin with introns. This hypothesis is supported by the phylogenetic relationship of c-like opsins and cnidopsins (Fig. 1A) and by the fact that both variants of the opsin gene (with or without introns) are still present in basal anthozoa (Fig. S12). Once an intron-less opsin gene was present in cnidarian genome, subsequent rapid lineage- and species-specific duplications resulted in a variety of opsins. This process provided the substrate for the evolution of cnidarian photoreception, be it either extraocularly or in sophisticated cubozoan eyes.

Gene duplications and their subsequent divergence play an important part in the evolution of novel gene functions^42^. Our data show that in *T. cystophora* genome, each of the opsins has been duplicated at least once, and several have undergone multiple rounds of duplication (Fig. S4). Theory suggests that duplicated genes can be lost rapidly^43^, but the spectrally diverse aquatic environment (such as the margins of mangrove lagoons naturally inhabited by *T. cystophora*) could provide strong selective pressures on the opsin genes, and thus, photoreception evolution. Photoreception tuning through opsin sequence evolution might therefore be a result of sensory adaptation to this rich environment of spectral light.

**Figure 8:**
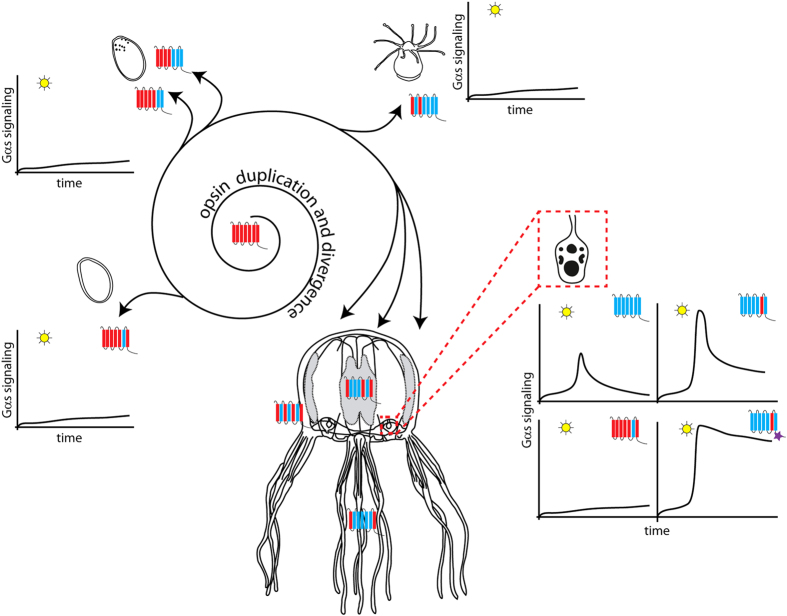
Possible scenario for expansion and functional diversification of opsins in *T. cystophora.* Our data and data from other studies^39^,^60^ show that Cnidarian intron-less opsins might have been derived from an ancient eumetazoan ciliary-like opsin containing introns by retro-transposition. Once anchored in the genome the ancient cnidopsin gene underwent several rounds of duplication, diversification and sensitivity tuning. Individual opsins were thus accommodated for distinct functions in diverse tissue photoreceptors - ocular, extraocular and larval. These opsins differ in stage- or tissue-expression, primary structure and also in subsequent cellular signaling – either via Gs-cAMP pathway or other G-protein pathways. For further information see Discussion.

In both medaka and zebrafish the opsin gene diversity in the genome is high, similarly as in the genome of *T. cystophora*. Subtype opsin genes in medaka and zebrafish are closely linked and are clearly products of local gene duplications^44^. Tandem duplication appears to be the most common mode of opsin gene family expansion in fishes^45^. Gene duplication followed by amino acid substitutions at key tuning sites played an important role in generating a diverse set of fish opsin genes. It is probable that similar mechanisms of opsin gene repertoire expansion occurred in the case of cnidaria (evolutionary convergence), where the opsin genes, being relatively short and intron-less, were even more rapidly duplicated and subsequently functionally diversified (see Fig. 8 for schematical representation).

### Rhopalia-specific opsin expression in *T. cystophora*

Cubozoa have relatively simple nervous systems consisting of a nerve net and a ring nerve. The latter has extensions forming ganglia and connections with the radial nerves and rhopalia. Morphological and electrophysiological studies have shown that a significant part of the CNS of cubomedusae is situated within the rhopalia^25^,^46^,^47^. In addition to numerous ciliated photoreceptors within the retinas of all six eyes, each rhopalium houses over 1,000 neurons of which approximately 500 are retina-associated. Each rhopalium also contains a group of pacemaker neurons that regulate swimming movements through the direct control of neuronal activity in the motor nerve net, and thus individual rhopalium facilitates various behaviors such as obstacle avoidance or light-shaft attraction enabling them to remain in close proximity to prey gathered in beams of light passing through open parts of the mangrove canopy. This behavioral regulation is most probably influenced by the visual input received by each rhopalium^48^.

Based on our mRNA (Fig. 4) and protein (Fig. 6) expression profiles, many of the opsin genes identified here are expressed in rhopalia. Since it is not easy to determine the physiological relevance of a given gene just based on the level of its mRNA expression, we appreciated the finding that Tcop18, with 100 times lower level of mRNA transcripts compared to Tcop13 (Fig. S7), is significantly expressed on protein level (Fig. 6). Based on this fact, we suggest that all Tc-group 2 opsins, plus at least one opsin from each subclass of Tc-group 1 is rhopalium-specific. Moreover, the real-time PCR analysis has revealed that all of the rhopalium-expressed opsins are dramatically up-regulated when the rhopalia are formed during the polyp-to-medusa metamorphosis (Fig. 5). We thus propose that the *Tripedalia* rhopalium is a complex organ integrating and processing multiple light cues, gained though a diverse set of opsins, and transforming these signals into various behavioral responses.

### Retina-specific opsin expression in *T. cystophora*

In addition to the extensive real-time PCR expression analysis, we paid special attention to the IHC analysis of retina specific opsins expression in *Tripedlia* rhopalia. The cubozoan lens-containing eyes have a thin cornea (made of monociliated epithelial cells), a spherical cellular lens, a thin vitreous space, and a hemisphere-shaped everse retina with pigmented photoreceptors of the ciliary type, as judged from their ultra-structural morphology^24^,^49^,^50^.

A previous study identified three types of photoreceptors in the lensed eyes of *T. cystophora* on the basis of differences in the morphology of their sensory cilium and microvillar organization^50^. In contrast, other studies^51^,^52^,^53^ supported the interpretation that there was only a single basic morphological type of photoreceptor in cubozoan lensed eyes. Our IHC data support the first interpretation, showing that there are at least two types of PRC (each with markedly different opsin expression profiles) in the lensed eyes of *T. cystophora*. Both cell types have three distinct segments, giving rise to three retinal layers: 1. a thick layer of receptor-cell cilia formed from type-B PRCs (expressing Tcop13) and cone-shaped projections from type-A PRCs (expressing Tcop18), creating the ciliary layer; 2. a thin pigment layer where both receptor cells types are densely pigmented; 3. a neural layer containing nucleated cell bodies of both types of receptor cells.

The ciliary layer is dominated by the ciliary segments of type-B receptor cells. The cilia extend from the pigment layer to the vitreous space. From the ciliary membrane, microvilli extend, partly as bundles of parallel microvilli and partly as a disorganized tangle (as shown in another cubozoan jellyfish *Ch. bronzie*^24^). The microvilli make up the majority of the volume of the ciliary layer. Scattered among the type-B receptor cells are the cone-shaped projections of type-A photoreceptor cells partially filled with screening pigment granules. These cones run parallel to the ciliary trunks of the type-B sensory cells. In the neural layer, the type-A receptor cells have their cell bodies with nuclei, and they are also positive for Tcop18 protein expression (Fig. 6E–P). Projections of type-A photoreceptor cell bodies create a compact layer surrounding the whole retina.

It has been previously suggested that the lens eye photoreceptors utilize a different photopigment from those of the pit eyes and slit eyes^52^. According to dominant mRNA and protein levels and strict retinal specificity, we consider Tcop13 the main visual opsin of *T. cystophora* complex lens eyes. On the other hand, Tcop18 (also expressed in lens eyes) appears to be the main visual opsin in the lesser eyes. Our IHC data show, that retinas of both eye types (lens and lesser eyes) express different opsin combinations (various combinations of Tcop13 and Tcop18) according to their task (another level of visual tuning). The expression of rhopalium-specific opsins surely does not only involve photoreceptors of the retina, as some of the retina-associated neurons will most probably prove to be photosensitive as well, given our qRT-PCR analysis. This possibility should be resolved in the future by detailed IHC analysis assaying other Tcops expression.

### Tissue-specific and larval opsins

Eyes are not the only means of photoreception in the Cnidarians, as many species lack distinct ocular structures yet exhibit specific photic behaviors. In these animals, photosensitivity is mediated through extraocular PRCs. Extraocular photosensitivity, is widespread throughout the animal kingdom, in both invertebrates and vertebrates^54^,^55^. The extraocular photosensitive cells are not organized into a complex organ such as ocelli or lens eyes. Instead, these cells are solitary or grouped and are scattered or localized throughout the animal body. Identification of the cells involved in extraocular photodetection has often proved difficult, but in some animals, neurons, epithelial cells, and muscle cells have been shown to be photosensitive^54^,^56^,^57^,^58^. Intriguingly, an ancient opsin-mediated phototransduction pathway and a previously unknown layer of sensory complexity in the control of cnidocyte discharge in cnidarian *Hydra magnipapillata* was reported very recently^59^. These various extraocular photoreceptors function as light detectors, informing the animal of the presence of light, measuring light intensity, and activating rhythmic behaviors as well as other physiological processes.

Our extensive qRT-PCR analysis (Figs 4, S6, S7) (with support from the phylogenetic data) of different developmental stages and tissues revealed that the *T. cystophora* opsins can be classified into two groups, the probably more ancient Tc-group 1 opsins and Tc-group 2 rhopalium-specific opsins (Fig. 7). Tc-group 1 opsins tend to have broader expression. The broadest tissue- and stage-specific expression distribution is visible in Tc-group 1B, with Tcop2 and Tcop10 being male gonad-specific and other Tcops expressed in tissues such as bell, tentacles or manubrium. Both sub-groups 1A and 1B show a trend for increasing tissue/organ specificity of opsins after subsequent duplications.

More than a half of the opsins from those two subgroups were detected (at least in small amounts) in planula larvae, with Tcop17 (and probably Tcop6) being larva-specific. Such a variety of larval opsins is astonishing considering that to date only three larval opsins have been reported from reef corals^60^. Planula larvae have an extremely simple organization with no nervous system at all. Their only advanced feature is the presence of 10–15 pigment-cup ocelli, evenly spaced across the posterior half of the larval ectoderm. The ocelli are single-cell structures containing a cup of screening pigment filled with presumably photosensory microvilli. These morphologically rhabdomeric-like photoreceptors have no neural connections to any other cells, but each has a well-developed motor-cilium, appearing to be the only means by which light can control the behavior of the larva^61^.

Our analysis implies that Cnidarians extensively utilize opsins not only for visual but also for extraocular photosensitivity. Revisiting the possible diversity of Tcops tissue/stage specific expression by IHC protein expression analysis and physiological studies could shed more light on their use for various behavioral tasks.

### Phototransduction by cubozoan opsins

To investigate the coupling partner of *T. cystophora* opsins we performed an opsin-Gs-cAMP coupling assay. Our data revealed that the Gs-cAMP pathway^27^ is used by opsin genes from Tc-group 2. Moreover our behavioral test showed for the first time that the opsin-Gs-cAMP cascade is functionally connected with vision guided behaviour. However, we were unable to obtain any light-mediated activation of signal transduction via this pathway for Tc-group 1 opsins. We propose that opsins that did not signal in our assay either use different G-protein pathways, as recently proposed in reef corals^60^, act as photoisomerases or for unknown reason do not signal in our cell-line based assay, but nonetheless use Gs signaling cascade under natural conditions. The later possibility is, however, in our opinion very unlikely, because we saw comparable expression of all Tcops on the cell membranes of our test cell system and moreover not even a repeated flash stimulation lead to any response. However, we do acknowledge the possibility, that some of examined Tcops did not fold properly in mammalian cells used in the assay and thus were unable to signal. In some cases we did record slight increases in luciferase signal (like the one in Fig. 2E for Tcop18), however this phenomenon also appeared in some wells containing cells transfected with control opsins (not signaling though Gs-cAMP cascade) or non-transfected cells. This slight increase in luciferase activity was probably connected with non-opsin-specific changes in cellular metabolism during experiment (note that increase in luciferase activity in Fig. 2E starts 140 minutes from the beginning of the experiment and does not seem to be connected with light stimulation). Clearly the future identification of the actual Gα subunit coupled to Tc-group 1 opsins is going to be necessary to understanding if *T. cystophora* possess at least two independent photosystems, thus providing another level for the functional divergence of the identified opsins. Another interesting feature of our assay is the time-course of response of Tcop5, Tcop13 and Caryb transfected cells to light stimulus, reaching the peak in the order of minutes. This phenomenon is probably not caused by the slow light response of the opsins themselves, but rather indicative of the slow kinetics of the recombinant cAMP-sensitive luciferase expression in GloSensor™ cAMP HEK293 cells. In a study by Koyanagi *et al.*^62^, the use of a similar assay led to peak of response in order of minutes even in the case of bovine rhodopsin, which is known to respond to light stimuli in other direct assay systems within millisecond time periods^63^.

Future structure-function studies of prototypical cubozoan group 2 opsin is highly warranted. It would be interesting to find out whether any of the proposed E/D counterions are indeed used by *T. cystophora* opsins. Likewise, the significance of various tripeptide variants found among *T. cystophora* opsins awaits further experimental interrogation. Our data so far point to variable sensitivity and bleaching properties of individual opsins depending on their primary amino acid sequence. Based on their expression and conserved amino acid sequence at key positions, we assume that all Tcops described here are functional opsins, but as mentioned earlier, this remains to be confirmed by other analysis (IHC expression, identification of Tc-group 1 signalling cascade).

In summary, our data suggest that the expansion and diversification of the opsin gene family in cubozoans has allowed fine tuning and optimal photopigment function.

In summary, a detailed expression analysis uncovered both redundancy and specialization in the utilization of the opsin gene repertoire. On the one hand, multiple opsins with presumably similar molecular characteristics are apparently utilized in the same stage/tissue. On the other hand, a clear tendency to establish unique expression patterns exists both within the opsin subfamilies (Tc-group 1 and Tc-group 2) and between the two subfamilies. Remarkably, retina photoreceptors of lens-containing eyes express opsins most probably utilizing at least two distinct signaling pathways.

## Materials and Methods

### Jellyfish collection and culture

Adult *T. cystophora* were collected from the mangroves of La Parguerra, Puerto Rico. Laboratory cultures were established using settling larvae and artificial seawater. Settled larvae metamorphosed into young polyps. Young polyps were transformed into budding (asexually reproducing) polyps by feeding with Artemia once a week. Polyps were stimulated into metamorphosis (transformation into free swimming medusa) by incubation at 28°C. Polyps and young medusa were both maintained at 26 °C. All stages were collected for (opsin) expression pattern analysis (RT-PCR) and juvenile medusa also for rhopalium IHC.

### Isolation of *Tripedalia cystophora* opsin genes

*Tripedalia cystophora* genomic DNA shotgun sequencing was performed on the GS FLX Titanium platform (454 Life Sciences, Roche). Pyrosequencing resulted in 1,952,068 reads (about 7 × 10^8^ bases) with average read length of 360 bp. Assembly generated 134,683 of all contigs containing 790,111 (40.5%) reads. Assembly was done by program Newbler, version 2.3 (Roche). Resulting contigs were combined with singleton reads to produce a complete contig database. The database was subjected to similarity search by the FASTA^64^ program using a wide range of homologous opsin proteins from other cnidarian and bilaterian species. FASTA search provided hits corresponding to short stretches of assumed Tripedalia opsin protein sequences. Full opsin genes sequences were obtained by using the Genome Walking strategy (Genome Walker, Clontech). Opsin sequences were deposited in GeneBank (accession numbers: JQ968416 -JQ968432).

(Primers in Supplement -T1)

### Molecular phylogeny

To investigate the relationship between the cnidarian opsins and bilaterian opsins, we inferred a molecular phylogenetic tree by the maximum likelihood (ML) method implemented in PhyML 3.0^65^ with LG substitution model^66^. Support for internal nodes was assessed using Approximate Likelihood-Ratio Test for Branches^67^.

### Dataset

Opsin protein sequences were acquired as described by Porter *et al.*^3^; however, incomplete sequences were discarded from the analysis and other 26 *Nematostella vectensis* annotated opsins were added to the dataset. In order to root the phylogenetic tree, 22 non-opsin GPCRs from the human genome were used as outgroups. The resulting dataset of 801 (779 opsin plus 22 non-opsin) transcripts plus genome trace opsin sequences was aligned using ClustalX^68^ under default parameters and trimmed by eye in BioEdit. For phylogenetic analyses, only the 7-transmembrane region including intervening inter- and extra-cellular domains was included, as it was difficult to ascertain homology of N- and C- termini due to sequence length variation and lack of conservation across genes. The molecular phylogenetic tree of the opsin family was inferred from an alignment of 226 amino acids long (after N- and C-termini exclusion) opsin sequences. (Sequences in Supplement -T2)

### Quantitative RT-PCR

RNA from indicated stages or dissected adult *T. cystophora* tissues was isolated using TRIZOL reagent (Invitrogen). Contaminating genomic DNA was removed by DNAse digestion and RNA repurification on RNeasy Micro columns (Qiagen) according to the manufacturer’s protocol. The same amounts of RNA from each sample were used for reverse transcription using VILO cDNA kit (Invitrogen). Primers for qPCR were designed using Primer 3 software (see Supplementary Table 1 for sequences of primers). The qPCR was performed in LightCycler 2.0 System using LightCycler® 480 DNA SYBR Green I Master kit (Roche Diagnostics, Germany) according to the standard manufacturer’s protocol. Target genes (Tcop1-Tcop18) and the housekeeping gene (Rpl32) were measured under the same conditions from the same cDNA. Results were analyzed by LightCycler software and crossing point values (Cp) were further determined as an average of Cp values from all replicates and normalized by Cp values of the housekeeping gene (so called deltaCp values). The results show relative normalized gene expression. Statistical significance of changes in the mRNA level of target genes between different samples were calculated by a Student´s t-test. For other data reproduction, heat map from z-scores (Standard scores) of deltaCp values for target genes (Tcop1-Tcop18) expression in different *T. cystophora* tissues was constructed. Z-score representation was obtained in R statistical environment with Bioconductor package.

### Generation and verification of antibodies

An antibody directed against Tcop13 c-opsin was prepared by immunization of mice as follows. The C-terminal region of *c-opsin* corresponding to amino acids 281-330 (*NPIIYCFLHKQFRRAVLRGVCGRIVGGNAIAPSSTGVEPGQTLGGGAAES*; primers in Supplement -T1) was cloned into the expression vector pET42, expressed in BL21(DE3)RIPL cells (Stratagene), and purified by *Ni*-NTA Agarose Beads (QIAGEN). Purified protein was used as antigen for mouse immunization. Human kidney HEK293 cells were transfected with EGFP_C1-c-opsin (amino acids 281–330) expression vector by using FuGENE®6 reagent (Roche). Total extracts were prepared from c-opsin-transfected cells and mock-transfected cells and were analyzed by Western blotting by using anti-c-opsin mouse serum and chemiluminescent detection kit (Pierce).

### Tissue collection and histology

Jellyfish were fixed in 4% paraformaldehyde (PFA), cryoprotected in 30% sucrose overnight at 4°C, and embedded and frozen in OCT (Tissue Freezing Medium, Jung). Horizontal frozen sections were prepared with a 8–12 μm thickness. The cryosections were washed three times in PBS and subsequently immuno-stained with an antibody.

### Immunohistochemistr*y*

The cryosections were refixed in 4% PFA for 10 min, washed three times with PBS, permeabilized with PBT (PBS + 0.1% Tween 20) for 15 min, and blocked in 10% BSA in PBT for 30 min. The primary antibodies were diluted in 1% BSA in PBT (1:500), incubated overnight at 4 °C, washed three times with PBS, and incubated with secondary antibodies in 1% BSA in PBT (1:500). The sections were counterstained with DAPI and mounted. Primary antibodies used were: anti-Tcop18^22^, anti-Tcop13, and anti-acetylated tubulin (Sigma). The following secondary antibodies were used: Alexa Fluor 488- or 594-conjugated goat anti-mouse or anti-rabbit IgG (Molecular Probes).

### Construction of opsin-expressing vectors

The expression vector pcDNA3.1 + 1D4 for opsin gene production in mammalian cells was prepared as follows. The sequence for BamHI restriction site followed by the sequence of 1D4 epitope tag from bovine rhodopsin was introduced into multiple cloning site of pcDNA 3.1+ vector (Clontech) through KpnI and EcoRI sites. Opsin cDNA of box jellyfish *C. rastonii* (GeneBank AB435549), kindly provided Dr. Koyanagi, was amplified from the vector by PCR and cloned into pcDNA3.1 + 1D4 vector using BamHI and HindIII cloning sites. The opsins of box jellyfish *T. cystophora*, which are all intron-less, were amplified by PCR from genomic DNA and cloned into pcDNA 3.1 + 1D4 vector either via BamHI and HindIII or BamHI and KpnI cloning sites. All the constructs were verified by standard sequencing techniques before use.

### Immunofluorescent staining of GloSensor™ cAMP HEK293 cells

GloSensor™ cAMP HEK293 cells (Promega) (2.5*10^3^) were seeded onto coverslips and transfected with FuGene HD (ROCHE). The next day, cells were washed with PBS and fixed with 4% paraformaldehyde (PFA) for 10 minutes. Fixed cells were permeabilized with 0.1% Triton X-100 for 10 minutes and blocked with 10% BSA in 1x PBS with 0.1% Tween 20 for 1 hour. A mouse monoclonal antibody raised against 1D4 epitope (Millipore Chemicon MAB5356), at a concentration of 1:250, was used in conjunction with a secondary antibody conjugated with Alexa Fluor 488 to immuno-stain expressed opsins. Cells were mounted in Mowiol^®^. Fluorescent images were captured using a Leica SP5 confocal microscope.

### Light response assays

GloSensor™ cAMP HEK293 cells (Promega) (10*10^3^) were plated into a solid white 96-well plate in L15 CO_2_-independent medium with phenol red (Gibco) and 10% serum and incubated overnight at 37 °C, 0.3% CO_2_. The cells were transfected the next day with plasmids expressing opsin genes using FuGENE® HD Transfection Reagent (ROCHE). Immuno-fluorescent staining revealed a transfection efficiency of 50% using this method. All procedures following transfection of the cells with the various opsin receptors were carried out in dim red light. Six hours post transfection 9-cis retinal (Sigma-Aldrich) was added to a final concentration of 10 mM. The cells were then kept overnight in an incubator (37 °C; 0.3% CO_2_). Next day the cells were removed from the incubator and left to equilibrate for 30 minutes at room temperature. Beetle luciferin potassium salt (Synchem) reconstituted in 10 mM HEPES buffer was added to the cells to a final concentration of 3 mM. The cells were then placed in a top-read Envision plate reader with ultra-sensitive luminescence model. Luciferase activity was measured for 2 hours with 0.1 second resolution and cycles of every 1 minute to determine the luciferin uptake. Cells were then subjected to three pulses of light stimulation using repeated flashes from a Nikon speedlight SB-600 electronic camera flash (5 flashes, 1 flash/ second in each pulse, ~40000 lumen/m2 per flash) followed by recovery periods of 30 minutes when Raw Luminescence Units (RLU) were recorded. After the third measurement, the cells were stimulated with seven light pulses with periods of 3 minutes (5 flashes, 1 flash/ second in each pulse). Luminescence was recorded between pulses (0.1 second resolution, 15 seconds per cycle) and another 120 minutes after the last pulse (0.1 seconds resolution, 30 seconds per cycle). The experiment for the tripeptide mutation was performed in a similar way with minor changes. The entire experiment was performed at 37 °C, which led to faster response of cells to the light stimulation. Three pulses (5 flashes, 1 flash/ second in each pulse) followed by recovery of 15 minutes were applied. The following repeated stimulation was done with 30 light pulses (1 flash) with periods of 30 seconds. Luminescence was measured another 30 minutes after the last pulse. Luminescence recordings were analyzed with Microsoft Office Excel. All experiments comprised cells plated and treated in triplicate. Prism (Graphpad) software was used for all statistical analyses.

### *T. cystophora* phototaxis test

All behavioral tests were performed at room temperature (22 °C). Phototaxis experiments were performed in an aquarium-like testing chamber (20 × 5 × 5 cm) with one illuminated side (Fig. 7a). To test the effect of suramin analog 4,4‘,4“,4“‘-(carbonylbis(imino-5,1,3-benzenetriylbis (carbonylimino)))tetrakis-benzene-1,3-disulfonic acid (NF449 - Calbiochem) on the *T. cystophora* phototactic behavior, we incubated 3-day-old medusae in 1 ml of artificial seawater with NF449 at a final concentration of either 0 μM, 100 μM, and 1 mM for 30 minutes under artificial day light. Medusae were then washed with artificial seawater, placed into the dark part of the testing chamber and tested for phototactic behavior. The number of medusae that reached the light region after 5 minutes, 3 hours and 24 hours was counted and compared to the number of animals from the untreated control group.

## Additional Information

**How to cite this article**: Liegertová, M. *et al.* Cubozoan genome illuminates functional diversification of opsins and photoreceptor evolution. *Sci. Rep.* **5**, 11885; doi: 10.1038/srep11885 (2015).

## Supplementary Material

Supplementary Video

Supplementary Information
