# Corrigendum: Cubozoan genome illuminates functional diversification of opsins and photoreceptor evolution

**DOI:** 10.1038/srep14396

**Published:** 2015-09-24

**Authors:** Michaela Liegertová, Jiří Pergner, Iryna Kozmiková, Peter Fabian, Antonio R. Pombinho, Hynek Strnad, Jan Pačes, Čestmír Vlček, Petr Bartůněk, Zbyněk Kozmik

Scientific Reports 5: Article number: 1188510.1038/srep11885; published online: 07082015; updated: 09242015.

This Article contains a typographical error in the grant number in the Acknowledgements section.

“This study was supported by the Grant Agency of the Czech Republic (P305/10/2141) to Z.K. by the Ministry of Education, Youth and Sports of the Czech Republic (LM2011022) to P.B. and by IMG institutional support RVO68378050”.

should read:

“This study was supported by the Grant Agency of the Czech Republic (P305/10/2141) to Z.K. by the Ministry of Education, Youth and Sports of the Czech Republic (LO1220) to P.B. and by IMG institutional support RVO68378050”.

