# Supplementary material for: Cubozoan genome illuminates functional diversification of opsins and photoreceptor evolution
Source: Sci Rep. 2015 Jul 8;5:11885. doi: 10.1038/srep11885 (PMC5155618; doi:10.1038/srep11885)
Supplement: Supplementary Information [file srep11885-s2.pdf]

Supplementary information:

Cubozoan genome illuminates functional diversification of opsins and photoreceptor evolution

Michaela Liegertová[1a], Jiří Pergner[1a], Iryna Kozmiková[1], Peter Fabian[1], Antonio R. Pombinho[2], Hynek Strnad[3], Jan Pačes[3], Čestmír Vlček[3], Petr Bartůněk[2] and Zbyněk Kozmik[1*]

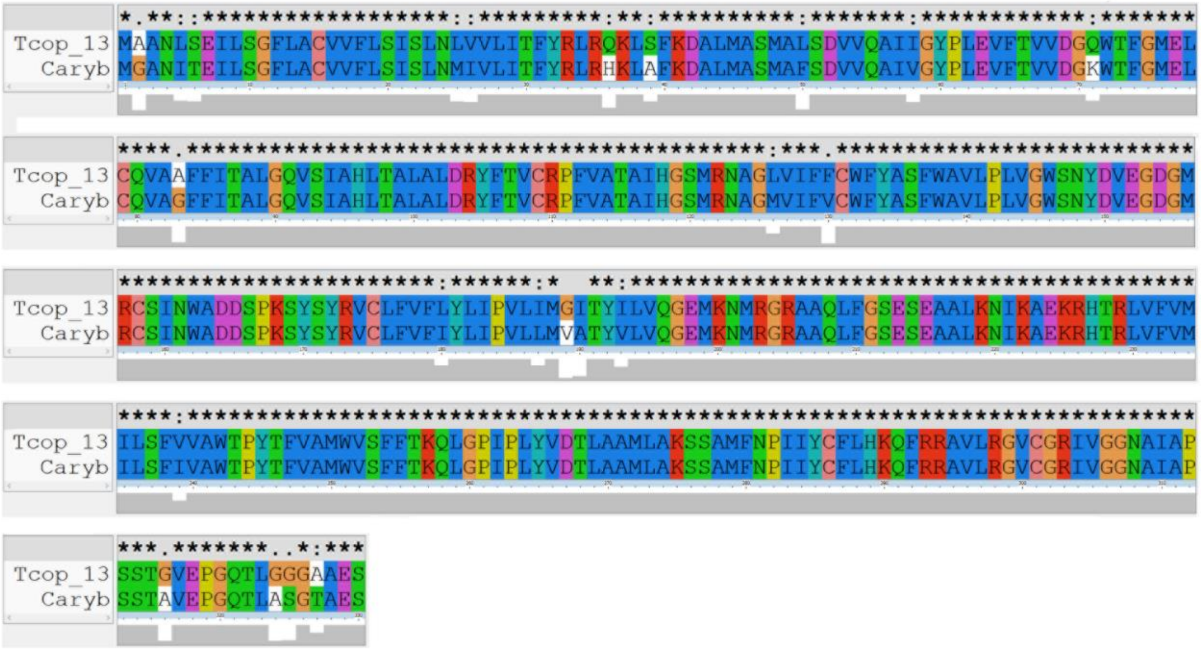

Figure S1 - Pairwise sequence alignment of Tcop13 and *Carybdea rastonii* opsin

Alignment showing homology between opsins, Tcop13 and Caryb, from two cubozoan species *Tripedalia cystophora* and *Carybdea rastonii*. Calculated sequence identity 93 %.

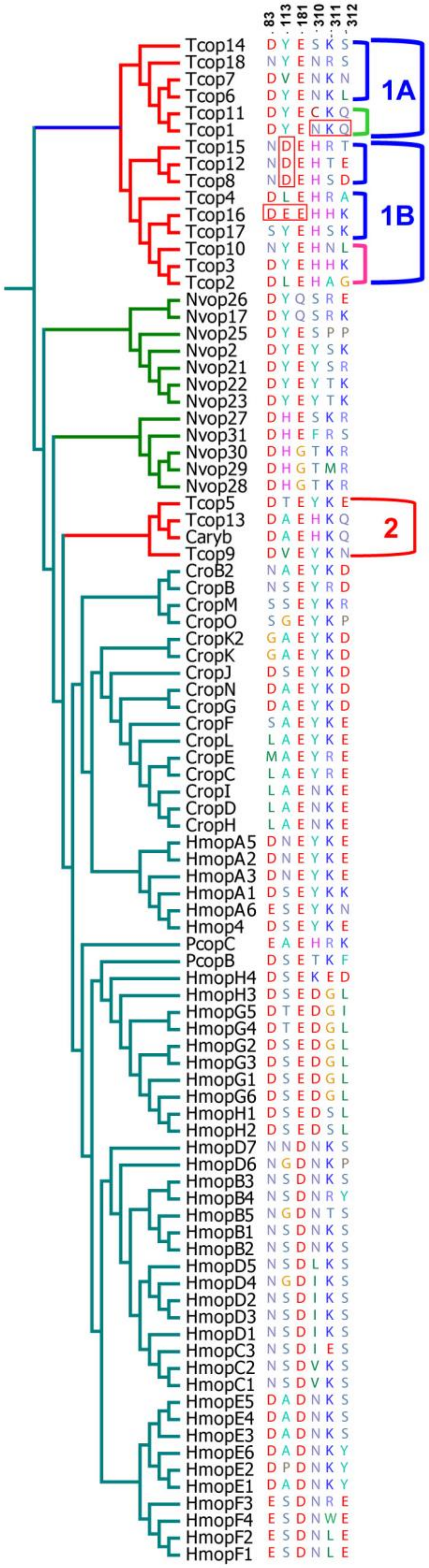

Figure S2 – Comparison of counterion and tripeptide diversity within cnidopsins

Partial sequence alignment of amino acid residues at three potential counterion sites (bovine rhodopsin sites 83, 113, 181) and the G protein-binding tripeptide (bovine rhodopsin sites 310, 311, 312) within the cnidopsin branch. Interesting amino acid residues are highlighted by red box. Tcop – *Tripedalia cystophora* (Cubozoa); Nvop – *Nematostella vectensis* (Anthozoa); Crop – *Cladonema radiatum* (Hydrozoa); Hmop – *Hydra magnipapillata* (Hydrozoa); Caryb – *Carybdea rastonii* (Cubozoa).

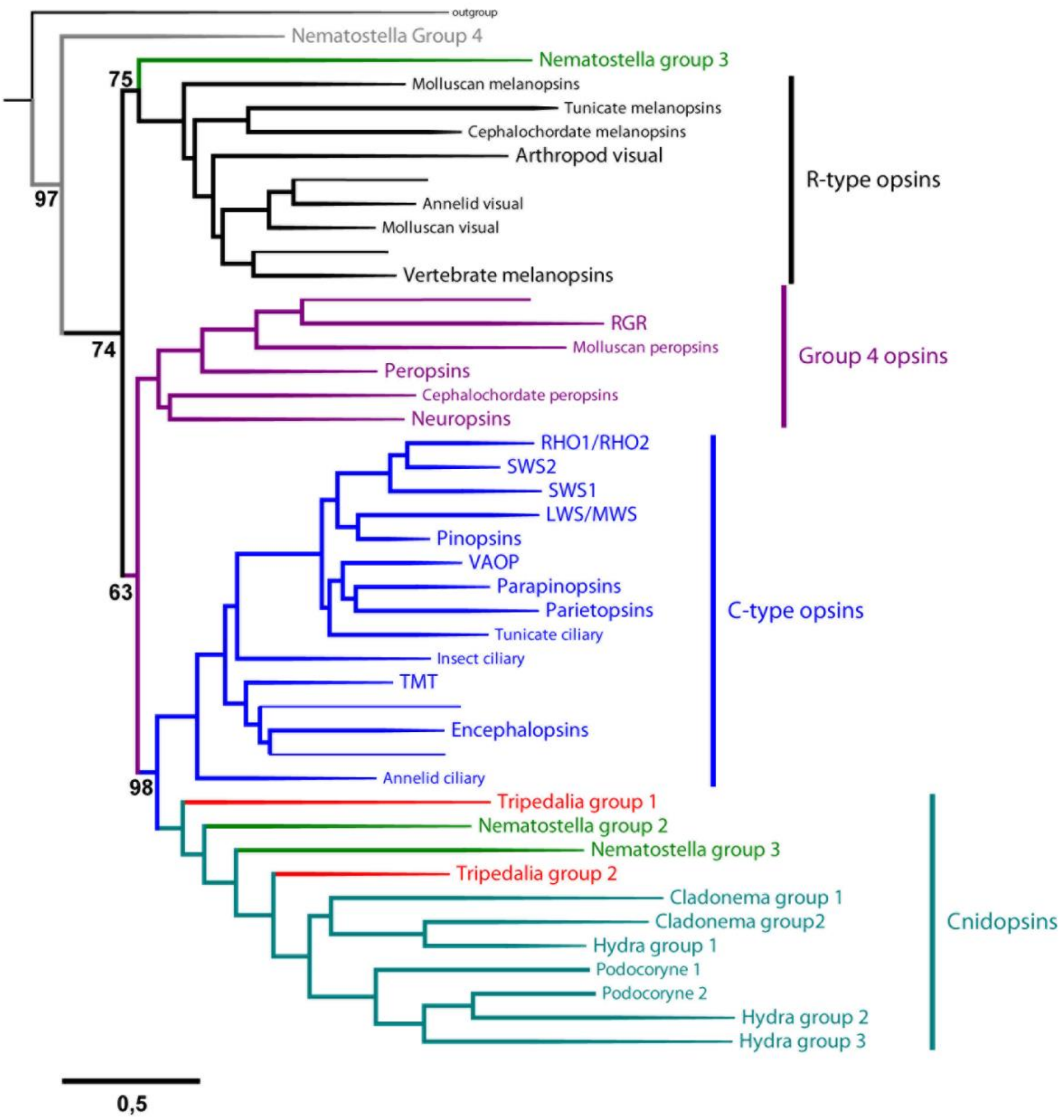

Figure S3 – Maximum-likelihood tree of the opsin family

The maximum-likelihood tree of 801 protein sequences with the branches collapsed into well-supported clades where possible. Approximate Likelihood-Ratio Test (aLRT) branch support values (%) are shown for major groups. The four major opsin clades have been labeled. R-type opsin group: arthropod visual pigments (M/LWS, SWS); annelid and molluscan visual pigments; vertebrate

melanopsins; uncharacterized tunicate, cephalochordate and molluscan opsins. Group 4 opsins: neuropsins; peropsins; RGR and uncharacterized cephalochordate and molluscan peropsins. C-type opsin group: vertebrate visual pigments (Rh1, Rh2, SWS1, SWS2, M/LWS); pinopsins; parapinopsins; vertebrate ancient opsins (VAOP); parietal opsins; teleost multiple tissue opsins (TMTs); encephalopsins; tunicate ciliary opsins; ptersopsins and insect ciliary opsins; uncharacterized annelid ciliary opsins. Cnidopsins: cnidiarian opsins including representatives from hydrozoans, anthozoans and cubozoans. Nematostella group 4 and 1 are novel cnidarian opsin subfamilies, which do not cluster within the cnidopsin group.

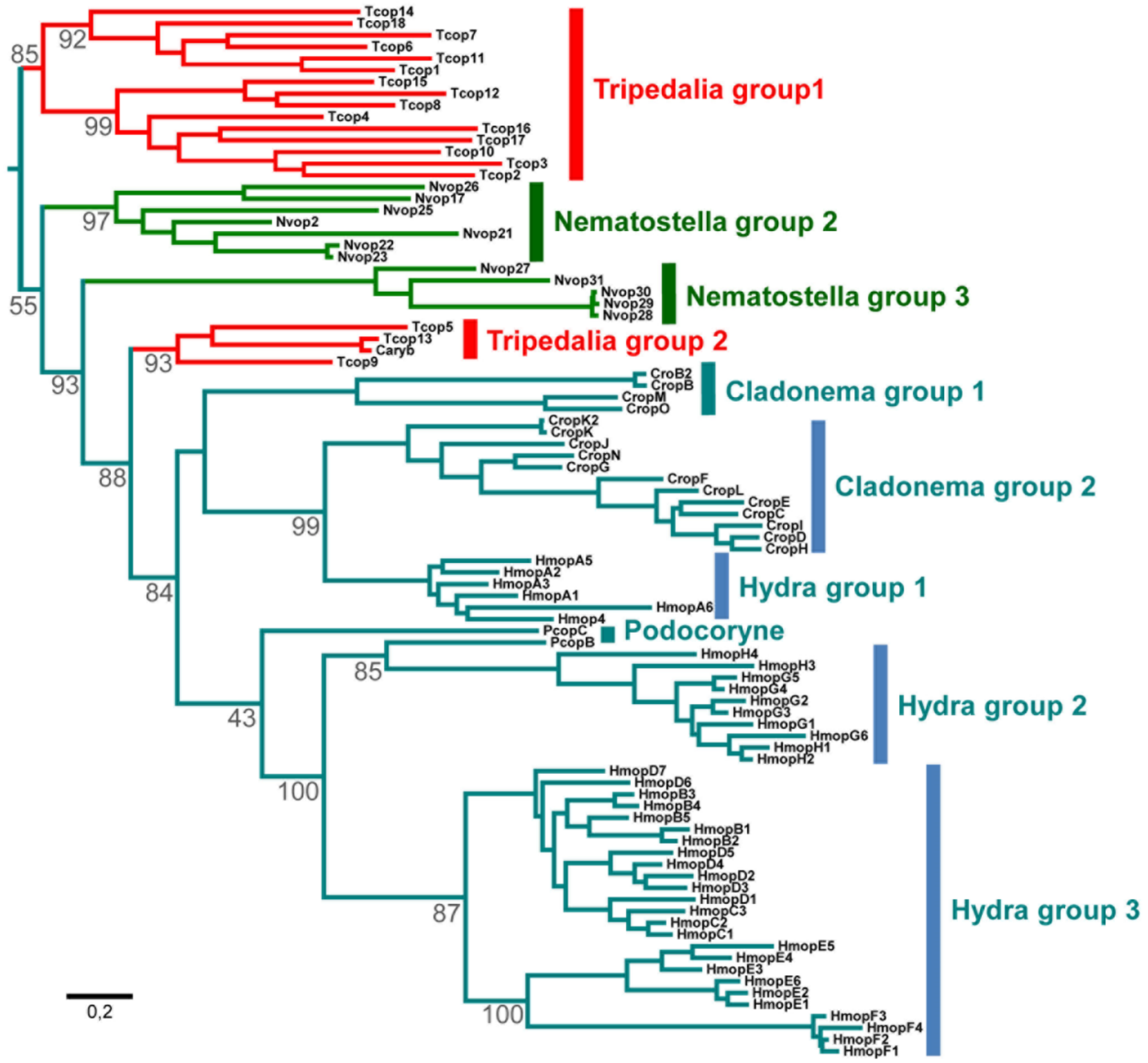

Figure S4 – Zoom into the cnidopsin branch of the opsin family maximum-likelihood tree

The major cnidopsin clades have been labeled. Approximate Likelihood-Ratio Test (aLRT) branch support values (%) are shown for major groups. Tcop – *Tripedalia cystophora* (Cubozoa); Nvop – *Nematostella vectensis* (Anthozoa); Crop – *Cladonema radiatum* (Hydrozoa); Hmop – *Hydra magnipapillata* (Hydrozoa); Caryb – *Carybdea rastonii*.

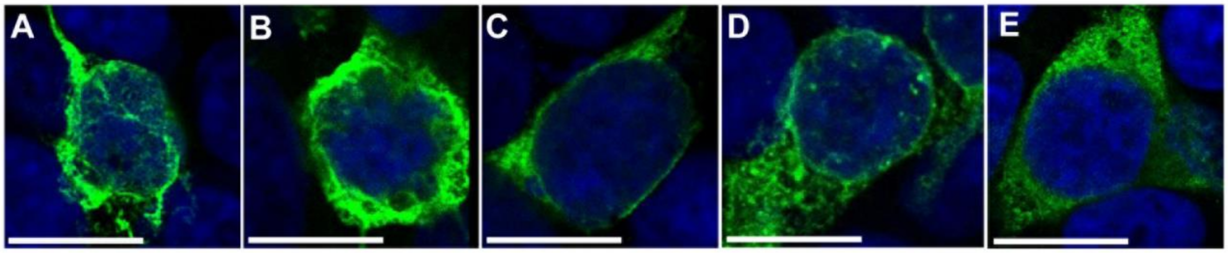

Figure S5 – **Expression of recombinant *T. cystophora* opsins in GloSensor™ cAMP HEK293 cells**

A-D) Opsins tagged with 1D4 tag were expressed in GloSensor™ cAMP HEK293 cells (Promega) and stained as described in Materials and Methods (blue – DAPI and green – 1D4 tag). Opsin signals were detected on the cell membrane. **A)** Tcop5. **B)** Tcop13. **C)** Tcop18. **D)** Tcop9. **E)** *Oryzias latipes* rhodopsin used as control. (Scale bars: 10 μm)

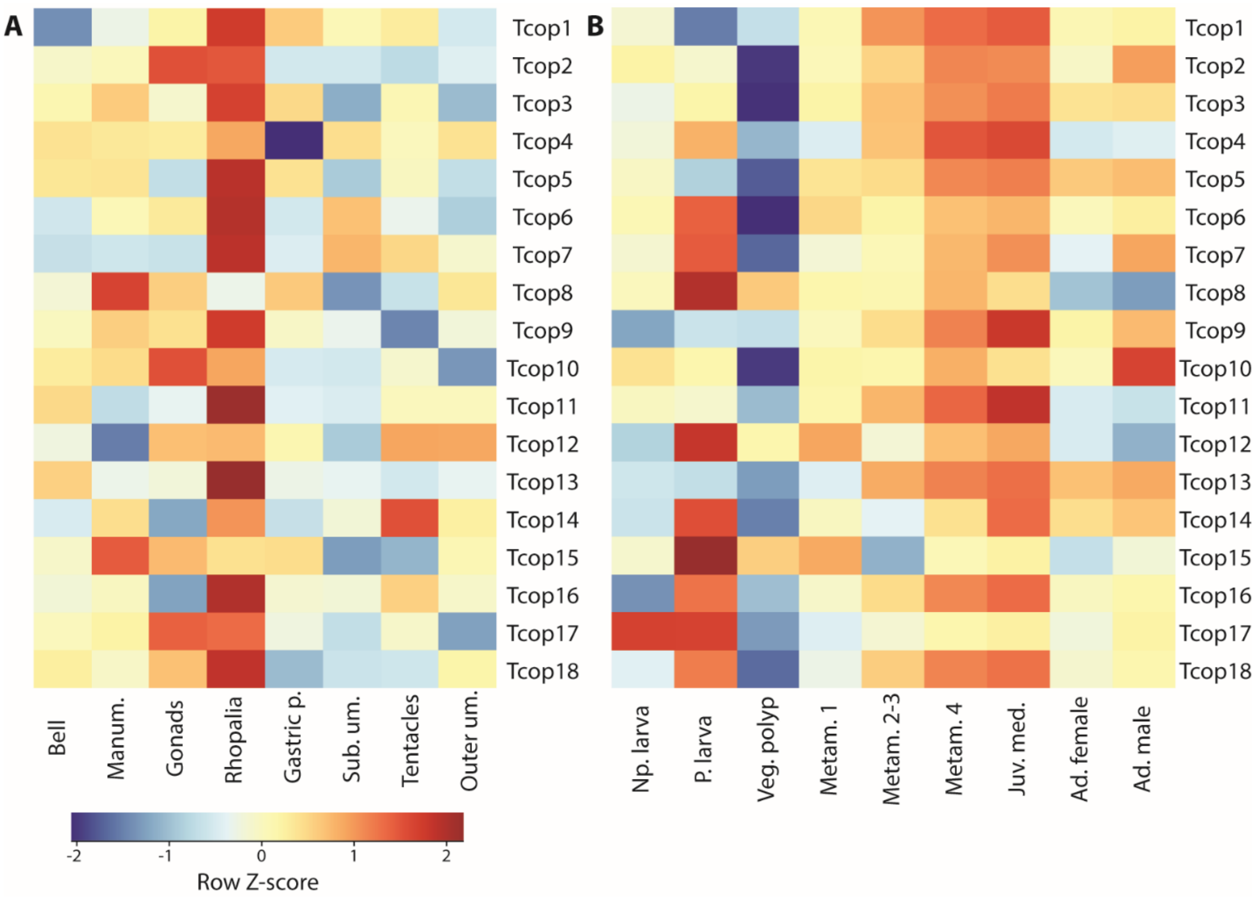

Figure S6 – mRNA expression levels of individual *T. cystophora* opsins in dissected body parts of adult jellyfish and life stages displayed as Z-score heat map

Opsin expression was normalized to expression of housekeeping gene Rpl32 and plotted as Z-score heat map (as described in Materials and methods). A – qRT-PCR results of Tcops expression in disected *Tripedalia* body parts. Schematic representation of dissected body parts can be found in Fig. 4. Manum. – manumbrium, Gastric p. - gastric pouch, bell, Sub. Um. – sub-umbrella, Outer um. - outer umbrella**.** B – qRT-PCR results of Tcops expression during *Tripedalia* life cycle. Schematic representation of life stages can be found in Fig. 5. Np. Larva - non-pigmented larva, P. larva - pigmented larva, Veg. polyp - vegetative polyp, Metam. 1, Metam. 2-3, Metam. 4 - three polyp-to-medusa metamorphosing stages, Juv. med. - juvenile medusa, Ad. female - adult female and Ad. male - adult male.

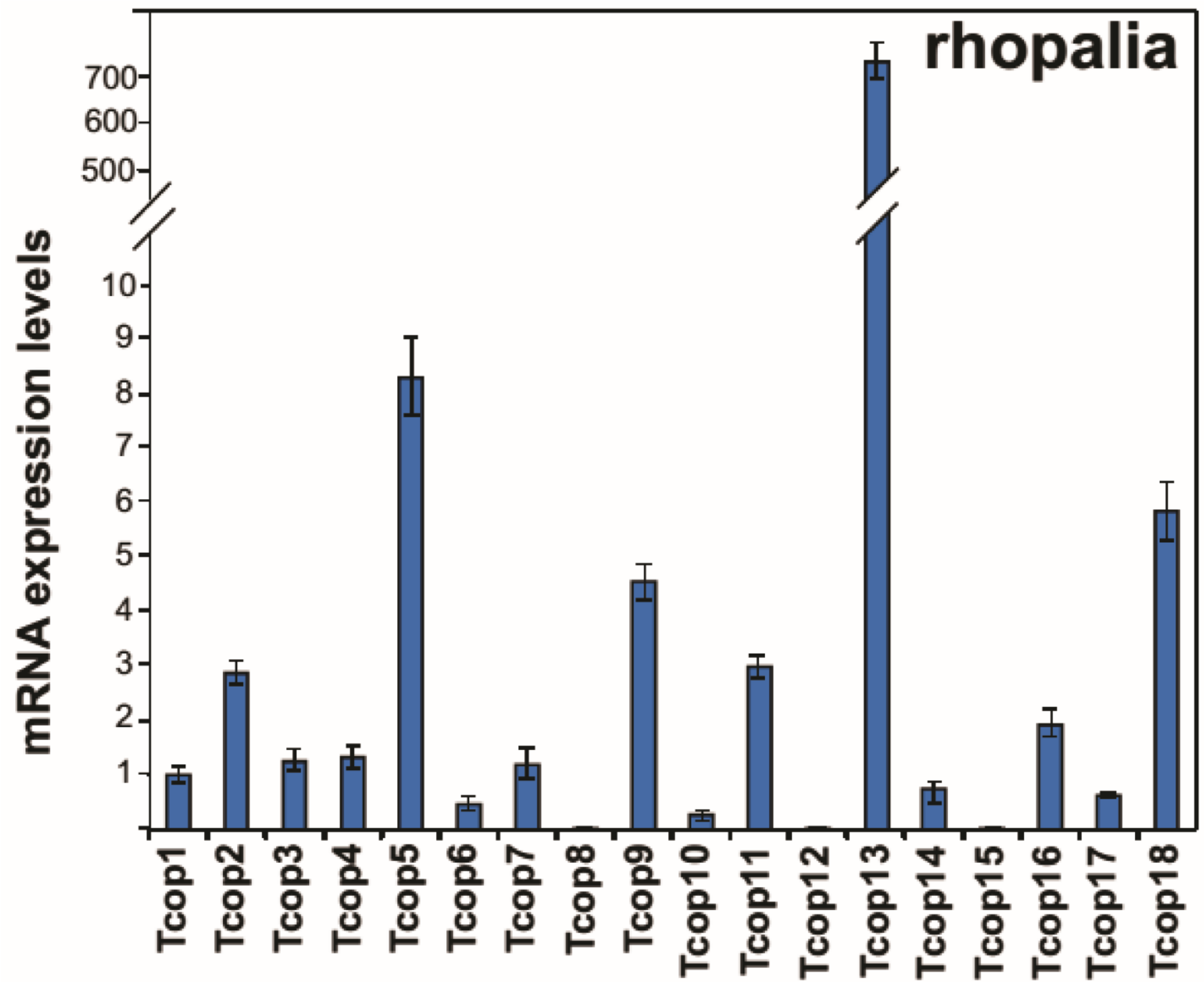

Figure S7 – mRNA expression levels of opsins in rhopalium and gonads

Comparison of all *T. cystophora* opsin mRNA levels, measured by real-time PCR in rhopalia.

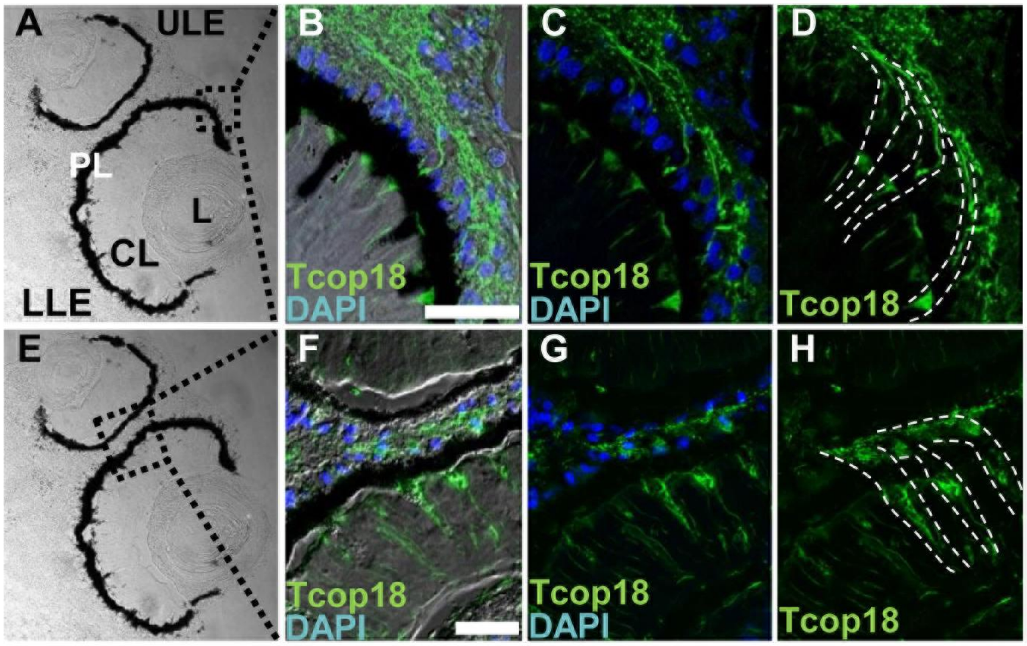

Figure S8 – Details of type-A photoreceptor cells in lens eye retinas

A-H) Confocal images of immunohistochemical staining for Tcop18 (green) in the examined parts of lens eye retinas. **D, H)** Shapes of type-A photoreceptor cells are indicated with a dotted white line.

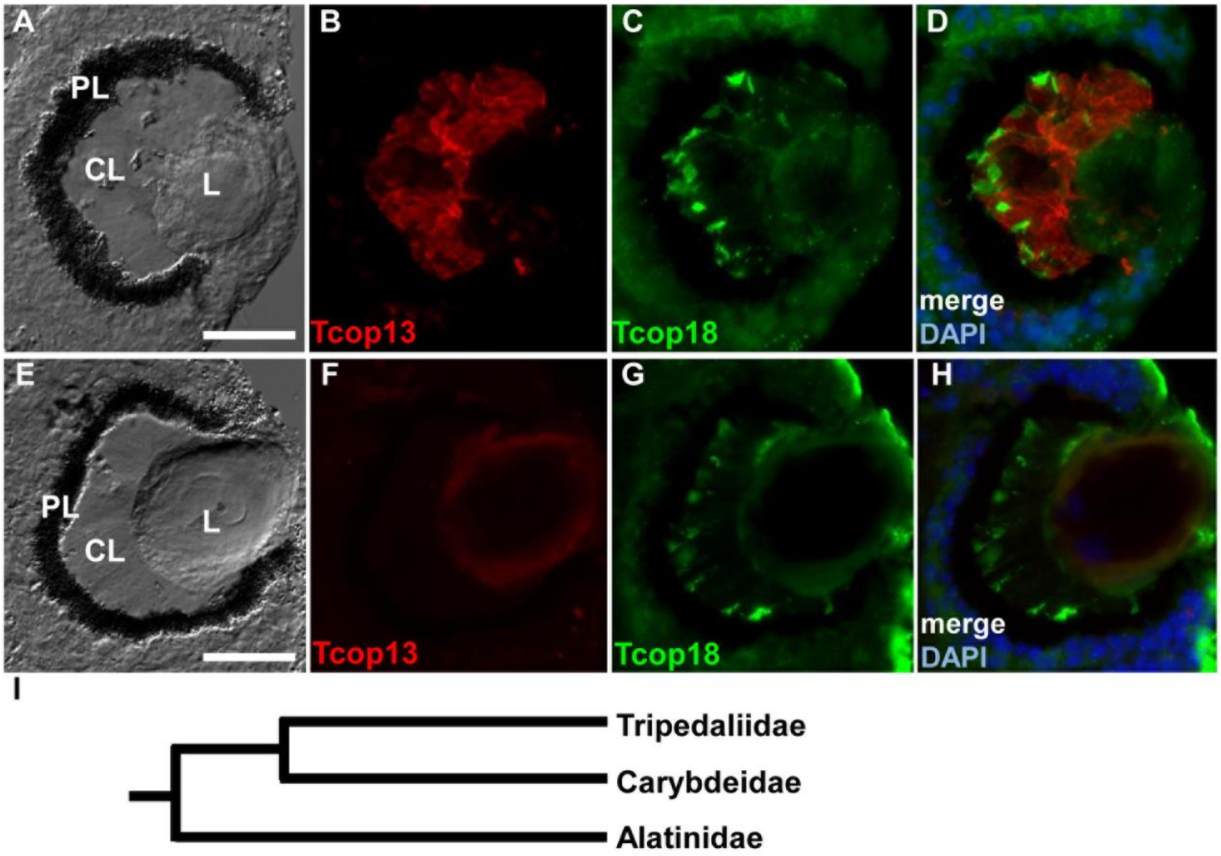

Figure S9 – Comparison of Tcop13 and Tcop18 expression in the retinas of lens eye between Tripedaliidae and Alatinidae

A-D) Confocal images of immunohistochemical staining for Tcop13 (red), Tcop18 (green) and DAPI (blue) in the developing LLE of juvenile *T. cystophora* medusa. **E-H)** Developing LLE of juvenile *Alatina marsupialis* medusa. **F)** Absence of Tcop13 signal in the retina. **I)** Phylogenetic relationship between Carybdeid families. (Scale bars: 20 µm)

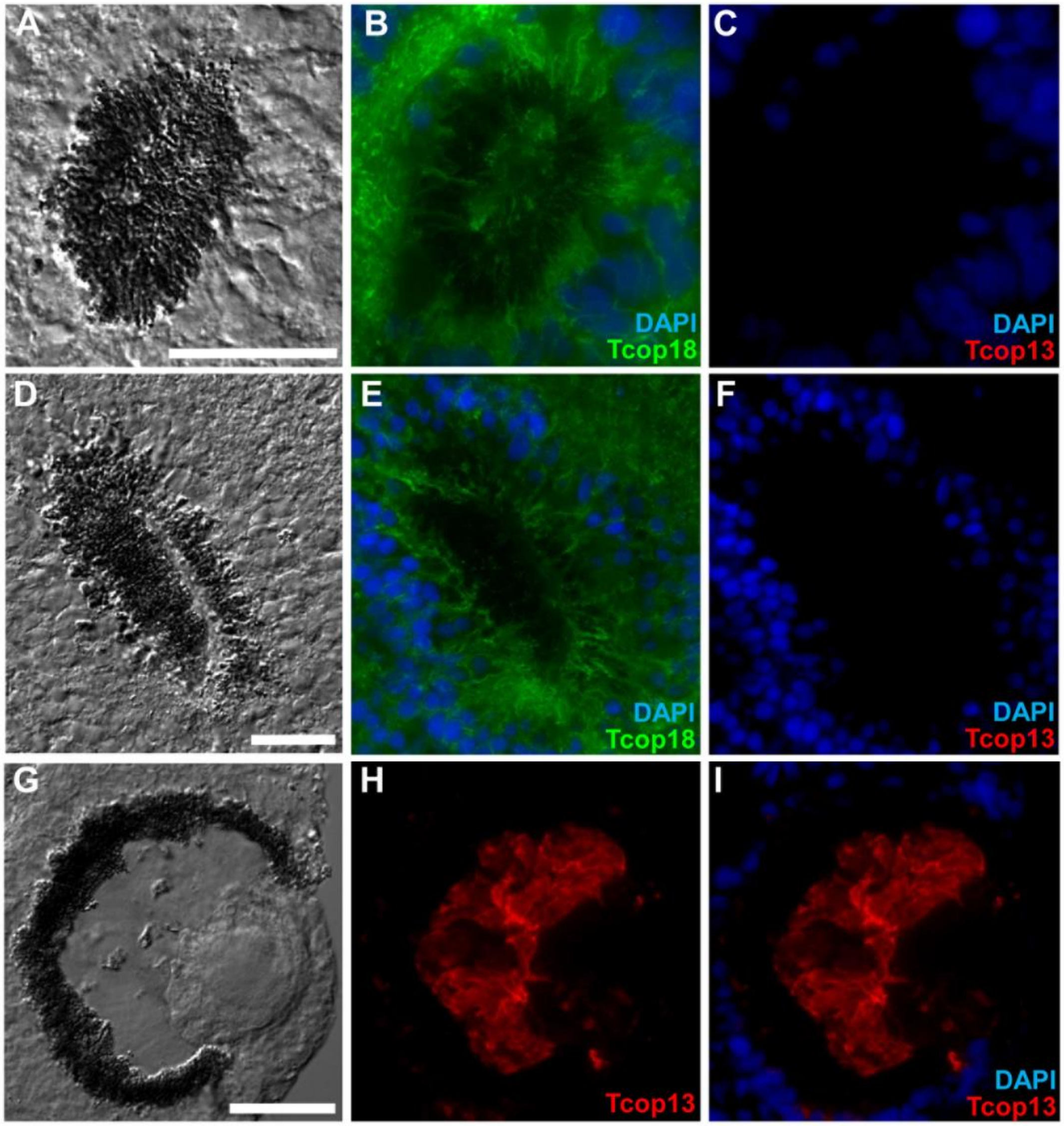

Figure S10 – Expression pattern of Tcop18 in the pit and slit ocelli of *T. cystophora*

A-D) Confocal images of pit and slit eyes. **A)** Pit eye as seen in bright field. **B)** Immunohistochemical staining for Tcop18 (green) and DAPI (blue). **C)** Staining for Tcop13 (green) and DAPI (blue). Signal for Tcop13 was not present in the retina. **D)** Slit eye as seen in bright field. **E)** Immunohistochemical staining for Tcop18 (green) and DAPI (blue). **F)** Staining for Tcop13 (green) and DAPI (blue). Signal for Tcop13 was not present in the retina. Both ocelli types seem to be formed exclusively of type-A photoreceptors. (Scale bars: 20 μm) **G)** LLE as seen in brightfield. **H)-I)** Staining for Tcop13 (red) and DAPI (blue); used as positive control. (Scale bars: 20 μm)

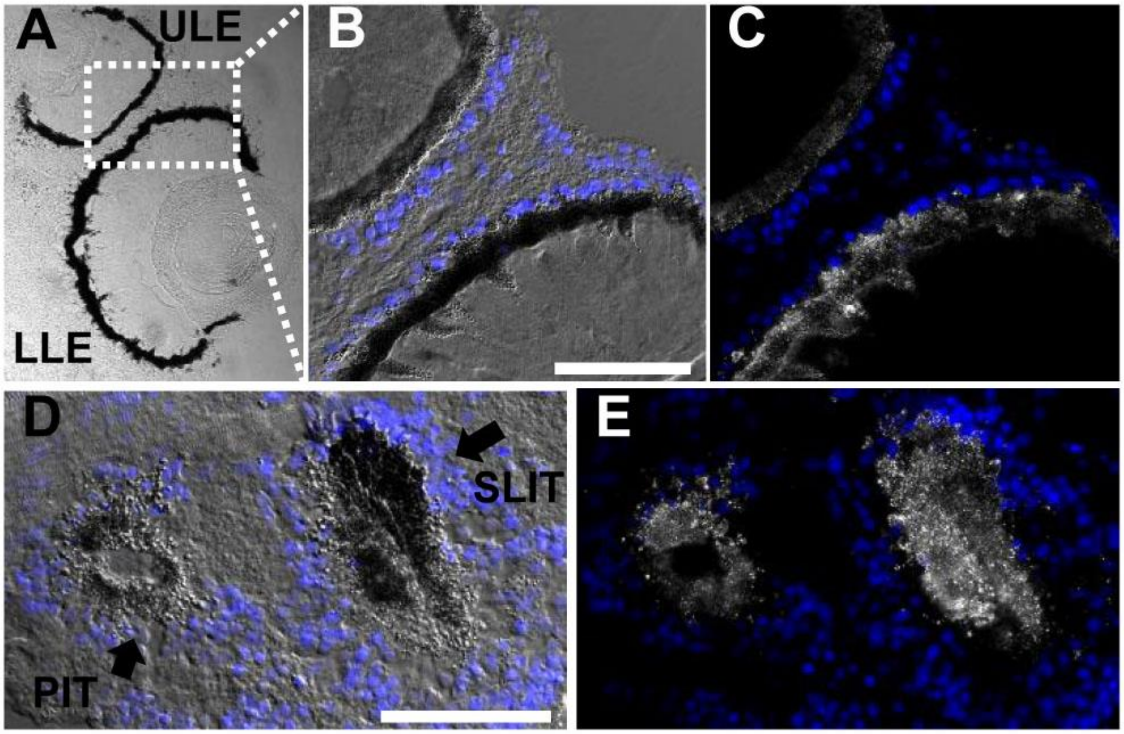

Figure S11 – Types of shading pigment granules in the photoreceptors

A-C) Detail of the pigmented areas of ULE and LLE retinas**. A-B)** In bright field, both types of pigment granules appear dark. **C)** The same detail in polarized light. Part of the granules alter the plane of polarization and appear white**. D-E)** Both types of pigment granules are present in the retinas of pit and slit eyes. **D)** Pit and slit eyes as seen in bright field. **E)** Pit and slit eyes as seen in polarized light. DAPI (blue). (Scale bars: 50 μm)

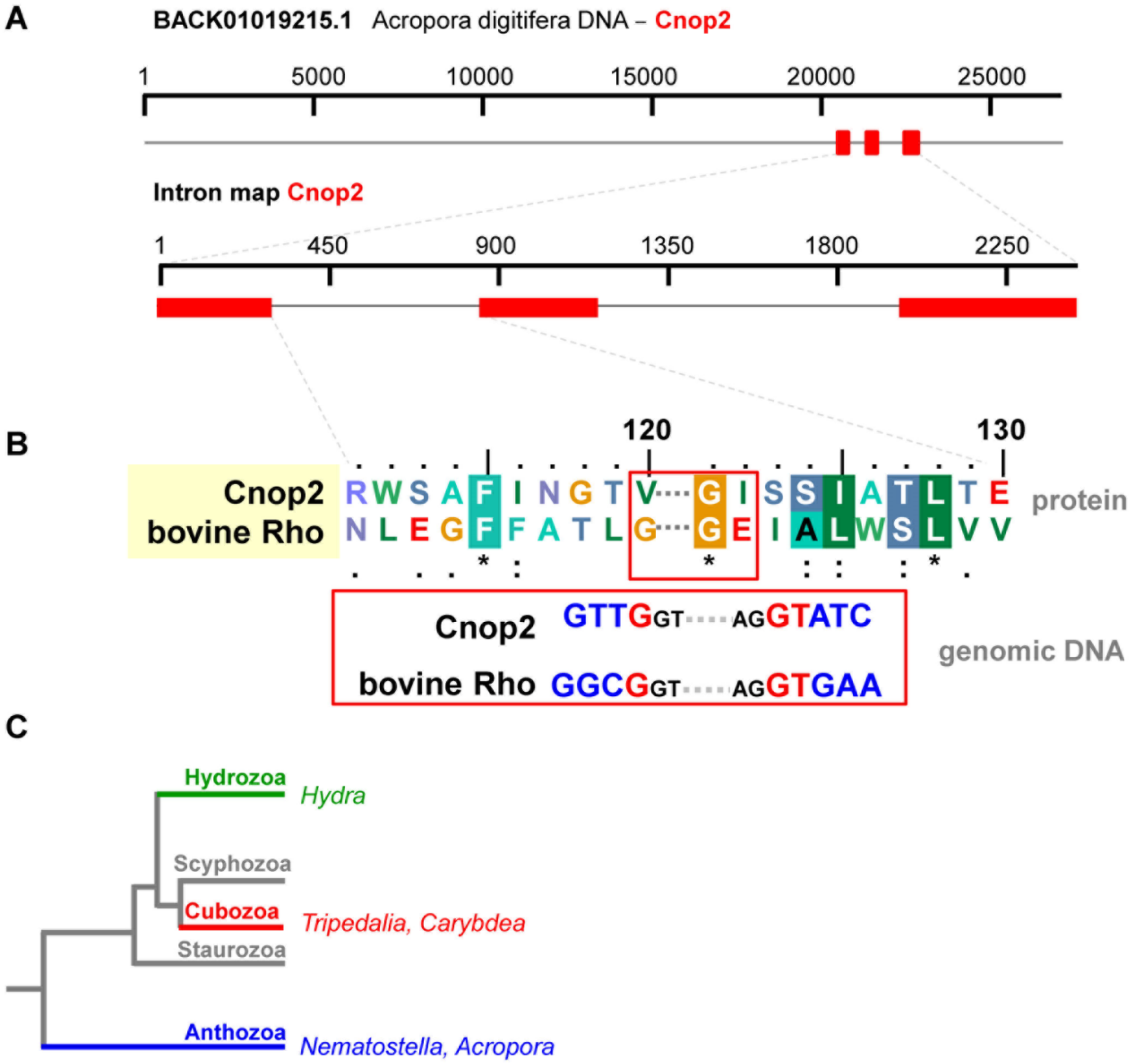

Figure S12 –Intron characterization of Cnop2 opsin from anthozoan *Acropora digitifera*

A) Cnop2 genomic localization and intron map. **B)** Partial protein alignment of bovine rhodopsin and Cnop2. Positions of the first intron in both genes are highlighted by red line. Introns from both species match in position and in phase (as shown on the genomic DNA). **C)** Phylogenetic relations within cnidaria.

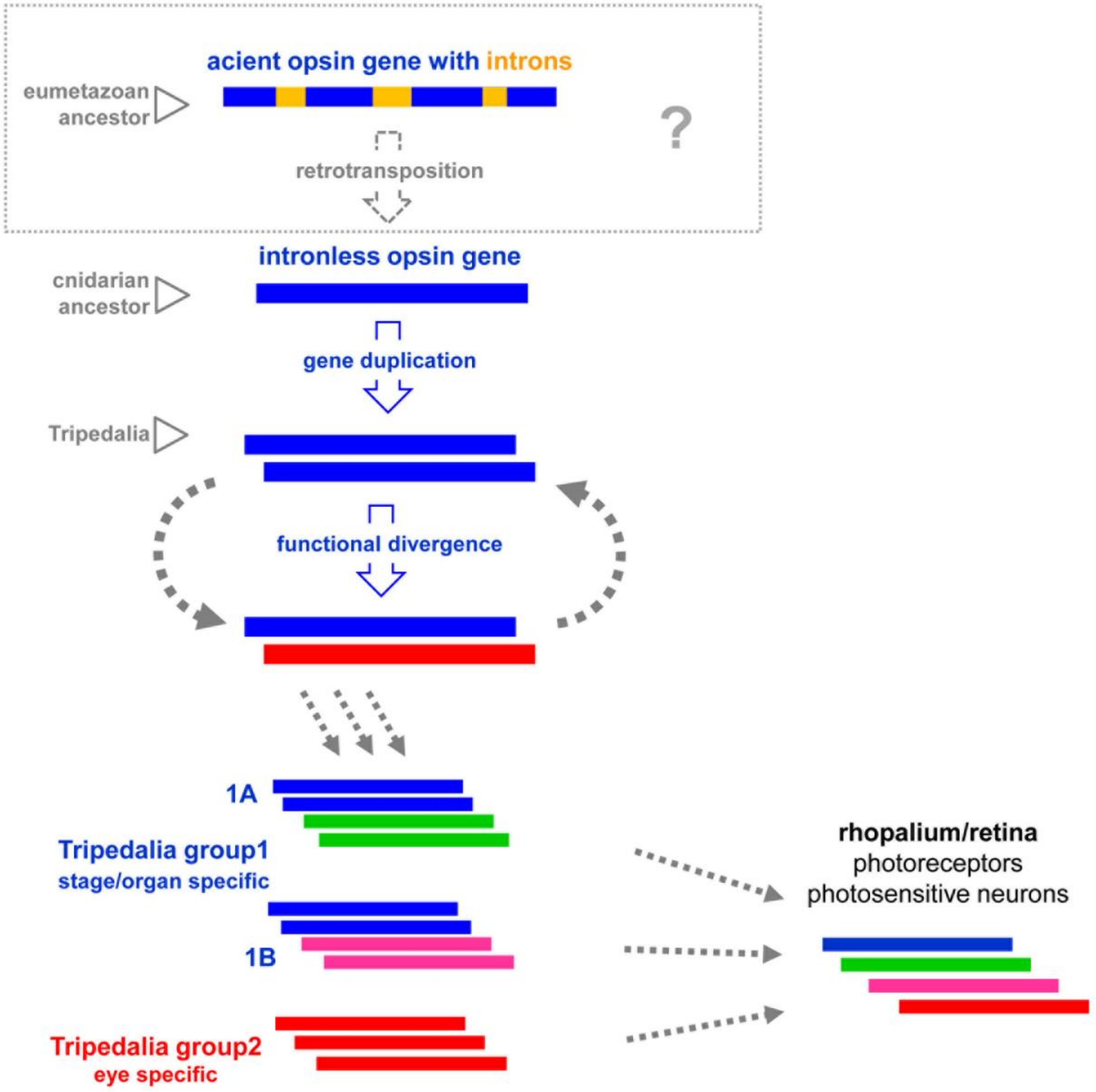

Figure S13 - Possible scenario for intron-less opsin emergence and subsequent expansion in *T. cystophora*

For further information see discussion.

Additional file – *T. cystophora* phototaxis test movie

Movie showing response of living *T.cystophora* medusa to light stimulus without and with presence of Gs cascade specific inhibitor. MPG movie can be viewed in any video software.

Gene	reverse primers for 5´end direction walking
Tcop1	CCATTACCATCAGGAAGACCAACAAGG
Tcop2	TCGATTATCTCACGTGATACCCACTTGG
Tcop3	GGTGCACAACCTACATGTTGACTTTCAG
Tcop4	GGACATACTATTCCAATCACCTGCTTTCC
Tcop5	CCAAAGTGACTCTTGGAGCGATGTC
Tcop6	CCAACGACGATCTGGATTGCCAATTAATG
Tcop7	CACCTGATTTCTAGTAGGATGCAGGCA
Tcop8	CTAGGGCTGCATTGGATCCAAACTTTG
Tcop9	AGGTCGTCCATAAAGAAAGAATGGCAT
Tcop10	CAGATGCGAAGTTTGTCCTGGATATCA
Tcop11	AAGCTGCTTGGAGGCTGATGAGATT
Tcop12	ACTGACTAAATTGCTGTCAATGGTCACG
Tcop13	ATGGCGGCTAACTTGTCTGAAATACTTTC
Tcop14	CCCTCTGTAGAATAACTTGAGAGTCCAGTA
Tcop15	CTACATTGCCTGTGACGCTCTCATG
Tcop16	GCCTCTTTATTTCAGCTCTAAACTTATGATGC
Tcop17	CGGATATTTACATGACGTCGCATCATCC

Gene	forward primers for 3´end direction walking
Tcop1	CACAAAGTGCAAGAGAAATCGAGAGACA
Tcop2	CCCAGTGCATACTGTCTCTTATATACTCG
Tcop3	ACCTACAAGAGTTCTCTCCTATCTGAAGG
Tcop4	CGAACCAGAACAGCTGTCGTAATCTT
Tcop5	TCATGTTCTACATCAGAAGCGAATTGACG
Tcop6	TGCTTACCCACTCTCAGCATATTCCA
Tcop7	CAACTTCTAAGTAACAACAGGACTCGGT
Tcop8	CCTCTCAACATTGTATCATGCTTTCAGC
Tcop9	GCACTCGACCGTTATATGACAGTGT
Tcop10	GGGATCAGCAGAGATTAAGGATGCTACT
Tcop11	TACGATGGCACAGTGATGACAGCA
Tcop12	ATCCTCGGTCTGCTATCTACCTGTTG
Tcop13	GCAGTGGACATTTGGTATGGAGCTA
Tcop14	CGCTATCAATATCGTCATCTTTTGCCAC
Tcop17	AGTTATTGCCTATCGCCAGTGGAAG

	anti-Tcop13 antibody preparation	
Tcop13	CGCGGATCCAACCCGATCATTTACTGCTTCCTT	forward
	CCCAAGCTTTTAACTCTCTGCAGCCCCTC	reverse

Gene/Product size	RT-qPCR primers:		
Tcop1/212 bp	1231A	TATGCCCGTTGTTTGCCTATC	forward
	1231B	GGTGACATTTTGGCGATTGAT	reverse
Tcop2/119 bp	1232A	AGTTGCTGCCCAGATTGTGTT	forward
	1232B	TTGTAGTGGGCGAGTTCCTGT	reverse
Tcop3/217 bp	1233A	GACCCGCTCGATGTGTCTTAC	forward
	1233B	AGGGAAGCCAACAGAACACAA	reverse
Tcop4/228 bp	1234A	CAGTCTCTTCGGTGGCTCACT	forward
	1234B	TCCAATCACCTGCTTTCCAGT	reverse
Tcop5/205 bp	1235A	TTGACGCGCTCTACTTTGTCA	forward
	1235B	GACATAAGCATCACGGCAACA	reverse
Tcop6/170 bp	1236A	GCACGGGTCAAACTCTCAAAC	forward
	1236B	ATTCTGCTATTGGGCTCACGA	reverse
Tcop7/155 bp	1237A	TTGCTGGTCTTCCTCATTGGT	forward
	1237B	GGAAGCAGCAAACAAGAATGG	reverse
Tcop8/194 bp	1238A	TGCATATTAGGCGTCACAACG	forward
	1238B	AATTGGGTTTAATGGGCCTTG	reverse
Tcop9/150 bp	1239A	ACCCAAGAAAAACCGAAAGGA	forward
	1239B	CAGAGCAACGCACTTTCACAC	reverse
Tcop10/227 bp	1240A	CTTGGGCATCTGTTGATGGTT	forward
	1240B	CAGCCATCGTAGCCAAATCTC	reverse
Tcop11/211 bp	1241A	ACCTGGCTATTCGGAGATGGT	forward
	1241B	GTAGTGCTGCCCAGACAAACC	reverse
Tcop12/160 bp	1242A	GCAACCTCTTGCATGTCACAA	forward
	1242B	AAGCGATCAACTGCCAACAAT	reverse
Tcop13/224 bp	1243A	CTGCACAGCTGTTTGGTTCTG	forward
	1243B	GAACATGGCTGAGGACTTTGC	reverse
Tcop14/178 bp	1244A	GACTGGTCTTGCCTTTGATCG	forward
	1244B	GCTCACCTGGAGACCCTCTTT	reverse
Tcop15/238 bp	1245A	GGCTGAAAATGCGAGAGAAGA	forward
	1245B	ACCATAACCCCAATGATGCTG	reverse
Tcop16/232 bp	1246A	TATCCCGTCGCTGGAGTAAGA	forward
	1246B	TTACCGCCAAAGCATACATCC	reverse
Tcop17/193 bp	1247A	TATGGTTTTTGCGTGCATGTC	forward
	1247B	ACCATCCGAAGTAGGGAGGAA	reverse
Tcop18/176 bp	1248A	CCACTTTTGGGATCTCCACTG	forward
	1248B	CGCTTCAAGGGAAGTACGATG	reverse
Rpl32/146 bp	DG6I	CTTGAAGCGACGCCTAACTCT	forward
	DG6J	AATGGTTGTCCCCACGGTAAAG	reverse

Table 1 - Primers used for genome walking and RT-qPCR analysis

List of primers used for genome walking and RT-qPCR analysis.

SOURCE	ID	Major Lineage	Phylum	Genus	species	common name	GB ACC#
Tpringle	>LWS.anoCar	C-type	Chordata	Anolis	carolinensis	lizard	XP_003216922
Tpringle	>LWS.galGal	C-type	Chordata	Gallus	gallus	chicken	NP_990740.1
Tpringle	>LWS.gasAcu	C-type	Chordata	Gasterosteus	aculeatus	stickleback	Genome
Tpringle	>LWS.homSap	C-type	Chordata	Homo	sapiens	human	NM_000513
Tpringle	>LWS.letJap	C-type	Chordata	Lethenteron	japonicum	lamprey	BAD17958.1
Tpringle	>LWS.macEug	C-type	Chordata	Macropus	eugenii	wallaby	AAP37945.1
UMBC	>LWS.Macfas	C-type	Chordata	Macaca	fascicularis	macaque	AAD40324.1
Tpringle	>LWS.monDom	C-type	Chordata	Monodelphis	domesticus	opossum	ABC75816.1
Tpringle	>LWS.neoFor	C-type	Chordata	Neoceratodus	forsteri	lungfish	ABS89280.1
Tpringle	>LWS.ornAna	C-type	Chordata	Ornithorhynchus	anatinus	platypus	NP_001121097.1
Tpringle	>LWS.petMar	C-type	Chordata	Petromyzon	marinus	lamprey	ACB69762.1

Tpringle	>LWS.smiCra	C-type	Chordata	Sminthopsis	crassicaudata	dunnart	ACA28596.1
Tpringle	>LWS.takRub	C-type	Chordata	Takifugu	rubripes	fugu	AAT38456.1
UMBC	>LWS.Turtru	C-type	Chordata	Tursiops	truncatus	dolphin	AAC12941.1
Tpringle	>LWS.xenTro	C-type	Chordata	Xenopus	tropicalis	frog	NP_001096331.1
Tpringle	>LWS1.calMil	C-type	Chordata	Callorhinchus	milii	elephantfish	ABU84863.1
Tpringle	>LWS2.calMil	C-type	Chordata	Callorhinchus	milii	elephantfish	ABU84864.1
UMBC	>MWS.Ambtig	C-type	Chordata	Ambystoma	tigrinum	tiger salamander	AAC96070.1
UMBC	>MWS.AstfasRED	C-type	Chordata	Astyanax	fasciatus	mexican cave tetra	AAA02766.1
UMBC	>MWS.AstmexGR	C-type	Chordata	Astyanax	mexicanus	mexican cave tetra	AAA67215.1
UMBC	>MWS.Ategeo	C-type	Chordata	Ateles	geoffroyi	spider monkey	BAD97425.1
UMBC	>MWS.Bostau	C-type	Chordata	Bos	taurus	cow	NP_776991.1
UMBC	>MWS.Caljac1	C-type	Chordata	Callithrix	jacchus	marmoset	BAB58964.1

UMBC	>MWS.Caraur	C-type	Chordata	Carassius	auratus	goldfish	AAA49190.1
UMBC	>MWS.Cavpor	C-type	Chordata	Cavia	porcellus	guinea pig	AAD30523.1
UMBC	>MWS.Cebcap	C-type	Chordata	Cebus	capucinus	monkey	BAD97422.1
UMBC	>MWS.Cercon	C-type	Chordata	Cercartetus	concinnus	possum	AAX11258.1
UMBC	>MWS.Cypcar	C-type	Chordata	Cyprinus	carpio	carp	BAB32496.1
UMBC	>MWS.Danrer.OPN1LW1	C-type	Chordata	Danio	rerio	zebrafish	NP_571250.1
UMBC	>MWS.Danrer.OPN1LW2	C-type	Chordata	Danio	rerio	zebrafish	NP_001002443.1
UMBC	>MWS.Deldel	C-type	Chordata	Delphinus	delphis	dolphin	AAP13020.1
UMBC	>MWS.Enhlut	C-type	Chordata	Enhydra	lutris	sea otter	AAX78226.1
UMBC	>MWS.Equcab	C-type	Chordata	Equus	caballus	horse	NP_001075314.1
UMBC	>MWS.Eribar	C-type	Chordata	Erignathus	barbatus	seal	AAX78227.1
UMBC	>MWS.Felcat	C-type	Chordata	Felis	catus	cat	NP_001009871.1

UMBC	>MWS.Gecgec	C-type	Chordata	Gecko	gecko	gecko	AAA49308.1
UMBC	>MWS.Glomel	C-type	Chordata	Globicephala	melas	whale	AAP13015.1
UMBC	>MWS.Isoobe	C-type	Chordata	Isoodon	obesulus	quenda	AAW69833.1
UMBC	>MWS.Lepwed	C-type	Chordata	Leptonychotes	weddellii	seal	AAX78224.1
UMBC	>MWS.Loxafr	C-type	Chordata	Loxodonta	africana	elephant	AAT95415.1
UMBC	>MWS.Lucgoo	C-type	Chordata	Lucania	goodei	fish	AAP57201.1
UMBC	>MWS.Macfas	C-type	Chordata	Macaca	fascicularis	macaque	AAD40325.1
UMBC	>MWS.Mirang	C-type	Chordata	Mirounga	angustirostris	elephant seal	AAX78223.1
UMBC	>MWS.Musmus	C-type	Chordata	Mus	musculus	mouse	NP_032132.1
UMBC	>MWS.Nanehr	C-type	Chordata	Nannospalax	ehrenbergi	molerat	AAF27288.1
UMBC	>MWS.Odoros	C-type	Chordata	Odobenus	rosmarus	walrus	AAX78220.1
UMBC	>MWS.Odovir	C-type	Chordata	Odocoileus	virginianus	deer	AAD30522.1

UMBC	>MWS.Oncmyk	C-type	Chordata	Oncorhynchus	mykiss	trout	NP_001117792.1
UMBC	>MWS.Oncner	C-type	Chordata	Oncorhynchus	nerka	salmon	AAP58346.1
UMBC	>MWS.Otocra	C-type	Chordata	Otolemur	crassicaudatus	lemur	BAD02407.1
UMBC	>MWS.Phelon	C-type	Chordata	Phelsuma	madagascariensis_longintinue	lizard	AAD25917.1
UMBC	>MWS.Phogro	C-type	Chordata	Phoca	groenlandica	seal	AAP13018.1
UMBC	>MWS.Phopho	C-type	Chordata	Phocoena	phocoena	porpoise	30313997
UMBC	>MWS.Phovit	C-type	Chordata	Phoca	vitulina	seal	AY228448
UMBC	>MWS.Plealt2	C-type	Chordata	Plecoglossus	altivelis	smelt	53828362
UMBC	>MWS.Ratnor	C-type	Chordata	Rattus	norvegicus	rat	16758313
UMBC	>MWS.Salsal	C-type	Chordata	Salmo	salar	salmon	NM_001123705.1
UMBC	>MWS.Scicar	C-type	Chordata	Sciurus	carolinensis	squirrel	4836695
UMBC	>MWS.Setbra	C-type	Chordata	Setonix	brachyurus	quokka	AY745192

UMBC	>MWS.Spetri	C-type	Chordata	Spermophilus	tridecemlineatus	squirrel	56787003
UMBC	>MWS.Susscr	C-type	Chordata	Sus	scrofa	pig	58332859
UMBC	>MWS.Tacacu	C-type	Chordata	Tachyglossus	aculeatus	echidna	189303848
UMBC	>MWS.Taegut	C-type	Chordata	Taeniopygia	guttata	finch	115529263
UMBC	>MWS.Triman	C-type	Chordata	Trichechus	manatus	manatee	AY228447
UMBC	>MWS.Ursmar	C-type	Chordata	Ursus	maritimus	polar bear	62275517
UMBC	>MWS.Xenlae	C-type	Chordata	Xenopus	laevis	frog	1905842
UMBC	>MWS.Zalcal	C-type	Chordata	Zalophus	californianus	sea lion	62275493
Tpringle	>PARIE.anoCar	C-type	Chordata	Anolis	carolinensis	lizard	AAD32622.1
Tpringle	>PARIE.danRer	C-type	Chordata	Danio	rerio	zebrafish	XP_003201482
Tpringle	>PARIE.gasAcu	C-type	Chordata	Gasterosteus	aculeatus	stickleback	Genome
Tpringle	>PARIE.takRub	C-type	Chordata	Takifugu	rubripes	fugu	Genome

Tpringle	>PARIE.utaSta	C-type	Chordata	Uta	stansburiana	lizard	DQ100320
Tpringle	>PARIE.xenTro	C-type	Chordata	Xenopus	tropicalis	frog	NP_001039256.1
Tpringle	>PIN.bufJap	C-type	Chordata	Bufo	japonicus	toad	AF200433
Tpringle	>PIN.colLiv	C-type	Chordata	Columba	livia	pigeon	AAB40945.1
Tpringle	>PIN.galGal	C-type	Chordata	Gallus	gallus	chicken	NP_990740.1
Tpringle	>PIN.pheMad	C-type	Chordata	Phelsuma	madagascariensis	lizard	AB022881
Tpringle	>PIN.podSic	C-type	Chordata	Podarcis	sicula	lizard	DQ013042
Tpringle	>PIN.taeGut	C-type	Chordata	Taeniopygia	guttata	finch	Genome
Tpringle	>PIN.utaSta	C-type	Chordata	Uta	stansburiana	lizard	DQ100321
Tpringle	>PIN.xenTro	C-type	Chordata	Xenopus	tropicalis	frog	XP_002934391.1
Tpringle	>PPIN.anoCar	C-type	Chordata	Anolis	carolinensis	lizard	XP_003217796
Tpringle	>PPIN.danRer	C-type	Chordata	Danio	rerio	zebrafish	NP_001005312.1

Tpringle	>PPIN.ictPun	C-type	Chordata	Ictalurus	punctatus	catfish	AF028014
Tpringle	>PPIN.letJap	C-type	Chordata	Lethenteron	japonicum	lamprey	AB116380
Tpringle	>PPIN.oncMyk	C-type	Chordata	Oncorhynchus	mykiss	trout	BAD17961
Tpringle	>PPIN.xenTro	C-type	Chordata	Xenopus	tropicalis	frog	AAI71331.1
Tpringle	>PPINa.cioInt	C-type	Chordata	Ciona	intestinalis	tunicate	NM_001032555
Tpringle	>PPINa.cioSav	C-type	Chordata	Ciona	savignyi	tunicate	EST library
Tpringle	>PPINa.gasAcu	C-type	Chordata	Gasterosteus	aculeatus	stickleback	DN691173
Tpringle	>PPINa.petMar	C-type	Chordata	Petromyzon	marinus	lamprey	Genome
Tpringle	>PPINa.takRub	C-type	Chordata	Takifugu	rubripes	fugu	Genome
Tpringle	>PPINa.tetNig	C-type	Chordata	Tetraodon	nigroviridis	pufferfish	CAG06878
Tpringle	>PPINb.cioInt	C-type	Chordata	Ciona	intestinalis	tunicate	XP_002119963
Tpringle	>PPINb.cioSav	C-type	Chordata	Ciona	savignyi	tunicate	EST library

Tpringle	>PPINb.gasAcu	C-type	Chordata	Gasterosteus	aculeatus	stickleback	DW621258
Tpringle	>PPINb.takRub	C-type	Chordata	Takifugu	rubripes	fugu	Genome
Tpringle	>PPINb.tetNig	C-type	Chordata	Tetraodon	nigroviridis	pufferfish	Genome
UMBC	>Rh1.Ambtig	C-type	Chordata	Ambystoma	tigrinum	tiger salamander	1477471
UMBC	>RH1.Anapla	C-type	Chordata	Anas	platyrhynchos	duck	AF021240
UMBC	>Rh1.Astmex	C-type	Chordata	Astyanax	mexicanus	mexican cave tetra	AMU12328
UMBC	>Rh1.Bufbuf	C-type	Chordata	Bufo	bufo	toad	2734705
UMBC	>Rh1.Bufmar	C-type	Chordata	Bufo	marinus	toad	2734707
UMBC	>RH1.Calphi	C-type	Chordata	Caluromys	philander	opossum	AY313946
UMBC	>Rh1.Canfam	C-type	Chordata	Canis	familiaris	dog	311989
UMBC	>Rh1.Caraur	C-type	Chordata	Carassius	auratus	goldfish	213016
UMBC	>Rh1.Ccar	C-type	Chordata	Cyprinus	carpio	carp	2598279

UMBC	>Rh1.Chedad	C-type	Chordata	Chela	dadiburjori	dadio fish	EF452915
UMBC	>Rh1.Danrer	C-type	Chordata	Danio	rerio	zebrafish	197246964
UMBC	>Rh1.Deldel	C-type	Chordata	Delphinus	delphis	dolphin	AF055314
UMBC	>Rh1.Dipvul	C-type	Chordata	Diplodus	vulgaris	seabream	4210636
UMBC	>RH1.Enhlut	C-type	Chordata	Enhydra	lutris	sea otter	AY883931
UMBC	>RH1.Eribar	C-type	Chordata	Erignathus	barbatus	seal	AY883932
UMBC	>Rh1.Felcat	C-type	Chordata	Felis	catus	cat	NM_001009242.1
UMBC	>Rh1.Gadmor	C-type	Chordata	Gadus	morhua	cod	33306307
UMBC	>Rh1.Galmel	C-type	Chordata	Galeus	melastomus	catshark	Y17586
UMBC	>Rh1.Gasspi	C-type	Chordata	Garra	spilota	fish	EU409649
UMBC	>Rh1.Glomel	C-type	Chordata	Globicephala	melas	whale	AF055315
UMBC	>Rh1.Gobnig	C-type	Chordata	Gobius	niger	goby	4210640

UMBC	>Rh1.Hiphip	C-type	Chordata	Hippoglossus	hippoglossus	halibut	20269371
UMBC	>RH1.Hydlep	C-type	Chordata	Hydrurga	leptonyx	seal	AY883930
UMBC	>RH1.Lepwed	C-type	Chordata	Leptonychotes	weddellii	seal	AY883929
UMBC	>Rh1.Loxafr	C-type	Chordata	Loxodonta	africana	elephant	51101220
UMBC	>Rh1.Macfas	C-type	Chordata	Macaca	fascicularis	macaque	913368
UMBC	>Rh1.Mesbid	C-type	Chordata	Mesoplodon	bidens	whale	AF055316
UMBC	>Rh1.Mirang	C-type	Chordata	Mirounga	angustirostris	elephant seal	AY228452
UMBC	>Rh1.Musmus	C-type	Chordata	Mus	musculus	mouse	21594394
UMBC	>Rh1.Notath	C-type	Chordata	Notropis	atherinoides	shiner	EF452903
UMBC	>RH1.Odoros	C-type	Chordata	Odobenus	rosmarus	walrus	AY883925
UMBC	>Rh1.Orycun	C-type	Chordata	Oryctolagus	cuniculus	rabbit	NM_001082349.1
UMBC	>Rh1.Otcra	C-type	Chordata	Otolemur	crassicaudatus	lemur	AB112591

UMBC	>Rh1.Parinn	C-type	Chordata	Paracheirodon	innesi	tetra	84095053
UMBC	>Rh1.Phogro	C-type	Chordata	Phoca	groenlandica	seal	AF055318
UMBC	>Rh1.Phovit	C-type	Chordata	Phoca	vitulina	seal	AF055317
UMBC	>Rh1.Poeret	C-type	Chordata	Poecilia	reticulata	guppy	1841472
UMBC	>Rh1.Pseame	C-type	Chordata	Pseudopleuronectes	americanus	flounder	50254000
UMBC	>Rh1.Ratnor	C-type	Chordata	Rattus	norvegicus	rat	U22180
UMBC	>Rh1.Sarpil	C-type	Chordata	Sardina	pilchardus	sardine	Y18677
UMBC	>Rh1.Scoana	C-type	Chordata	Scopelarchus	analis	pearleye	EF517404
UMBC	>RH1.Smicra	C-type	Chordata	Sminthopsis	crassicaudata	dunnart	AAN86046.2
UMBC	>Rh1.Spaehr	C-type	Chordata	Spalax	ehrenbergi	mole rat	10954028
UMBC	>Rh1.Susscr	C-type	Chordata	Sus	scrofa	pig	47523517
UMBC	>Rh1.Taegut	C-type	Chordata	Taeniopygia	guttata	finch	115529257

UMBC	>Rh1.Triman	C-type	Chordata	Trichechus	manatus	manatee	AF055319
UMBC	>Rh1.Turtru	C-type	Chordata	Tursiops	truncatus	dolphin	AF055456
UMBC	>RH1.Ursmar	C-type	Chordata	Ursus	maritimus	polar bear	AY883926
UMBC	>Rh1.Xenlae	C-type	Chordata	Xenopus	laevis	frog	214734
UMBC	>Rh1.Zactem	C-type	Chordata	Zacco	temminckii	chub	EF452918
UMBC	>RH1.Zalcal	C-type	Chordata	Zalophus	californianus	sea lion	AY883924
UMBC	>RH2.Acabut.Rh2Aa	C-type	Chordata	Acanthopagrus	butcheri	black bream	EU090913
UMBC	>RH2.Acabut.Rh2Ab	C-type	Chordata	Acanthopagrus	butcheri	black bream	EU090914
UMBC	>RH2.Anapla	C-type	Chordata	Anas	platyrhynchos	duck	2781422
UMBC	>RH2.Caraur	C-type	Chordata	Carassius	auratus	goldfish	212959
UMBC	>RH2.Colliv	C-type	Chordata	Columba	livia	pigeon	AAD32242.1
UMBC	>RH2.Cypcar1	C-type	Chordata	Cyprinus	carpio	carp	42627736

UMBC	>RH2.Cypcar2	C-type	Chordata	Cyprinus	carpio	carp	31071631
UMBC	>RH2.Dismaw	C-type	Chordata	Dissostichus	mawsoni	toothfish	55585550
UMBC	>RH2.Melund	C-type	Chordata	Melopsittacus	undulatus	budgerigar	AF021241
UMBC	>RH2.Notang	C-type	Chordata	Notothenia	angustata	cod	AY771354
UMBC	>RH2.Pagbor	C-type	Chordata	Pagothenia	borchgrevinki	rockcod	AY771353
UMBC	>RH2.Pseame	C-type	Chordata	Pseudopleuronectes	americanus	flounder	50254002
UMBC	>RH2.Scoana	C-type	Chordata	Scopelarchus	analis	pearleye	EF517406
UMBC	>RH2.Sercan	C-type	Chordata	Serinus	canaria	canary	7981417
UMBC	>RH2.Thuori	C-type	Chordata	Thunnus	orientalis	tuna	AB290451
UMBC	>RH2.Utasta	C-type	Chordata	Uta	stansburiana	lizard	DQ100324
Tpringle	>RHO1.angAng	C-type	Chordata	Anguilla	anguilla	european eel	ACT34384.1
Tpringle	>RHO1.anoCar	C-type	Chordata	Anolis	carolinensis	lizard	P41591.1

Tpringle	>RHO1.bosTau	C-type	Chordata	Bos	taurus	cow	NM_001014890
Tpringle	>RHO1.calMil	C-type	Chordata	Callorhinchus	milii	elephantfish	ABU84865.1
Tpringle	>RHO1.conMyr	C-type	Chordata	Conger	myriaster	conger eel	BAB21486.1
Tpringle	>RHO1.galGal	C-type	Chordata	Gallus	gallus	chicken	NM_205490
Tpringle	>RHO1.geoAus	C-type	Chordata	Geotria	australis	lamprey	AY366493
Tpringle	>RHO1.homSap	C-type	Chordata	Homo	sapiens	human	NM_000539
Tpringle	>RHO1.latCha	C-type	Chordata	Latimeria	chalumnae	coelacanth	AAD30519
Tpringle	>RHO1.letJap	C-type	Chordata	Lethenteron	japonicum	lamprey	AB116382
Tpringle	>RHO1.leuEri	C-type	Chordata	Leucoraja	erinacea	skate	U81514
Tpringle	>RHO1.monDom	C-type	Chordata	Monodelphis	domesticus	opossum	XP_001366225.1
Tpringle	>RHO1.neoFor	C-type	Chordata	Neoceratodus	forsteri	lungfish	ABS89278.1
Tpringle	>RHO1.ornAna	C-type	Chordata	Ornithorhynchus	anatinus	platypus	ABN43074

Tpringle	>RHO1.petMar	C-type	Chordata	Petromyzon	marinus	lamprey	Q98980.1
Tpringle	>RHO1.takRub	C-type	Chordata	Takifugu	rubripes	fugu	AF201472
Tpringle	>RHO1.xenTro	C-type	Chordata	Xenopus	tropicalis	frog	NP_001090803.1
Tpringle	>RHO2.ancDan	C-type	Chordata	Danio	rerio	zebrafish	Genome
Tpringle	>RHO2.anoCar	C-type	Chordata	Anolis	carolinensis	lizard	AAB35062.1
Tpringle	>RHO2.calMil	C-type	Chordata	Callorhinchus	milii	elephantfish	EF565168
Tpringle	>RHO2.galGal	C-type	Chordata	Gallus	gallus	chicken	NP_990821
Tpringle	>RHO2.gasAcu	C-type	Chordata	Gasterosteus	aculeatus	stickleback	Genome
Tpringle	>RHO2.geoAus	C-type	Chordata	Geotria	australis	lamprey	AY366494
Tpringle	>RHO2.hipHip	C-type	Chordata	Hippoglossus	hippoglossus	halibut	AF156263
Tpringle	>RHO2.latCha	C-type	Chordata	Latimeria	chalumnae	coelacanth	AH007713
Tpringle	>RHO2.mulSur	C-type	Chordata	Mullus	surmuletus	mullet	Y18680

Tpringle	>RHO2.neoFor	C-type	Chordata	Neoceratodus	forsteri	lungfish	ABS89279.1
Tpringle	>RHO2.oreNil	C-type	Chordata	Oreochromis	niloticus	tilapia	AF247124
Tpringle	>RHO2.oryLat	C-type	Chordata	Oryzias	latipes	medaka	NP_001098125
Tpringle	>RHO2.pheMad	C-type	Chordata	Phelsuma	madagascariensis	lizard	AF074044
Tpringle	>RHO2.podSic	C-type	Chordata	Podarcis	sicula	lizard	AY941829
Tpringle	>RHO2.pomMin	C-type	Chordata	Pomatoschistus	minutus	sand goby	Y18679
Tpringle	>RHO2.taeGut	C-type	Chordata	Taeniopygia	guttata	finch	NM_001076696
Tpringle	>RHO2.takRub	C-type	Chordata	Takifugu	rubripes	fugu	AF226989
Tpringle	>RHO2a.danRer	C-type	Chordata	Danio	rerio	zebrafish	NM_131253
Tpringle	>RHO2b.danRer	C-type	Chordata	Danio	rerio	zebrafish	NM_182891
Tpringle	>RHO2c.danRer	C-type	Chordata	Danio	rerio	zebrafish	NM_182892
Tpringle	>RHO2d.danRer	C-type	Chordata	Danio	rerio	zebrafish	NM_131254

UMBC	>SWS1.Acabut	C-type	Chordata	Acanthopagrus	butcheri	black bream	ABC75599
UMBC	>SWS1.Alopal	C-type	Chordata	Alouatta	palliata	howler monkey	AAB94764
UMBC	>SWS1.Ambtig	C-type	Chordata	Ambystoma	tigrinum	tiger salamander	AAC96071
Tpringle	>SWS1.anoCar	C-type	Chordata	Anolis	carolinensis	lizard	AAD32621.1
UMBC	>SWS1.Aulhue	C-type	Chordata	Aulonocara	hueseri	cichlid	AAY26075
UMBC	>SWS1.Botau	C-type	Chordata	Bos	taurus	cow	NP_776992
UMBC	>SWS1.Canfam	C-type	Chordata	Canis	familiaris	dog	XP_539386
UMBC	>SWS1.Caraur	C-type	Chordata	Carassius	auratus	goldfish	Q90309
UMBC	>SWS1.Cavpor	C-type	Chordata	Cavia	porcellus	guinea pig	AAT36312
UMBC	>SWS1.Ceboli	C-type	Chordata	Cebus	olivaceus	monkey	AAB95487
UMBC	>SWS1.Colliv	C-type	Chordata	Columba	livia	pigeon	AAD38034
UMBC	>SWS1.Copbor	C-type	Chordata	Copadichromis	borleyi	cichlid	AAY26081

UMBC	>SWS1.Cynafr	C-type	Chordata	Cynotilapia	afra	cichlid	AAY26079
UMBC	>SWS1.Cynpyr	C-type	Chordata	Cynops	pyrrhogaster	newt	BAB79499
UMBC	>SWS1.Cypcar	C-type	Chordata	Cyprinus	carpio	carp	BAC78823
Tpringle	>SWS1.danRer	C-type	Chordata	Danio	rerio	zebrafish	NP_571394.1
UMBC	>SWS1.Daumad	C-type	Chordata	Daubentonia	madagascariensis	aye-aye	ABR53744
UMBC	>SWS1.Dimcom	C-type	Chordata	Dimidiochromis	compressiceps	cichlid	AAF05731
UMBC	>SWS1.Dismaw	C-type	Chordata	Dissostichus	mawsoni	toothfish	AAX99140
UMBC	>SWS1.Equcab	C-type	Chordata	Equus	caballus	horse	XP_001502785
UMBC	>SWS1.Eulful	C-type	Chordata	Eulemur	fulvus	lemur	BAD14291
Tpringle	>SWS1.galGal	C-type	Chordata	Gallus	gallus	chicken	NP_990769.1
UMBC	>SWS1.Gekgek	C-type	Chordata	Gecko	gecko	gecko	AAG61163
Tpringle	>SWS1.geoAus	C-type	Chordata	Geotria	australis	lamprey	AY366495

UMBC	>SWS1.Gymacu	C-type	Chordata	Gymnodraco	acuticeps	fish	AAX99145
UMBC	>SWS1.Hiphip	C-type	Chordata	Hippoglossus	hippoglossus	halibut	AF156264
Tpringle	>SWS1.homSap	C-type	Chordata	Homo	sapiens	human	NP_001699
UMBC	>SWS1.Isoobe	C-type	Chordata	Isoodon	obesulus	quenda	AAU29518
UMBC	>SWS1.Labchi	C-type	Chordata	Labidochromis	chisumulae	cichlid	AAY26073
UMBC	>SWS1.Lepfit	C-type	Chordata	Lepidopus	fitchi	scabbardfish	ACJ72019
UMBC	>SWS1.Letpar	C-type	Chordata	Lethrinops	parvidens	cichlid	AAY26077
UMBC	>SWS1.Loxafr	C-type	Chordata	Loxodonta	africana	elephant	AAT95414
UMBC	>SWS1.Lucgoo	C-type	Chordata	Lucania	goodei	fish	AAP57195
UMBC	>SWS1.Maceug	C-type	Chordata	Macropus	eugenii	wallaby	AAP37944
UMBC	>SWS1.Macmul	C-type	Chordata	Macaca	mulatta	rhesus monkey	XP_001091869
UMBC	>SWS1.Melaur	C-type	Chordata	Melanochromis	auratus	cichlid	AAY26076

UMBC	>SWS1.Melund	C-type	Chordata	Melopsittacus	undulatus	budgerigar	O57605
UMBC	>SWS1.Melver	C-type	Chordata	Melanochromis	vermivorus	cichlid	AAZ53203
Tpringle	>SWS1.monDom	C-type	Chordata	Monodelphis	domesticus	opossum	NP_001138556.1
UMBC	>SWS1.Mumus	C-type	Chordata	Mus	musculus	mouse	NP_031564
Tpringle	>SWS1.neoFor	C-type	Chordata	Neoceratodus	forsteri	lungfish	EF526298.1
UMBC	>SWS1.Neoomn	C-type	Chordata	Neochromis	omnicaeruleus	cichlid	AAV93292
UMBC	>SWS1.Notang	C-type	Chordata	Notothenia	angustata	cod	AAX99143
UMBC	>SWS1.Oncmyk	C-type	Chordata	Oncorhynchus	mykiss	trout	NP_001117793
UMBC	>SWS1.Oncner	C-type	Chordata	Oncorhynchus	nerka	salmon	AAP58349
UMBC	>SWS1.Ophven	C-type	Chordata	Ophthalmotilapia	ventralis	cichlid	AAY26072
UMBC	>SWS1.Orenil	C-type	Chordata	Oreochromis	niloticus	tilapia	AAF05732
Tpringle	>SWS1.oryLat	C-type	Chordata	Oryzias	latipes	medaka	NM_001104656.1

UMBC	>SWS1.Pagbor	C-type	Chordata	Pagothenia	borchgrevinki	rockcod	AAX99142
UMBC	>SWS1.Pagmac	C-type	Chordata	Pagetopsis	macropterus	fish	AAX99144
Tpringle	>SWS1.petMar	C-type	Chordata	Geotria	australis	lamprey	AAR14684
UMBC	>SWS1.Phacar	C-type	Chordata	Phalacrocorax	carbo	cormorant	ABS86975
UMBC	>SWS1.Phemad	C-type	Chordata	Phelsuma	madagascariensis_longintinue	lizard	AAD45183
UMBC	>SWS1.PlealtUV1	C-type	Chordata	Plecoglossus	altivelis	smelt	BAD54746
UMBC	>SWS1.PlealtUV2	C-type	Chordata	Plecoglossus	altivelis	smelt	BAD54747
UMBC	>SWS1.Poeret	C-type	Chordata	Poecilia	reticulata	guppy	ABB69699
UMBC	>SWS1.Pseace	C-type	Chordata	Pseudotropheus	acei	cichlid	AAZ53202
UMBC	>SWS1.Punazu	C-type	Chordata	Pundamilia	azurea	cichlid	AAV93287
UMBC	>SWS1.Punnye	C-type	Chordata	Pundamilia	nyererei	cichlid	AAV93285
UMBC	>SWS1.Rancat	C-type	Chordata	Rana	catesbeiana	frog	BAA96828

UMBC	>SWS1.Ratnor	C-type	Chordata	Rattus	norvegicus	rat	EDM15216
UMBC	>SWS1.Saimbol	C-type	Chordata	Saimiri	boliviensis	monkey	O13092
UMBC	>SWS1.Salsal	C-type	Chordata	Salmo	salar	salmon	NP_001117180
UMBC	>SWS1.Scicar	C-type	Chordata	Sciurus	carolinensis	squirrel	ABC26408
UMBC	>SWS1.Scomax	C-type	Chordata	Scophthalmus	maximus	fish	AF385825
UMBC	>SWS1.Setbra	C-type	Chordata	Setonix	brachyurus	quokka	AAU29519
Tpringle	>SWS1.smiCra	C-type	Chordata	Sminthopsis	crassicaudata	dunnart	AY442173
UMBC	>SWS1.Sphhum	C-type	Chordata	Spheniscus	humboldti	penguin	CAC20913
UMBC	>SWS1.Steleu	C-type	Chordata	Stenobrachius	leucopsarus	lanternfish	ACJ72020
UMBC	>SWS1.Stimod	C-type	Chordata	Stigmatochromis	modestus	cichlid	AAY26082
UMBC	>SWS1.Susscr	C-type	Chordata	Sus	scrofa	pig	NP_999255
Tpringle	>SWS1.taeGut	C-type	Chordata	Taeniopygia	guttata	finch	NP_001070172.1

UMBC	>SWS1.Tarban	C-type	Chordata	Tarsius	bancanus	tarsier	BAD14290
Tpringle	>SWS1.tarRos	C-type	Chordata	Tarsipes	rostratus	honey possum	AY772472
UMBC	>SWS1.Treloe	C-type	Chordata	Trematomus	loennbergii	fish	AAX99141
UMBC	>SWS1.Trodub	C-type	Chordata	Tropheus	duboisi	cichlid	AAY26074
Tpringle	>SWS1.utaSta	C-type	Chordata	Uta	stansburiana	lizard	DQ100325
Tpringle	>SWS1.xenLae	C-type	Chordata	Xenopus	laevis	frog	AAH84882.1
UMBC	>SWS1.Xentro	C-type	Chordata	Xenopus	tropicalis	frog	NP_001119548
UMBC	>SWS2.Ambtig	C-type	Chordata	Ambystoma	tigrinum	tiger salamander	AAC96069
Tpringle	>SWS2.anoCar	C-type	Chordata	Anolis	carolinensis	lizard	AAD32621.1
UMBC	>SWS2.Astbur	C-type	Chordata	Astatotilapia	burtoni	tilapia	AAT79866
UMBC	>SWS2.Astmex	C-type	Chordata	Astyanax	mexicanus	mexican cave tetra	P51472
UMBC	>SWS2.Batnik	C-type	Chordata	Batrachocottus	nikolskii	sculpin	CAD23113

UMBC	>SWS2.Caraur	C-type	Chordata	Carassius	auratus	goldfish	P32310
UMBC	>SWS2.Colliv	C-type	Chordata	Columba	livia	pigeon	AAD38035
UMBC	>SWS2.Cotgob	C-type	Chordata	Cottus	gobio	sculpin	CAD23111
UMBC	>SWS2.Cotine	C-type	Chordata	Cottocomephorus	inermis	sculpin	CAD23114
UMBC	>SWS2.Cotkes	C-type	Chordata	Cottus	kesslerii	sculpin	CAD23115
UMBC	>SWS2.Cynafr2A	C-type	Chordata	Cynotilapia	afra	cichlid	AAY26046
UMBC	>SWS2.Cynpyr	C-type	Chordata	Cynops	pyrrhogaster	newt	BAB39378
UMBC	>SWS2.Cypcar	C-type	Chordata	Cyprinus	carpio	carp	BAC78822
UMBC	>SWS2.Cypeur	C-type	Chordata	Cyphocottus	eurystomus	fish	CAD23112
UMBC	>SWS2.Danrer	C-type	Chordata	Danio	rerio	zebrafish	NP_571267
UMBC	>SWS2.Gadmor	C-type	Chordata	Gadus	morhua	cod	AAQ02799
Tpringle	>SWS2.galGal	C-type	Chordata	Gallus	gallus	chicken	NP_990848

Tpringle	>SWS2.gasAcu	C-type	Chordata	Gasterosteus	aculeatus	stickleback	Genome
Tpringle	>SWS2.geoAus	C-type	Chordata	Geotria	australis	lamprey	AY366492
UMBC	>SWS2.Hiphip	C-type	Chordata	Hippoglossus	hippoglossus	halibut	AAM17920
UMBC	>SWS2.Labchi1	C-type	Chordata	Labidochromis	chisumulae	cichlid	AAY26048
UMBC	>SWS2.Labchi2	C-type	Chordata	Labidochromis	chisumulae	cichlid	AAY26051
UMBC	>SWS2.Copbor2A	C-type	Chordata	Copadichromis	borleyi	cichlid	AAY26039
UMBC	>SWS2.Gekgek	C-type	Chordata	Gekko	gekko	gecko	P35357
UMBC	>SWS2.Letpar2A	C-type	Chordata	Lethrinops	parvidens	cichlid	AAY26044
UMBC	>SWS2.Lucgoo1	C-type	Chordata	Lucania	goodei	fish	AAP57196
UMBC	>SWS2.Lucgoo2	C-type	Chordata	Lucania	goodei	fish	AAP57197
UMBC	>SWS2.Mayzeb2A	C-type	Chordata	Maylandia	zebra	cichlid	AAF63525
UMBC	>SWS2.Melaur2A	C-type	Chordata	Melanochromis	auratus	cichlid	AAY26043

UMBC	>SWS2.Myllat2A	C-type	Chordata	Mylochromis	lateristriga	cichlid	AAY26042
UMBC	>SWS2.Neobri	C-type	Chordata	Neolamprologus	brichardi	cichlid	AAY26049
Tpringle	>SWS2.neoFor	C-type	Chordata	Neoceratodus	forsteri	lungfish	EF526299
UMBC	>SWS2.Oncmyk	C-type	Chordata	Oncorhynchus	mykiss	trout	NP_001117794
UMBC	>SWS2.Oncner	C-type	Chordata	Oncorhynchus	nerka	salmon	AAP58350
UMBC	>SWS2.Ophven2A	C-type	Chordata	Ophthalmotilapia	ventralis	cichlid	AAY26038
UMBC	>SWS2.Ophven2B	C-type	Chordata	Ophthalmotilapia	ventralis	cichlid	AAY26050
UMBC	>SWS2.Orenil2A	C-type	Chordata	Oreochromis	niloticus	tilapia	AAF63527
UMBC	>SWS2.Orenil2B	C-type	Chordata	Oreochromis	niloticus	tilapia	AAF63531
Tpringle	>SWS2.ornAna	C-type	Chordata	Ornithorhynchus	anatinus	platypus	NP_001121098.1
UMBC	>SWS2.Orylat1	C-type	Chordata	Oryzias	latipes	medaka	BAE78650
UMBC	>SWS2.Orylat2	C-type	Chordata	Oryzias	latipes	medaka	BAE78651

UMBC	>SWS2.Parcya	C-type	Chordata	Paralabidochromis	cyaneus	cichlid	AAV93274
UMBC	>SWS2.Poeret	C-type	Chordata	Poecilia	reticulata	guppy	ABB69698
UMBC	>SWS2.Pseace	C-type	Chordata	Pseudotropheus	acei	cichlid	AAZ53199
UMBC	>SWS2.Pseame	C-type	Chordata	Pseudopleuronectes	americanus	flounder	AAT72125
UMBC	>SWS2.Punnye	C-type	Chordata	Pundamilia	nyererei	cichlid	AAV93275
UMBC	>SWS2.Punpun	C-type	Chordata	Pundamilia	pundamilia	cichlid	BAG56606
UMBC	>SWS2.Rancat	C-type	Chordata	Rana	catesbeiana	frog	BAA76864
UMBC	>SWS2.Salsal	C-type	Chordata	Salmo	salar	salmon	NP_001117178
UMBC	>SWS2.Sercan	C-type	Chordata	Serinus	canaria	canary	CAB91994
UMBC	>SWS2.Tacacu	C-type	Chordata	Tachyglossus	aculeatus	echidna	ACD85829
Tpringle	>SWS2.taeGut	C-type	Chordata	Taeniopygia	guttata	finch	NP_001070172.1
Tpringle	>SWS2.takRub	C-type	Chordata	Takifugu	rubripes	fugu	AAT38459.1

UMBC	>SWS2.Tetnig	C-type	Chordata	Tetraodon	nigroviridis	pufferfish	AAT38460
UMBC	>SWS2.Thuori	C-type	Chordata	Thunnus	orientalis	tuna	BAG14282
Tpringle	>SWS2.utaSta	C-type	Chordata	Uta	stansburiana	lizard	DQ100326
Tpringle	>SWS2.xenTro	C-type	Chordata	Xenopus	tropicalis	frog	XP_002937272
Tpringle	>VAOP.anoCar	C-type	Chordata	Anolis	carolinensis	lizard	XP_003218607
UMBC	>VAOP.Astbur	C-type	Chordata	Astatotilapia	burtoni	tilapia	EU523854
UMBC	>VAOP.Caraur	C-type	Chordata	Carassius	auratus	goldfish	AB383149
UMBC	>VAOP.Cypcar	C-type	Chordata	Cyprinus	carpio	carp	AF233520
Tpringle	>VAOP.danRer	C-type	Chordata	Danio	rerio	zebrafish	NM_131586
UMBC	>VAOP.Danrer.valopb	C-type	Chordata	Danio	rerio	zebrafish	AY996588
Tpringle	>VAOP.galGal	C-type	Chordata	Gallus	gallus	chicken	ABM66817.2
UMBC	>VAOP.Orylat	C-type	Chordata	Oryzias	latipes	medaka	NM 001136515

Tpringle	>VAOP.petMar	C-type	Chordata	Petromyzon	marinus	lamprey	U90667
UMBC	>VAOP.Plealt.val	C-type	Chordata	Plecoglossus	altivelis	smelt	AB074483
Tpringle	>VAOP.rutRut	C-type	Chordata	Rutilus	rutilus	minnow	AY116411
UMBC	>VAOP.Salsal	C-type	Chordata	Salmo	salar	salmon	NM 001123626
Tpringle	>VAOP.taeGut	C-type	Chordata	Taeniopygia	guttata	finch	Genome
Tpringle	>VAOP.takRub	C-type	Chordata	Takifugu	rubripes	fugu	Genome
Tpringle	>VAOP.xenTro	C-type	Chordata	Xenopus	tropicalis	frog	XP_002936846.1
Tpringle	>ENCEPH.anoCar	C-type	Chordata	Anolis	carolinensis	lizard	XP_003215888
Tpringle	>ENCEPH.danRer	C-type	Chordata	Danio	rerio	zebrafish	NM_001111164
Tpringle	>ENCEPH.galGal	C-type	Chordata	Gallus	gallus	chicken	XP_426139
Tpringle	>ENCEPH.gasAcu	C-type	Chordata	Gasterosteus	aculeatus	stickleback	Genome
Tpringle	>ENCEPH.homSap	C-type	Chordata	Homo	sapiens	human	NM_014322

Tpringle	>ENCEPH.loxAfr	C-type	Chordata	Loxodonta	africana	elephant	Genome
Tpringle	>ENCEPH.monDom	C-type	Chordata	Monodelphis	domestica	opossum	NC_008802.1
Tpringle	>ENCEPH.musMus	C-type	Chordata	Mus	musculus	mouse	AF140241
Tpringle	>ENCEPH.oryLat	C-type	Chordata	Oryzias	latipes	medaka	Genome
Tpringle	>ENCEPH.otoGar	C-type	Chordata	Otolemur	garnettii	lemur	Genome
Tpringle	>ENCEPH.pteVam	C-type	Chordata	Pteropus	vampyrus	macrobat	Genome
Tpringle	>ENCEPH.takRub	C-type	Chordata	Takifugu	rubripes	fugu	Genome
Tpringle	>ENCEPH4.braBel	C-type	Chordata	Branchiostoma	belcheri	amphioxus	AB050608
Tpringle	>ENCEPH4.braFlo	C-type	Chordata	Branchiostoma	floridae	amphioxus	AB050608
Tpringle	>TMT.anoCar	C-type	Chordata	Anolis	carolinensis	lizard	Genome
Tpringle	>TMT.danRer	C-type	Chordata	Danio	rerio	zebrafish	NP_001112371.1
Tpringle	>TMT.galGal	C-type	Chordata	Gallus	gallus	chicken	XM_001234388

Tpringle	>TMT.gasAcu	C-type	Chordata	Gasterosteus	aculeatus	stickleback	Genome
Tpringle	>TMT.macEug	C-type	Chordata	Macropus	eugenii	wallaby	Genome
Tpringle	>TMT.monDom	C-type	Chordata	Monodelphis	domestica	opossum	XM_001372110
Tpringle	>TMT.ornAna	C-type	Chordata	Ornithorhynchus	anatinus	platypus	Genome
Tpringle	>TMT.oryLat	C-type	Chordata	Oryzias	latipes	medaka	DK170580
Tpringle	>TMT.taeGut	C-type	Chordata	Taeniopygia	guttata	finch	NC_011462.1
Tpringle	>TMT.takRub	C-type	Chordata	Takifugu	rubripes	fugu	AF349945_1
Tpringle	>TMT.tetNig	C-type	Chordata	Tetraodon	nigroviridis	pufferfish	Genome
Tpringle	>TMT.xenTro	C-type	Chordata	Xenopus	tropicalis	frog	AL773998
Tpringle	>TMT5.braBel	C-type	Chordata	Branchiostoma	belcheri	amphioxus	AB050609
Tpringle	>TMT5.braFlo	C-type	Chordata	Branchiostoma	floridae	amphioxus	XM_002589165
Tpringle	>TMTa.gasAcu	C-type	Chordata	Gasterosteus	aculeatus	stickleback	Genome

Tpringle	>TMTa.oryLat	C-type	Chordata	Oryzias	latipes	medaka	Genome
Tpringle	>TMTa.pimPro	C-type	Chordata	Pimephales	promelas	minnow	DT200813
Tpringle	>TMTa.takRub	C-type	Chordata	Takifugu	rubripes	fugu	AF402774
Tpringle	>TMTa.tetNig	C-type	Chordata	Tetraodon	nigroviridis	pufferfish	Genome
Tpringle	>TMTa1.danRer	C-type	Chordata	Danio	rerio	zebrafish	NM_001118899
Tpringle	>TMTb.danRer	C-type	Chordata	Danio	rerio	zebrafish	CN506730
Tpringle	>TMTb.gasAcu	C-type	Chordata	Gasterosteus	aculeatus	stickleback	Genome
Tpringle	>TMTb.oryLat	C-type	Chordata	Oryzias	latipes	medaka	Genome
Tpringle	>TMTb.takRub	C-type	Chordata	Takifugu	rubripes	fugu	Genome
Tpringle	>TMTb.tetNig	C-type	Chordata	Tetraodon	nigroviridis	pufferfish	Genome
Tpringle	>TMTPIN.stoPur	C-type	Echinodermata	Strongylocentrotus	purpuratus	sea urchin	XM_001177470
Tpringle	>TMTy.braFlo	C-type	Chordata	Branchiostoma	floridae	amphioxus	FE572481

UMBC	>DIP.Culqui	C-type	Arthropoda	Culex	quinquefasciatus	mosquito	XM_001864516
Tpringle	>TMT.aedAeg	C-type	Arthropoda	Aedes	aegypti	mosquito	XM_001650752
Tpringle	>TMT.apiMel	C-type	Arthropoda	Apis	mellifera	bee	NM_001039968
Tpringle	>TMT.culPip	C-type	Arthropoda	Culex	pipiens	mosquito	XP_001864551
Tpringle	>TMT.triCas	C-type	Arthropoda	Tribolium	castaneum	flour beetle	NP_001138950.1
Tpringle	>TMT1.anoGam	C-type	Arthropoda	Anopheles	gambiae	mosquito	XM_312503
Tpringle	>TMT2.anoGam	C-type	Arthropoda	Anopheles	gambiae	mosquito	XM_312502
Tpringle	>TMTa.dapPul	C-type	Arthropoda	Daphnia	pulex	water flea	Genome
Tpringle	>TMTb.dapPul	C-type	Arthropoda	Daphnia	pulex	water flea	Genome
Tpringle	>ENCEPH.strPur	C-type	Echinodermata	Strongylocentrotus	purpuratus	sea urchin	AAGJ02133080
Tpringle	>TMT1.plaDum	C-type	Annelida	Platynereis	dumerilii	ragworm	CT030681
Tpringle	>TMT2.plaDum	C-type	Annelida	Platynereis	dumerilii	ragworm	AY692353

UMBC	>CNID.CropB1	Cnidops	Cnidaria	Cladonema	radiatum	jellyfish	AB332416
UMBC	>CNID.CropB4	Cnidops	Cnidaria	Cladonema	radiatum	jellyfish	AB332417
UMBC	>CNID.CropC	Cnidops	Cnidaria	Cladonema	radiatum	jellyfish	AB332420
UMBC	>CNID.CropD	Cnidops	Cnidaria	Cladonema	radiatum	jellyfish	AB332422
UMBC	>CNID.CropE	Cnidops	Cnidaria	Cladonema	radiatum	jellyfish	AB332421
UMBC	>CNID.CropF	Cnidops	Cnidaria	Cladonema	radiatum	jellyfish	AB332426
UMBC	>CNID.CropG1	Cnidops	Cnidaria	Cladonema	radiatum	jellyfish	AB332427
UMBC	>CNID.CropH	Cnidops	Cnidaria	Cladonema	radiatum	jellyfish	AB332423
UMBC	>CNID.CropI	Cnidops	Cnidaria	Cladonema	radiatum	jellyfish	AB332424
UMBC	>CNID.CropJ	Cnidops	Cnidaria	Cladonema	radiatum	jellyfish	AB332433
UMBC	>CNID.CropK1	Cnidops	Cnidaria	Cladonema	radiatum	jellyfish	AB332431
UMBC	>CNID.CropK2	Cnidops	Cnidaria	Cladonema	radiatum	jellyfish	AB332432

UMBC	>CNID.CropL	Cnidops	Cnidaria	Cladonema	radiatum	jellyfish	AB332425
UMBC	>CNID.CropM	Cnidops	Cnidaria	Cladonema	radiatum	jellyfish	AB332418
UMBC	>CNID.CropN1	Cnidops	Cnidaria	Cladonema	radiatum	jellyfish	AB332429
UMBC	>CNID.CropO	Cnidops	Cnidaria	Cladonema	radiatum	jellyfish	AB332419
UMBC	>CNID.PcopB (2)	Cnidops	Cnidaria	Podocoryna	carnea	hydrozoan	AB332434
UMBC	>CNID.PcopcC (1)	Cnidops	Cnidaria	Podocoryna	carnea	hydrozoan	AB332435
Tpringle	>CNOPa1.hydMag	Cnidops	Cnidaria	Hydra	magnipapillata	hydra	ACZU01000679
Tpringle	>CNOPa2.hydMag	Cnidops	Cnidaria	Hydra	magnipapillata	hydra	ACZU01004988
Tpringle	>CNOPa3.hydMag	Cnidops	Cnidaria	Hydra	magnipapillata	hydra	ACZU01055709
Tpringle	>CNOPa4.hydMag	Cnidops	Cnidaria	Hydra	magnipapillata	hydra	ACZU01004994
Tpringle	>CNOPa5.hydMag	Cnidops	Cnidaria	Hydra	magnipapillata	hydra	ACZU01004993
Tpringle	>CNOPa6.hydMag	Cnidops	Cnidaria	Hydra	magnipapillata	hydra	ACZU01000199

Tpringle	>CNOPb1.hydMag	Cnidops	Cnidaria	Hydra	magnipapillata	hydra	ACZU01027146
Tpringle	>CNOPb2.hydMag	Cnidops	Cnidaria	Hydra	magnipapillata	hydra	ACZU01051946
Tpringle	>CNOPb3.hydMag	Cnidops	Cnidaria	Hydra	magnipapillata	hydra	ACZU01043783
Tpringle	>CNOPb4.hydMag	Cnidops	Cnidaria	Hydra	magnipapillata	hydra	ACZU01065240
Tpringle	>CNOPb5.hydMag	Cnidops	Cnidaria	Hydra	magnipapillata	hydra	ACZU01078635
Tpringle	>CNOPc1.hydMag	Cnidops	Cnidaria	Hydra	magnipapillata	hydra	ACZU01005587
Tpringle	>CNOPc2.hydMag	Cnidops	Cnidaria	Hydra	magnipapillata	hydra	ACZU01005585
Tpringle	>CNOPc3.hydMag	Cnidops	Cnidaria	Hydra	magnipapillata	hydra	ACZU01005579
Tpringle	>CNOPd1.hydMag	Cnidops	Cnidaria	Hydra	magnipapillata	hydra	ACZU01091217
Tpringle	>CNOPd2.hydMag	Cnidops	Cnidaria	Hydra	magnipapillata	hydra	ACZU01027487
Tpringle	>CNOPd3.hydMag	Cnidops	Cnidaria	Hydra	magnipapillata	hydra	ACZU01075622
Tpringle	>CNOPd4.hydMag	Cnidops	Cnidaria	Hydra	magnipapillata	hydra	ACZU01076129

Tpringle	>CNOPd5.hydMag	Cnidops	Cnidaria	Hydra	magnipapillata	hydra	ACZU01038272
Tpringle	>CNOPd6.hydMag	Cnidops	Cnidaria	Hydra	magnipapillata	hydra	ACZU01049360
Tpringle	>CNOPd7.hydMag	Cnidops	Cnidaria	Hydra	magnipapillata	hydra	ACZU01040368
Tpringle	>CNOPe1.hydMag	Cnidops	Cnidaria	Hydra	magnipapillata	hydra	ACZU01068872
Tpringle	>CNOPe2.hydMag	Cnidops	Cnidaria	Hydra	magnipapillata	hydra	ACZU01085554
Tpringle	>CNOPe3.hydMag	Cnidops	Cnidaria	Hydra	magnipapillata	hydra	ACZU01057385
Tpringle	>CNOPe4.hydMag	Cnidops	Cnidaria	Hydra	magnipapillata	hydra	ACZU01037497
Tpringle	>CNOPe5.hydMag	Cnidops	Cnidaria	Hydra	magnipapillata	hydra	ACZU01037501
Tpringle	>CNOPe6.hydMag	Cnidops	Cnidaria	Hydra	magnipapillata	hydra	ACZU01013912
Tpringle	>CNOPf1.hydMag	Cnidops	Cnidaria	Hydra	magnipapillata	hydra	ACZU01095006
Tpringle	>CNOPf2.hydMag	Cnidops	Cnidaria	Hydra	magnipapillata	hydra	ACZU01063269
Tpringle	>CNOPf3.hydMag	Cnidops	Cnidaria	Hydra	magnipapillata	hydra	ACZU01063264

Tpringle	>CNOPf4.hydMag	Cnidops	Cnidaria	Hydra	magnipapillata	hydra	ACZU01049000
Tpringle	>CNOPg1.hydMag	Cnidops	Cnidaria	Hydra	magnipapillata	hydra	ACZU01024987
Tpringle	>CNOPg2.hydMag	Cnidops	Cnidaria	Hydra	magnipapillata	hydra	ACZU01083619
Tpringle	>CNOPg3.hydMag	Cnidops	Cnidaria	Hydra	magnipapillata	hydra	ACZU01007423
Tpringle	>CNOPg4.hydMag	Cnidops	Cnidaria	Hydra	magnipapillata	hydra	ACZU01103264
Tpringle	>CNOPg5.hydMag	Cnidops	Cnidaria	Hydra	magnipapillata	hydra	ACZU01014309
Tpringle	>CNOPg6.hydMag	Cnidops	Cnidaria	Hydra	magnipapillata	hydra	ACZU01086515
Tpringle	>CNOPh1.hydMag	Cnidops	Cnidaria	Hydra	magnipapillata	hydra	ACZU01018050
Tpringle	>CNOPh2.hydMag	Cnidops	Cnidaria	Hydra	magnipapillata	hydra	ACZU01085207
Tpringle	>CNOPh3.hydMag	Cnidops	Cnidaria	Hydra	magnipapillata	hydra	ACZU01011197
Tpringle	>CNOPh4.hydMag	Cnidops	Cnidaria	Hydra	magnipapillata	hydra	ACZU01090363
Tpringle	>NEUR.strPur	Group 4	Echinodermata	Strongylocentrotus	purpuratus	sea urchin	XM_001197837

Tpringle	>NEUR1.bosTau	Group 4	Chordata	Bos	taurus	cow	XP_615467
Tpringle	>NEUR1.calJac	Group 4	Chordata	Callithrix	jacchus	marmoset	XP_002746669
Tpringle	>NEUR1.canFam	Group 4	Chordata	Canis	familiaris	dog	Genome
Tpringle	>NEUR1.dasNov	Group 4	Chordata	Dasypus	novemcinctus	armadillo	Genome
Tpringle	>NEUR1.galGal	Group 4	Chordata	Gallus	gallus	chicken	NP_001124215
Tpringle	>NEUR1.homSap	Group 4	Chordata	Homo	sapiens	human	NP_859528
Tpringle	>NEUR1.loxAfr	Group 4	Chordata	Loxodonta	africana	elephant	Genome
Tpringle	>NEUR1.monDom	Group 4	Chordata	Monodelphis	domesticus	opossum	XP_001369202
Tpringle	>NEUR1.musMus	Group 4	Chordata	Mus	musculus	mouse	NP_861418
Tpringle	>NEUR1.ornAna	Group 4	Chordata	Ornithorhynchus	anatinus	platypus	XP_001511991.1
Tpringle	>NEUR1.xenTro	Group 4	Chordata	Xenopus	tropicalis	frog	Genome
Tpringle	>NEUR1a.braFlo	Group 4	Chordata	Branchiostoma	floridae	amphioxus	FE548698

Tpringle	>NEUR1b.braFlo	Group 4	Chordata	Branchiostoma	floridae	amphioxus	XM_002202511
Tpringle	>NEUR2.anoCar	Group 4	Chordata	Anolis	carolinensis	lizard	Genome
Tpringle	>NEUR2.danRer	Group 4	Chordata	Danio	rerio	zebrafish	Genome
Tpringle	>NEUR2.galGal	Group 4	Chordata	Gallus	gallus	chicken	AB368181
Tpringle	>NEUR2.xenTro	Group 4	Chordata	Xenopus	tropicalis	frog	Genome
Tpringle	>NEUR3.anoCar	Group 4	Chordata	Anolis	carolinensis	lizard	AAWZ01001057
Tpringle	>NEUR3.galGal	Group 4	Chordata	Gallus	gallus	chicken	AB368183
Tpringle	>NEUR3.taeGut	Group 4	Chordata	Taeniopygia	guttata	finch	ABQF01025032
Tpringle	>NEUR3.xenTro	Group 4	Chordata	Xenopus	tropicalis	frog	Genome
Tpringle	>NEUR3a.danRer	Group 4	Chordata	Danio	rerio	zebrafish	Genome
Tpringle	>NEUR3a.tetNig	Group 4	Chordata	Tetraodon	nigroviridis	pufferfish	Genome
Tpringle	>NEUR3b.danRer	Group 4	Chordata	Danio	rerio	zebrafish	Genome

Tpringle	>NEUR4.anocar	Group 4	Chordata	Anolis	carolinensis	lizard	Genome
Tpringle	>NEUR4.danRer	Group 4	Chordata	Danio	rerio	zebrafish	Genome
Tpringle	>NEUR4.galGal	Group 4	Chordata	Gallus	gallus	chicken	Genome
Tpringle	>NEUR4.gasAcu	Group 4	Chordata	Gasterosteus	aculeatus	stickleback	Genome
Tpringle	>NEUR4.ornAna	Group 4	Chordata	Ornithorhynchus	anatinus	platypus	XP_001508178
Tpringle	>NEUR4.tetNig	Group 4	Chordata	Tetraodon	nigroviridis	pufferfish	Genome
Tpringle	>NEUR4.xenTro	Group 4	Chordata	Xenopus	tropicalis	frog	Genome
UMBC	>OPS5.Equcab	Group 4	Chordata	Equus	caballus	horse	XP_001502825
UMBC	>OPS5.Macmul	Group 4	Chordata	Macaca	mulatta	rhesus monkey	XP_001103952
UMBC	>OPS5.tetnig	Group 4	Chordata	Tetraodon	nigroviridis	pufferfish	CAG13006
Tpringle	>PER1.braBel	Group 4	Chordata	Branchiostoma	belcheri	amphioxus	AB050610
Tpringle	>PER1.braFlo	Group 4	Chordata	Branchiostoma	floridae	amphioxus	AB050606

Tpringle	>PER2.braBel	Group 4	Chordata	Branchiostoma	belcheri	amphioxus	AB050607
Tpringle	>PER2.braFlo	Group 4	Chordata	Branchiostoma	floridae	amphioxus	AB050607
Tpringle	>PER1.aplCal	Group 4	Mollusca	Aplysia	californica	sea slug	EB338056
Tpringle	>PER1.lotGig	Group 4	Mollusca	Lottia	gigantea	limpet	Genome
Tpringle	>PER1a.sacKol	Group 4	Hemichordata	Saccoglossus	kowalevskii	acornworm	ACQM01133041
UMBC	>RGR.Bostau	Group 4	Chordata	Bos	taurus	cow	NP_786969
UMBC	>RGR.Danrer	Group 4	Chordata	Danio	rerio	zebrafish	Q567Y2
UMBC	>RGR.Musmus	Group 4	Chordata	Mus	musculus	mouse	NP_067315
UMBC	>RGR.Ratnor	Group 4	Chordata	Rattus	norvegicus	rat	NP_001100769
Tpringle	>RGR1.galGal	Group 4	Chordata	Gallus	gallus	chicken	NM_001031216
Tpringle	>RGR1.gasAcu	Group 4	Chordata	Gasterosteus	aculeatus	stickleback	Genome
Tpringle	>RGR1.homSap	Group 4	Chordata	Homo	sapiens	human	NM_001012720

Tpringle	>RGR1.xenTro	Group 4	Chordata	Xenopus	tropicalis	frog	BC135113
Tpringle	>RGR2.danRer	Group 4	Chordata	Danio	rerio	zebrafish	NM_001024436
Tpringle	>RGR2.gasAcu	Group 4	Chordata	Gasterosteus	aculeatus	stickleback	Genome
Tpringle	>RGR2.oryLat	Group 4	Chordata	Oryzias	latipes	medaka	Genome
Tpringle	>RGR2.pimPro	Group 4	Chordata	Pimephales	promelas	minnow	EST library
Tpringle	>RGR2.tetNig	Group 4	Chordata	Tetraodon	nigroviridis	pufferfish	Genome
UMBC	>PER.Bostau	Group 4	Chordata	Bos	taurus	cow	NP_001179153
UMBC	>PER.Canfam	Group 4	Chordata	Canis	familiaris	dog	XP 853139
Tpringle	>PER1.gasAcu	Group 4	Chordata	Gasterosteus	aculeatus	stickleback	Genome
Tpringle	>PER1.homSap	Group 4	Chordata	Homo	sapiens	human	NM_006583
Tpringle	>PER1.monDom	Group 4	Chordata	Monodelphis	domestica	opossum	Genome
Tpringle	>PER1.ornAna	Group 4	Chordata	Ornithorhynchus	anatinus	platypus	XM_001506377

Tpringle	>PER1.xenTro	Group 4	Chordata	Xenopus	tropicalis	frog	Genome
Tpringle	>PER3.braBel	Group 4	Chordata	Branchiostoma	belcheri	amphioxus	AB050606
Tpringle	>PER3.braFlo	Group 4	Chordata	Branchiostoma	floridae	amphioxus	AB050610
UMBC	>RRH.Danrer	Group 4	Chordata	Danio	rerio	zebrafish	NP_001004654.1
UMBC	>RRH.Galgal	Group 4	Chordata	Gallus	gallus	chicken	NP_001073227.1
UMBC	>RRH.Musmus	Group 4	Chordata	Mus	musculus	mouse	NP_033128.1
Tpringle	>BCR.limPol	R-type	Arthropoda	Limulus	polyphemus	horseshoe crab	ACO05013
Tpringle	>BCR.triGra	R-type	Arthropoda	Triops	granarius	tadpole shrimp	BAG80976
Tpringle	>BCR1.triGra	R-type	Arthropoda	Triops	granarius	tadpole shrimp	BAG80979
Tpringle	>BCR1.triLon	R-type	Arthropoda	Triops	longicaudatus	tadpole shrimp	BAG80982
Tpringle	>BCR2.braKug	R-type	Arthropoda	Branchinella	kugenumaensis	fairy shrimp	BAG80986
Tpringle	>BCR2.triGra	R-type	Arthropoda	Triops	granarius	tadpole shrimp	BAG80977

Tpringle	>BCR2.triLon	R-type	Arthropoda	Triops	longicaudatus	tadpole shrimp	BAG80981
Tpringle	>BCR3.braKug	R-type	Arthropoda	Branchinella	kugenumaensis	fairy shrimp	BAG80985
Tpringle	>BCR3.triGra	R-type	Arthropoda	Triops	granarius	tadpole shrimp	BAG80980
Tpringle	>BCRa.hemSan	R-type	Arthropoda	Hemigrapsus	sanguineus	crab	D50583.1
Tpringle	>BCRb.hemSan	R-type	Arthropoda	Hemigrapsus	sanguineus	crab	D50584.1
UMBC	>CRUST.Neooer.Rh2	R-type	Arthropoda	Neogonodactylus	oerstedii	stomatopod	DQ646870
UMBC	>DIP.Bacdor	R-type	Arthropoda	Bactrocera	dorsalis	fruitfly	AAS88872
UMBC	>DIP.Cery	R-type	Arthropoda	Calliphora	erythrocephala	blowfly	156511
UMBC	>DIP.Drepse.RH1	R-type	Arthropoda	Drosophila	pseudoobscura	fruitfly	9077
UMBC	>DIP.Dropse.RH2	R-type	Arthropoda	Drosophila	pseudoobscura	fruitfly	9079
UMBC	>DIP.Dropse.RH3	R-type	Arthropoda	Drosophila	pseudoobscura	fruitfly	CAA46710
Tpringle	>LMS.acyPis	R-type	Arthropoda	Acyrthosiphon	pisum	pea aphid	XP_001947730.1

Tpringle	>LMS.anoGam	R-type	Arthropoda	Anopheles	gambiae	mosquito	CAA76825.1
Tpringle	>LMS.homCoa	R-type	Arthropoda	Homalodisca	coagulata	sharpshooter	AY588065
Tpringle	>LMS.ixoSca	R-type	Arthropoda	Ixodes	scapularis	tick	XM_002408275.1
Tpringle	>LMS.limPol	R-type	Arthropoda	Limulus	polyphemus	horseshoe crab	AAA02498.1
Tpringle	>LMS.lucCru	R-type	Arthropoda	Luciola	cruciata	firefly	BAH56227.1
Tpringle	>LMS.manSex	R-type	Arthropoda	Manduca	sexta	moth	AAD11964.1
Tpringle	>LMS.meoOer	R-type	Arthropoda	Neogonodactylus	oerstedii	stomatopod	DQ646869
Tpringle	>LMS.papXut	R-type	Arthropoda	Papilio	xuthus	butterfly	AB028218
Tpringle	>LMS.schGre	R-type	Arthropoda	Schistocerca	gregaria	locust	CAA56377
Tpringle	>LMS.triCas	R-type	Arthropoda	Tribolium	castaneum	flour beetle	ABA00706.1
Tpringle	>LMS1.hasAda	R-type	Arthropoda	Hasarius	adansoni	jumping spider	BAG14330.1
Tpringle	>LMS1.plePay	R-type	Arthropoda	Plexippus	paykulli	jumping spider	BAG14333.1

Tpringle	>LMS2.droMel	R-type	Arthropoda	Drosophila	melanogaster	fruitfly	AAA28734.1
Tpringle	>LMS2.hasAda	R-type	Arthropoda	Hasarius	adansoni	jumping spider	BAG14331.1
UMBC	>LMS2.Limpol	R-type	Arthropoda	Limulus	polyphemus	horseshoe crab	P35361
Tpringle	>LMS2.plePay	R-type	Arthropoda	Plexippus	paykulli	jumping spider	BAG14334.1
Tpringle	>LMS6.droMel	R-type	Arthropoda	Drosophila	melanogaster	fruitfly	CAB06821.1
Tpringle	>LMSa.apiMel	R-type	Arthropoda	Apis	mellifera	bee	NM_001077825
Tpringle	>LMSa.nasVit	R-type	Arthropoda	Nasonia	vitripennis	jewel wasp	NM_001170908.1
Tpringle	>LMSb.apiMel	R-type	Arthropoda	Apis	mellifera	bee	U26026
Tpringle	>LMSb.nasVit	R-type	Arthropoda	Nasonia	vitripennis	jewel wasp	NM_001170908.1
UMBC	>LWS.Acypis	R-type	Arthropoda	Acyrthosiphon	pisum	pea aphid	187892212
UMBC	>LWS.Apicer	R-type	Arthropoda	Apis	cerana	bee	219566008
UMBC	>LWS.Apomor.Rh1	R-type	Arthropoda	Apodemia	mormo	butterfly	50981744

UMBC	>LWS.Apomor.Rh2	R-type	Arthropoda	Apodemia	mormo	butterfly	50981746
UMBC	>LWS.Athros	R-type	Arthropoda	Athalia	rosae	sawfly	188593534
UMBC	>LWS.Bicany	R-type	Arthropoda	Bicyclus	anynana	butterfly	157502893
UMBC	>LWS.Bommor.EO	R-type	Arthropoda	Bombyx	mori	silkworm	112983528
UMBC	>LWS.Camllud	R-type	Arthropoda	Cambarus	ludovicianus	crayfish	CK991518.1
UMBC	>LWS.Catbom	R-type	Arthropoda	Cataglyphis	bombycinus	ant	AF042787.1
UMBC	>LWS.Colphil	R-type	Arthropoda	Colias	philodice	butterfly	62860651
UMBC	>LWS.Geoutr	R-type	Arthropoda	Geoica	utricularia	aphid	219920848
UMBC	>LWS.Helera	R-type	Arthropoda	Heliconius	erato	butterfly	62860665
UMBC	>LWS.Limart	R-type	Arthropoda	Limenitis	arthemis	butterfly	62860657
UMBC	>LWS.Lycrub.Rh1	R-type	Arthropoda	Lycaena	rubidus	butterfly	50981732
UMBC	>LWS.Megvic	R-type	Arthropoda	Megoura	viciae	vetch aphid	AF189714

UMBC	>LWS.Minabi	R-type	Arthropoda	Mindarus	abietinus	aphid	219920842
UMBC	>LWS.Myrlow	R-type	Arthropoda	Myrmecia	loweryi	ant	75755591
UMBC	>LWS.Neorid	R-type	Arthropoda	Neominois	ridingsii	butterfly	115490647
UMBC	>LWS.Notmac	R-type	Arthropoda	Nothomyrmecia	macrops	ant	75755597
UMBC	>LWS.Oenchr	R-type	Arthropoda	Oeneis	chryxus	butterfly	115490649
UMBC	>LWS.Pedhum	R-type	Arthropoda	Pediculus	humanus	louse	AAZO01003609
UMBC	>LWS.Pierap	R-type	Arthropoda	Pieris	rapae	butterfly	BAD06459
UMBC	>LWS.Polica	R-type	Arthropoda	Polyommatus	icarus	butterfly	158512151
UMBC	>LWS.Procla	R-type	Arthropoda	Procambarus	clarkii	crayfish	S53494
UMBC	>LWS.Schgre.Lo2	R-type	Arthropoda	Schistocerca	gregaria	locust	CAA56378.1
UMBC	>LWS.Sphodro	R-type	Arthropoda	Sphodromantis	sp.	praying mantis	312514
UMBC	>LWS.Themar	R-type	Arthropoda	Thermonectus	marmoratus	diving beetle	197259972

UMBC	>LWS.Vancar	R-type	Arthropoda	Vanessa	cardui	butterfly	159798088
UMBC	>SWS.AedaegUV1	R-type	Arthropoda	Aedes	aegypti	mosquito	XP_001662982
UMBC	>SWS.AedaegUV2	R-type	Arthropoda	Aedes	aegypti	mosquito	XP_001653866.1
UMBC	>SWS.Apicer	R-type	Arthropoda	Apis	cerana	bee	BAH04515.1
UMBC	>SWS.ApicerUV	R-type	Arthropoda	Apis	cerana	bee	BAH04514.1
UMBC	>SWS.ApomorB	R-type	Arthropoda	Apodemia	mormo	butterfly	AAT91643.1
UMBC	>SWS.ApomorUV	R-type	Arthropoda	Apodemia	mormo	butterfly	AAT91642.1
UMBC	>SWS.BicanyB	R-type	Arthropoda	Bicyclus	anynana	butterfly	AAY16527.1
UMBC	>SWS.BicanyUV	R-type	Arthropoda	Bicyclus	anynana	butterfly	AAL91507.1
UMBC	>SWS.BomimpUV	R-type	Arthropoda	Bombus	impatiens	bumble bees	AAV67326.1
UMBC	>SWS.CamabdUV	R-type	Arthropoda	Camponotus	abdominalis	ant	AAC05092.1
UMBC	>SWS.CatbomUV	R-type	Arthropoda	Cataglyphis	bombycinus	ant	AAC05091.1

UMBC	>SWS.ColphiV	R-type	Arthropoda	Colias	philodice	butterfly	AAY16532.1
UMBC	>SWS.CulquiUV	R-type	Arthropoda	Culex	quinquefasciatus	mosquito	XP_001851157.1
UMBC	>SWS.Danple	R-type	Arthropoda	Danaus	plexippus	butterfly	AAU07977.1
UMBC	>SWS.DanplexUV	R-type	Arthropoda	Danaus	plexippus	butterfly	AAU07979.1
UMBC	>SWS.Helera	R-type	Arthropoda	Heliconius	erato	butterfly	AAY16539.1
UMBC	>SWS.HeleraUV	R-type	Arthropoda	Heliconius	erato	butterfly	AAY16537.1
UMBC	>SWS.HelmelB	R-type	Arthropoda	Heliconius	melpomene	butterfly	AAY16530.1
UMBC	>SWS.LimartB	R-type	Arthropoda	Limenitis	arthemis astyanax	butterfly	AAY16535.2
UMBC	>SWS.LycrubB1	R-type	Arthropoda	Lycaena	rubidus	butterfly	AAT91639.1
UMBC	>SWS.LycrubB2	R-type	Arthropoda	Lycaena	rubidus	butterfly	AAT91640.1
UMBC	>SWS.LycrubUV	R-type	Arthropoda	Lycaena	rubidus	butterfly	AAT91641.1
UMBC	>SWS.NymantB	R-type	Arthropoda	Nymphalis	antiopa	butterfly	AAY16526.1

UMBC	>SWS.PapxutB	R-type	Arthropoda	Papilio	xuthus	butterfly	BAA93469.1
UMBC	>SWS.Pierap	R-type	Arthropoda	Pieris	rapae	butterfly	BAE19945.1
UMBC	>SWS.PleglaB1	R-type	Arthropoda	Plebejus	glandon	butterfly	ABD64152.1
UMBC	>SWS.PleglaB2	R-type	Arthropoda	Plebejus	glandon	butterfly	ABD64153.1
UMBC	>SWS.PolicaB1	R-type	Arthropoda	Polyommatus	icarus	butterfly	ABD64150.1
UMBC	>SWS.PolicaB2	R-type	Arthropoda	Polyommatus	icarus	butterfly	ABD64151.1
UMBC	>SWS.PolicaUV	R-type	Arthropoda	Polyommatus	icarus	butterfly	ABW69111.1
UMBC	>SWS.SatbehB1	R-type	Arthropoda	Satyrium	behrii	butterfly	ABD64148.1
UMBC	>SWS.SatbehB2	R-type	Arthropoda	Satyrium	behrii	butterfly	ABD64149.1
UMBC	>SWS.ThemarUVI	R-type	Arthropoda	Thermonectus	marmoratus	diving beetle	ACH56537.1
UMBC	>SWS.ThemarUVII	R-type	Arthropoda	Thermonectus	marmoratus	diving beetle	ACH56538.1
UMBC	>SWS.VancarB	R-type	Arthropoda	Vanessa	cardui	butterfly	AAP49026.2

UMBC	>SWS.VancarUV	R-type	Arthropoda	Vanessa	cardui	butterfly	AAP49025.2
Tpringle	>UV3.droMel	R-type	Arthropoda	Drosophila	melanogaster	fruitfly	AAA28854.1
Tpringle	>UV5.anoGam	R-type	Arthropoda	Anopheles	gambiae	mosquito	XP_001688790
Tpringle	>UV5.apiMel	R-type	Arthropoda	Apis	mellifera	bee	AAC13418
Tpringle	>UV5.braKug	R-type	Arthropoda	Branchinella	kugenumaensis	fairy shrimp	BAG80984
Tpringle	>UV5.diaNig	R-type	Arthropoda	Dianemobius	nigrofasciatus	cricket	BAG71429
Tpringle	>UV5.hasAda	R-type	Arthropoda	Hasarius	adansoni	jumping spider	BAG14332.1
Tpringle	>UV5.lucCru	R-type	Arthropoda	Luciola	cruciata	firefly	BAH56228
Tpringle	>UV5.manSex	R-type	Arthropoda	Manduca	sexta	moth	AAD11965.1
Tpringle	>UV5.papXut	R-type	Arthropoda	Papilio	xuthus	butterfly	BAA93470
Tpringle	>UV5.plePay	R-type	Arthropoda	Plexippus	paykulli	jumping spider	BAG14335
Tpringle	>UV5.rhoPro	R-type	Arthropoda	Rhodnius	prolixus	kissing bug	Genome

Tpringle	>UV5.triGra	R-type	Arthropoda	Triops	granarius	tadpole shrimp	BAG80978
Tpringle	>UV5.triLon	R-type	Arthropoda	Triops	longicaudatus	tadpole shrimp	BAG80983
Tpringle	>UV5B.droMel	R-type	Arthropoda	Drosophila	melanogaster	fruitfly	AAC47426.1
Tpringle	>UV7.aedAeg	R-type	Arthropoda	Aedes	aegypti	mosquito	XM_001650694
Tpringle	>UV7.anoGam	R-type	Arthropoda	Anopheles	gambiae	mosquito	XP_308329
Tpringle	>UV7.culQui	R-type	Arthropoda	Culex	quinquefasciatus	mosquito	XM_001861603
Tpringle	>UV7.droAna	R-type	Arthropoda	Drosophila	ananassae	fruitfly	XP_001956024
Tpringle	>UV7.droMel	R-type	Arthropoda	Drosophila	melanogaster	fruitfly	NP_524035
Tpringle	>UV7.droMoj	R-type	Arthropoda	Drosophila	mojavensis	fruitfly	XP_002007363.1
Tpringle	>UV7.droPse	R-type	Arthropoda	Drosophila	pseudoobscura	fruitfly	XP_002134833
Tpringle	>UV7.droWil	R-type	Arthropoda	Drosophila	willistoni	fruitfly	XP_002068301.1
Tpringle	>UV7.droYak	R-type	Arthropoda	Drosophila	yakuba	fruitfly	XP_002094554

Tpringle	>UV7.pedHum	R-type	Arthropoda	Pediculus	humanus	louse	AAZO01007270
Tpringle	>UV7.rhoPro	R-type	Arthropoda	Rhodnius	prolixus	kissing bug	Genome
Tpringle	>UV7a.acyPis	R-type	Arthropoda	Acyrthosiphon	pisum	pea aphid	XP_001950416.1
Tpringle	>UV7b.acyPis	R-type	Arthropoda	Acyrthosiphon	pisum	pea aphid	XP_001944926.1
Tpringle	>UVB.acyPis	R-type	Arthropoda	Acyrthosiphon	pisum	pea aphid	XP_001951588.1
Tpringle	>UVB.anoGam	R-type	Arthropoda	Anopheles	gambiae	mosquito	XP_319247.1
Tpringle	>UVB.apiMel	R-type	Arthropoda	Apis	mellifera	bee	AF004168
Tpringle	>UVB.diaNig	R-type	Arthropoda	Dianemobius	nigrofasciatus	cricket	BAF45422
Tpringle	>UVB.manSex	R-type	Arthropoda	Manduca	sexta	moth	AAD11966.1
Tpringle	>UVB.megVic	R-type	Arthropoda	Megoura	viciae	vetch aphid	AAG17120
Tpringle	>UVB.nasVit	R-type	Arthropoda	Nasonia	vitripennis	jewel wasp	XP_001604622.1
UMBC	>CEPH.Acusub	R-type	Mollusca	Alloteuthis	subulata	squid	Z49108

UMBC	>CEPH.Eupsco1	R-type	Mollusca	Euprymna	scolopes	ceph	ACB05673.1
UMBC	>CEPH.Eupsco2	R-type	Mollusca	Euprymna	scolopes	ceph	ACB05672.1
UMBC	>CEPH.Lolfor	R-type	Mollusca	Loligo	forbesi	squid	CAA40108.1
UMBC	>CEPH.Lolpea	R-type	Mollusca	Loligo	pealei	squid	AY450853
UMBC	>CEPH.Octdof	R-type	Mollusca	Enteroctopus	dofleini	octopus	CAA30644.1
Tpringle	>LWS.todPac	R-type	Mollusca	Todarodes	pacificus	squid	X70498
Tpringle	>MEL1.capCap	R-type	Annelida	Capitella	capitata	polychaete worm	Genome
Tpringle	>MEL1.schMan	R-type	Platyhelminthes	Schistosoma	mansoni	trematode worm	AF155134
Tpringle	>MEL1.schMed	R-type	Platyhelminthes	Schmidtea	mediterranea	planaria	AF112361
Tpringle	>MEL2.schMan	R-type	Platyhelminthes	Schistosoma	mansoni	trematode worm	CD096414
Tpringle	>MEL1.bosTau	R-type	Chordata	Bos	taurus	cow	NP_001179328.1
Tpringle	>MEL1.calJac	R-type	Chordata	Callithrix	jacchus	marmoset	Genome

Tpringle	>MEL1.calMil	R-type	Chordata	Callorhinchus	milii	elephantfish	Genome
Tpringle	>MEL1.canFam	R-type	Chordata	Canis	familiaris	dog	XP_853735.1
Tpringle	>MEL1.danRer	R-type	Chordata	Danio	rerio	zebrafish	Genome
Tpringle	>MEL1.danRer2	R-type	Chordata	Danio	rerio	zebrafish	Genome
Tpringle	>MEL1.equCab	R-type	Chordata	Equus	caballus	horse	XP_001501116.2
Tpringle	>MEL1.eriEur	R-type	Chordata	Erinaceus	europaeus	hedgehog	Genome
Tpringle	>MEL1.felCat	R-type	Chordata	Felis	catus	cat	AY382594
Tpringle	>MEL1.galGal	R-type	Chordata	Gallus	gallus	chicken	NP_001038118.1
Tpringle	>MEL1.gasAcu	R-type	Chordata	Gasterosteus	aculeatus	stickleback	Genome
Tpringle	>MEL1.gorGor	R-type	Chordata	Gorilla	gorilla	gorilla	Genome
Tpringle	>MEL1.homSap	R-type	Chordata	Homo	sapiens	human	NM_033282
Tpringle	>MEL1.lotGig	R-type	Mollusca	Lottia	gigantea	limpet	FC774055

Tpringle	>MEL1.micMur	R-type	Chordata	Microcebus	murinus	mouse lemur	Genome
Tpringle	>MEL1.monDom	R-type	Chordata	Monodelphis	domestica	opossum	Genome
Tpringle	>MEL1.musMus	R-type	Chordata	Mus	musculus	mouse	AF147789
Tpringle	>MEL1.nanEhr	R-type	Chordata	Nannospalax	ehrenbergi	molerat	AM748539
Tpringle	>MEL1.ornAna	R-type	Chordata	Ornithorhynchus	anatinus	platypus	Genome
Tpringle	>MEL1.oryLat	R-type	Chordata	Oryzias	latipes	medaka	Genome
Tpringle	>MEL1.otoGar	R-type	Chordata	Otolemur	garnettii	lemur	Genome
Tpringle	>MEL1.panTro	R-type	Chordata	Pan	troglodytes	chimp	Genome
Tpringle	>MEL1.patYes	R-type	Mollusca	Patinopecten	yessoensis	scallop	AB006454
Tpringle	>MEL1.petMar	R-type	Chordata	Petromyzon	marinus	lamprey	Genome
Tpringle	>MEL1.phoSun	R-type	Chordata	Phodopus	sungorus	hamster	AY726733
Tpringle	>MEL1.ponAbe	R-type	Chordata	Pongo	abelii	orang	Genome

Tpringle	>MEL1.proCap	R-type	Chordata	Procavia	capensis	rock hyrax	Genome
Tpringle	>MEL1.pteVam	R-type	Chordata	Pteropus	vampyrus	macrobat	Genome
Tpringle	>MEL1.ratNor	R-type	Chordata	Rattus	norvegicus	rat	AY072689
Tpringle	>MEL1.rheMac	R-type	Chordata	Rhesus	macaca	rhesus	Genome
Tpringle	>MEL1.sepOff	R-type	Mollusca	Sepia	officinalis	cuttlefish	AF000947
Tpringle	>MEL1.smiCra	R-type	Chordata	Sminthopsis	crassicaudata	dunnart	DQ383281
Tpringle	>MEL1.susScr	R-type	Chordata	Sus	scrofa	pig	Genome
Tpringle	>MEL1.taeGut	R-type	Chordata	Taeniopygia	guttata	finch	Genome
Tpringle	>MEL1.takRub	R-type	Chordata	Takifugu	rubripes	fugu	Genome
UMBC	>MEL1.Xenlae	R-type	Chordata	Xenopus	laevis	frog	ABD37674.1
Tpringle	>MEL1.xenTro	R-type	Chordata	Xenopus	tropicalis	frog	XP_002937616.1
Tpringle	>MEL2.anoCar	R-type	Chordata	Anolis	carolinensis	lizard	Genome

Tpringle	>MEL2.danRer	R-type	Chordata	Danio	rerio	zebrafish	Genome
Tpringle	>MEL2.galGal	R-type	Chordata	Gallus	gallus	chicken	AY882944
Tpringle	>MEL2.gasAcu	R-type	Chordata	Gasterosteus	aculeatus	stickleback	Genome
Tpringle	>MEL2.tetNig	R-type	Chordata	Tetraodon	nigroviridis	pufferfish	Genome
Tpringle	>MEL2.xenLae	R-type	Chordata	Xenopus	laevis	frog	Genome
Tpringle	>MELmop.braBel	R-type	Chordata	Branchiostoma	belcheri	amphioxus	AB205400
Tpringle	>MELmop.braFlo	R-type	Chordata	Branchiostoma	floridae	amphioxus	Genome
Tpringle	>MEL1.aplCal	R-type	Mollusca	Aplysia	californica	sea hare	AASC01108363
Tpringle	>MEL1.cioInt	R-type	Chordata	Ciona	intestinalis	tunicate	AABS01000008
Tpringle	>MEL1.cioSav	R-type	Chordata	Ciona	savignyi	tunicate	Genome
Tpringle	>MEL2.lotGig	R-type	Mollusca	Lottia	gigantea	limpet	Genome
Tpringle	>MEL6.braBel	R-type	Chordata	Branchiostoma	belcheri	amphioxus	AB050611

Tpringle	>MEL6.braFlo	R-type	Chordata	Branchiostoma	floridae	amphioxus	XP_002586119.1
Tpringle	>MEL1.dapPul	R-type	Arthropoda	Daphnia	pulex	water flea	Genome
Gen-Bank	>Nvop1	Cnidops	Cnidaria	Nematostella	vectensis	anemone	FAA00408.1
Gen-Bank	>Nvop2	Cnidops	Cnidaria	Nematostella	vectensis	anemone	FAA00400.1
Gen-Bank	>Nvop3	Cnidops	Cnidaria	Nematostella	vectensis	anemone	FAA00410.1
Gen-Bank	>Nvop4	Cnidops	Cnidaria	Nematostella	vectensis	anemone	FAA00409.1
Gen-Bank	>Nvop5	Cnidops	Cnidaria	Nematostella	vectensis	anemone	FAA00407.1
Gen-Bank	>Nvop6	Cnidops	Cnidaria	Nematostella	vectensis	anemone	FAA00406.1
Gen-Bank	>Nvop7	Cnidops	Cnidaria	Nematostella	vectensis	anemone	FAA00405.1
Gen-Bank	>Nvop8	Cnidops	Cnidaria	Nematostella	vectensis	anemone	FAA00404.1
Gen-Bank	>Nvop9	Cnidops	Cnidaria	Nematostella	vectensis	anemone	FAA00403.1
Gen-Bank	>Nvop11	Cnidops	Cnidaria	Nematostella	vectensis	anemone	FAA00401.1

Gen-Bank	>Nvop12	Cnidops	Cnidaria	Nematostella	vectensis	anemone	FAA00399.1
Gen-Bank	>Nvop13	Cnidops	Cnidaria	Nematostella	vectensis	anemone	FAA00398.1
Gen-Bank	>Nvop14	Cnidops	Cnidaria	Nematostella	vectensis	anemone	FAA00397.1
Gen-Bank	>Nvop15	Cnidops	Cnidaria	Nematostella	vectensis	anemone	FAA00396.1
Gen-Bank	>Nvop17	Cnidops	Cnidaria	Nematostella	vectensis	anemone	FAA00395.1
Gen-Bank	>Nvop19	Cnidops	Cnidaria	Nematostella	vectensis	anemone	FAA00412.1
Gen-Bank	>Nvop21	Cnidops	Cnidaria	Nematostella	vectensis	anemone	FAA00393.1
Gen-Bank	>Nvop22	Cnidops	Cnidaria	Nematostella	vectensis	anemone	FAA00392.1
Gen-Bank	>Nvop23	Cnidops	Cnidaria	Nematostella	vectensis	anemone	FAA00391.1
Gen-Bank	>Nvop25	Cnidops	Cnidaria	Nematostella	vectensis	anemone	FAA00389.1
Gen-Bank	>Nvop26	Cnidops	Cnidaria	Nematostella	vectensis	anemone	FAA00388.1
Gen-Bank	>Nvop27	Cnidops	Cnidaria	Nematostella	vectensis	anemone	FAA00387.1

Gen-Bank	>Nvop28	Cnidops	Cnidaria	Nematostella	vectensis	anemone	FAA00386.1
Gen-Bank	>Nvop29	Cnidops	Cnidaria	Nematostella	vectensis	anemone	FAA00385.1
Gen-Bank	>Nvop30	Cnidops	Cnidaria	Nematostella	vectensis	anemone	FAA00384.1
Gen-Bank	>Nvop31	Cnidops	Cnidaria	Nematostella	vectensis	anemone	FAA00383.1
Tpringle	>Caryb	Cnidops	Cnidaria	Carybdea	rastonii	sea wasp	BAG80696
Tpringle	>Tcop18	Cnidops	Cnidaria	Tripedalia	cystophora	box jelly	EU310498
Gen-Bank	>Tcop1	Cnidops	Cnidaria	Tripedalia	cystophora	box jelly	JQ968432
Gen-Bank	>Tcop2	Cnidops	Cnidaria	Tripedalia	cystophora	box jelly	JQ968431
Gen-Bank	>Tcop3	Cnidops	Cnidaria	Tripedalia	cystophora	box jelly	JQ968430
Gen-Bank	>Tcop4	Cnidops	Cnidaria	Tripedalia	cystophora	box jelly	JQ968429
Gen-Bank	>Tcop5	Cnidops	Cnidaria	Tripedalia	cystophora	box jelly	JQ968428
Gen-Bank	>Tcop6	Cnidops	Cnidaria	Tripedalia	cystophora	box jelly	JQ968427

Gen-Bank	>Tcop7	Cnidops	Cnidaria	Tripedalia	cystophora	box jelly	JQ968426
Gen-Bank	>Tcop8	Cnidops	Cnidaria	Tripedalia	cystophora	box jelly	JQ968425
Gen-Bank	>Tcop9	Cnidops	Cnidaria	Tripedalia	cystophora	box jelly	JQ968424
Gen-Bank	>Tcop10	Cnidops	Cnidaria	Tripedalia	cystophora	box jelly	JQ968423
Gen-Bank	>Tcop11	Cnidops	Cnidaria	Tripedalia	cystophora	box jelly	JQ968422
Gen-Bank	>Tcop12	Cnidops	Cnidaria	Tripedalia	cystophora	box jelly	JQ968421
Gen-Bank	>Tcop13	Cnidops	Cnidaria	Tripedalia	cystophora	box jelly	JQ968420
Gen-Bank	>Tcop14	Cnidops	Cnidaria	Tripedalia	cystophora	box jelly	JQ968419
Gen-Bank	>Tcop15	Cnidops	Cnidaria	Tripedalia	cystophora	box jelly	JQ968418
Gen-Bank	>Tcop16	Cnidops	Cnidaria	Tripedalia	cystophora	box jelly	JQ968417
Gen-Bank	>Tcop17	Cnidops	Cnidaria	Tripedalia	cystophora	box jelly	JQ968416
Gen-Bank	>OG.TSHR	TSHR		Homo	sapiens	human	AAB87990

Gen-Bank	>OG.ADORA3	A3 adenosine receptor		Homo	sapiens	human	AAA16365.1
NCBI	>OG.TRHR	TRHR		Homo	sapiens	human	NP_003292.1
Gen-Bank	>OG.ADRA1D	alpha-1A-adrenergic receptor		Homo	sapiens	human	AAA35496.1
NCBI	>OG.GPR161	G-protein coupled receptor 161 isoform 2		Homo	sapiens	human	NP_722561.1
NCBI	>OG.PRLHR	prolactin-releasing peptide receptor		Homo	sapiens	human	NP_004239.1
NCBI	>OG.NPY1R	neuropeptide Y receptor type 1		Homo	sapiens	human	NP_000900.1
NCBI	>OG.PPYR1	neuropeptide Y receptor type 4		Homo	sapiens	human	NP_005963.3
NCBI	>OG.GPR19	G-protein coupled receptor 19		Homo	sapiens	human	NP_006134.1
Gen-Bank	>OG.QRFPR	QRFP receptor		Homo	sapiens	human	BAC98938.1
Gen-Bank	>OG.NMUR2	neuromedin U receptor 2		Homo	sapiens	human	AAF82755.1
Gen-Bank	>OG.TACR2	neurokinin-2 receptor		Homo	sapiens	human	AAB05897.1

Gen-Bank	>OG.HCRTR1	orexin receptor-1		Homo	sapiens	human	AAC39601.1
NCBI	>OG.P2RY8	P2Y purinoceptor 8		Homo	sapiens	human	NP_835230.1
NCBI	>OG.CYSLTR1	cysteinyl leukotriene receptor 1		Homo	sapiens	human	NP_006630.1
Gen-Bank	>OG.GPR17	G protein-coupled receptor 17		Homo	sapiens	human	AEP43758.1
NCBI	>OG.BDKRB2	B2 bradykinin receptor		Homo	sapiens	human	NP_000614.1
NCBI	>OG.CCR4	C-C chemokine receptor type 4		Homo	sapiens	human	NP_005499.1
Gen-Bank	>OG.GALR1	galanin receptor		Homo	sapiens	human	AAC51936.1
Gen-Bank	>OG.OPRM1	opioid receptor, mu 1		Homo	sapiens	human	CAI20458.1
NCBI	>OG.OPRL1	nociceptin receptor		Homo	sapiens	human	NP_000904.1
NCBI	>OG.SSTR1	somatostatin receptor type 1		Homo	sapiens	human	NP_001040.1

Table 2 - Dataset sequences

List of sequences used for phylogenetic analysis.
